# Fe-catalyzed esterification of amides *via* C–N bond activation†

**DOI:** 10.1039/c7ra12152k

**Published:** 2018-01-25

**Authors:** Xiuling Chen, Siying Hu, Rongxing Chen, Jian Wang, Minghu Wu, Haibin Guo, Shaofa Sun

**Affiliations:** Hubei University Of Science and Technology China

## Abstract

An efficient Fe-catalyzed esterification of primary, secondary, and tertiary amides with various alcohols for the preparation of esters was performed. The esterification process was accomplished with FeCl_3_·6H_2_O, which is a stable, inexpensive, environmentally friendly catalyst with high functional group tolerance.

## Abstract 2

An efficient Fe-catalyzed esterification of primary, secondary, and tertiary amides with various alcohols was performed. Esterification was accomplished with inexpensive, environmentally friendly FeCl_3_·6H_2_O, and with high functional group tolerance

## Graphical abstract

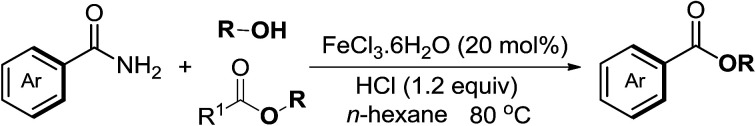

The amide bond is not only employed as an important natural peptide skeleton in biological systems, but also as a versatile functional group in organic transformations.^1,2^ Among amide transformations,^3,4^ transamidation and esterification of amides have attracted widespread attention from organic chemists.^5–11^ In contrast to transamidation of amides, esterification of amides is relatively difficult because of the low nucleophilicity of alcohols compared with amines.^12^ To overcome this shortcoming, various processes for the esterification of amides have been developed, such as using an activating agent,^6^ employing twisted amides,^7^ and forming intramolecular assisted groups.^8^ For increasing the synthetic flexibility of esterification of amides, new catalytic systems Zn(OTf)_2_ and Sc(OTf)_3_ were developed by Mashima and Williams for the esterification of amides.^9^ Shimizu *et al.* reported CeO_2_ catalyzed esterification of amides.^10^ Off late, progress of the significant Ni-catalyzed esterification of amides has gained precedence.^11^ Although this approach produces satisfactory yield, the drawbacks such as the use of an expensive catalyst, generation of reagent waste, high temperature and the limited scope of the substrate still persists. Herein, under mild reaction conditions, we report a novel and efficient Fe-catalyzed esterification of amides for the synthesis of esters (eqn (1)). Compared to conventional methods, this esterification procedure is distinguished by using a stable, inexpensive, environment-friendly catalyst, *i.e.*, FeCl_3_·6H_2_O with a low toxicity solvent. The catalytic system has wide functional group compatibility. The reactions of several primary, secondary, and tertiary amides with various alcohols have been well tolerated in this process. Moreover, esters were also as effective as esterification reagents for the esterification of amides by the acyl–acyl exchange process. In the course of our present study, a Co-catalyzed amide C–N cleavage to form esters was reported by Danoun *et al.*^13^ However, an additional additive (Bipy) and Mn (3 equiv.) were needed and the scope of the reaction was limited to only tertiary amides; primary or secondary amides were not compatible with this catalytic oxidation system.1
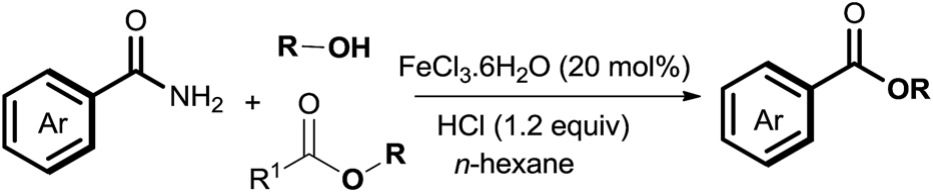


We initially selected benzamide 1a and ethanol 2a as model substrates to screen the reaction conditions, and the results are summarized in Table 1. First, in the presence of FeCl_2_, ethyl benzoate 3a was afforded in 31% yield (Table 1, entry 1). Next, our catalyst-screening results showed that various iron complexes such as FeCl_3_, FeBr_3_, FeSO_4_·7H_2_O, FeCl_3_·6H_2_O, and Fe(NO_3_)_3_·9H_2_O could catalyze the reaction (Table 1, entries 2–6), and FeCl_3_·6H_2_O was the best catalyst for this reaction as ethyl benzoate 3a was afforded in 35% yield (Table 1, entry 6). Other metal complexes such as CuCl_2_, PdCl_2_, and NiCl_2_·6H_2_O did not improve the efficiency of this transformation (Table 1, entries 7–9). In the absence of an iron complex, this reaction could not work and the product was not detected (Table 1, entry 10). Next, in the presence of FeCl_3_·6H_2_O, the influence of additives on the reaction was investigated (Table 1, entries 11–18). Among the tested additives, the stronger bidentate ligands such as 1,10-phenanthroline or 2,2-bipyridine did not show any efficiency, while hydrochloric acid gave the best reactive activity as ethyl benzoate 3a was obtained in 91% yield (Table 1, entry 18). In general, H_2_SO_4_ or HNO_3_ was used as a catalyst for esterification; however, they did not efficiently promote the reaction under the present system (Table 1, entries 16–17). In the absence of FeCl_3_·6H_2_O, the product 3a was afforded in 35% yield (Table 1, entry 19). For the solvents investigated (Table 1, entries 20–23), *n*-hexane was the best choice (Table 1, entry 18).

**Table 1:** Optimization of the reaction conditions^a^

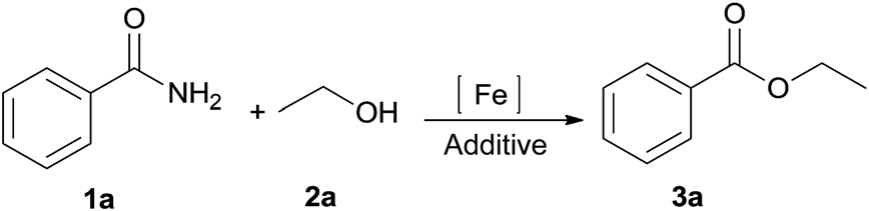			
Entry	Catalyst (20%)	Additive (40%)	Yield^b^
1	FeCl_2_	—	31
2	FeCl_3_	—	31
3	FeBr_3_	—	25
4	FeSO_4_·7H_2_O	—	20
5	Fe(NO_3_)_3_·9H_2_O	—	20
6	FeCl_3_·6H_2_O	—	35
7	CuCl_2_	—	Trace
8	PdCl_2_	—	Trace
9	Ni_2_Cl·6H_2_O	—	Trace
10	—	—	—
12	FeCl_3_·6H_2_O	Pyridine	Trace
13	FeCl_3_·6H_2_O	1,10-Phenanthroline	Trace
14	FeCl_3_·6H_2_O	Formic acid	Trace
15	FeCl_3_·6H_2_O	l-Proline	Trace
16	FeCl_3_·6H_2_O	H_2_SO_4_	22
17	FeCl_3_·6H_2_O	HNO_3_	25
18	FeCl_3_·6H_2_O	HCl	91
19	—	HCl	35
20^c^	FeCl_3_·6H_2_O	HCl	Trace
21^d^	FeCl_3_·6H_2_O	HCl	36
22^e^	FeCl_3_·6H_2_O	HCl	28
23^f^	FeCl_3_·6H_2_O	HCl	40

a Reaction conditions: 1a (0.2 mmol), 2a (0.1 mL), catalyst (0.04 mmol, 20 mol%), H_2_SO_4_ (concentrated, 0.24 mmol), HNO_3_ (concentrated, 0.24 mmol), HCl (0.24 mmol, 36–38%), *n*-hexane (1.0 mL), 80 °C, 14 h.

b GC yield using hexadecane as internal standard.

c DMF was used as a solvent.

d 1,4-Dioxane was used as solvent.

e Toluene was used as solvent.

f CH_3_CN was used as solvent.

The substrate scope of esterification with alcohols was investigated under the optimized reaction conditions. As shown in Table 2, esterification of benzamide by primary alcohols, secondary alcohols, tertiary alcohols, and ring alcohols took place smoothly, and the corresponding esters were afforded in good to high yields. When primary alcohols 2a, 2b, 2c were used as substrates, affording the ester products 3a–3c in high yields, it should be noted that the reactivity of the alcoholysis of amides was independent of the alkyl chain length (Table 2, entries 1–3). Secondary alcohols 2d and tertiary alcohols 2e also worked effectively as the substrates, producing the esters in 81–91% yield (Table 2, entries 4–5). Treating 1a with cyclohexanol 2f could afford the ester 3f in 80% yield (Table 2, entry 6). Alcoholysis products 3g and 3h were obtained from corresponding alcohols (Table 2, entries 7–8). Substrate alcohol having trifluoromethyl also participated in this catalytic reaction to form the ester 3i in high yield (Table 2, entry 9). Moreover, when ethane-1,2-diol was treated with 1a, only one product 3j was obtained (Table 2, entry 10).

**Table 2:** Esterification of primary amide with various alcohols^a^

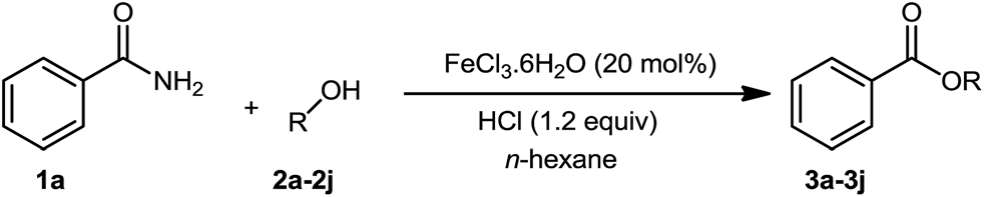			
Entry	Alcohol	Products	Yield^b^
1	Ethanol 2a	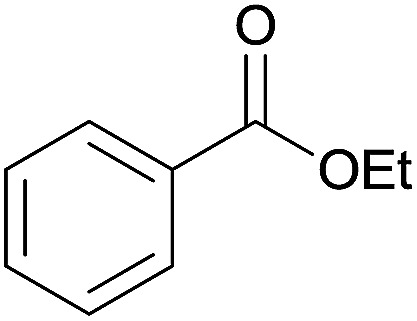	3a, 85%
2	*n*-Propanol 2b	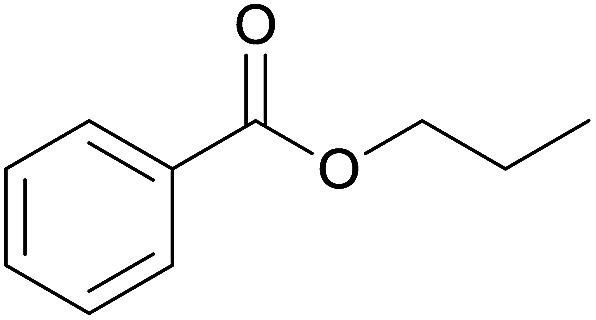	3b, 88%
3	*n*-Octanol 2c	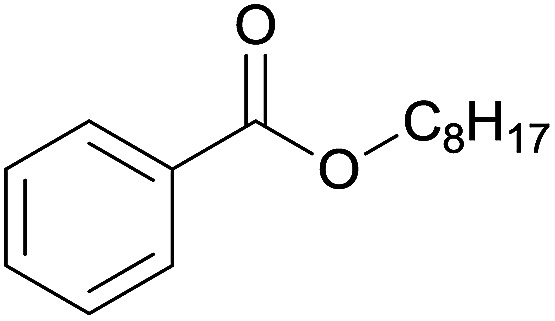	3c, 82%
4	*i*-Propanol 2d	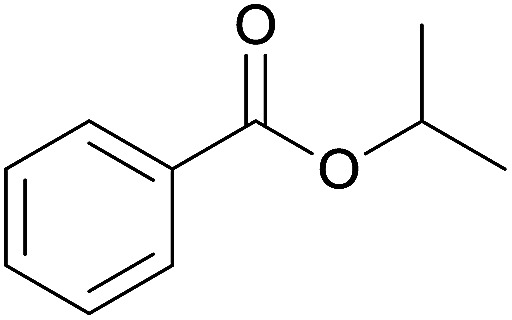	3d, 91%
5	*t*-Butyl alcohol 2e	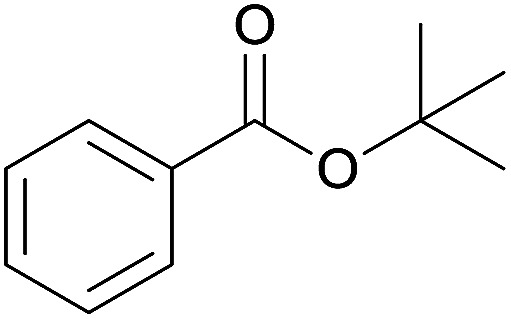	3e, 81%
6	Cyclohexanol 2f	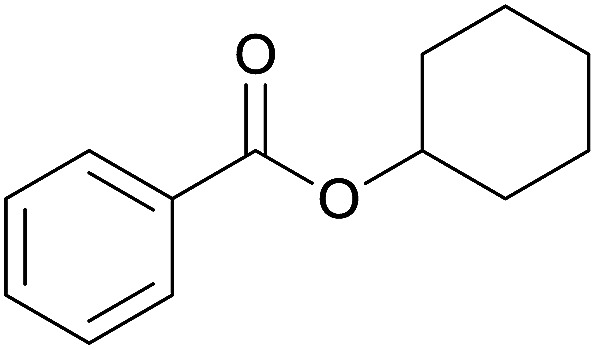	3f, 80%
7	Phenethanol 2g	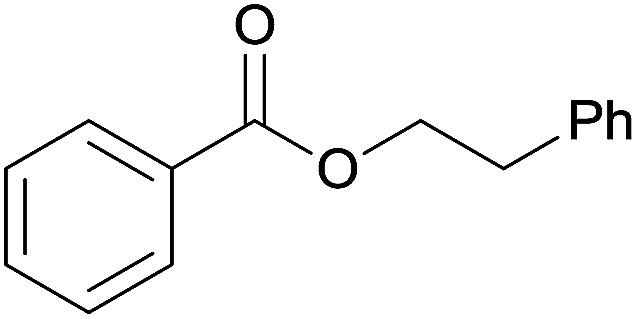	3g, 83%
8	Phenethanol 2h	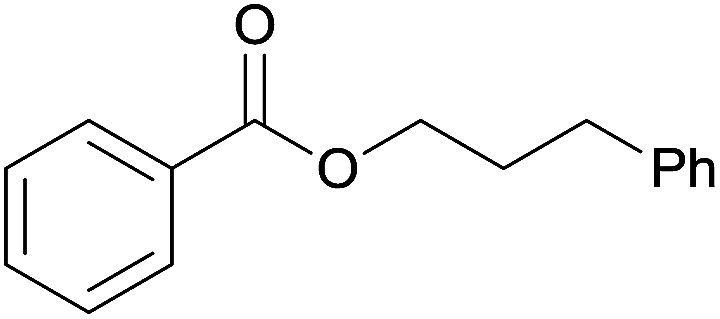	3h, 81%
9	Trifluoroethanol 2i	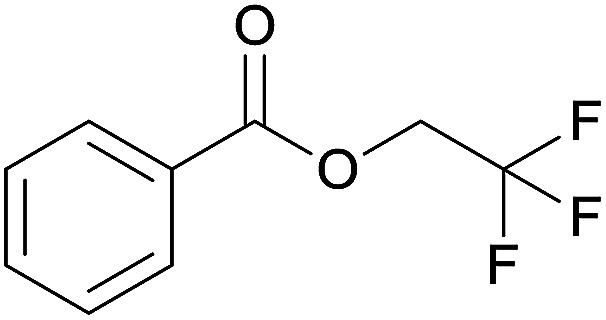	3i, 81%
10	Ethane-1,2-diol 2j	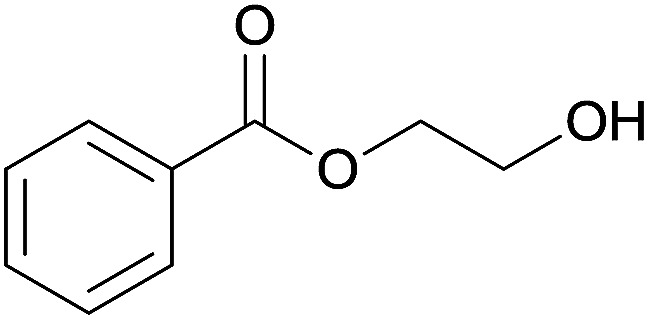	3j, 85%

a Reaction conditions: 1a (0.2 mmol), 2a (0.1 mL), 2b–2j (0.24 mmol), FeCl_3_·6H_2_O (0.04 mmol, 20 mol%), HCl (0.24 mmol, 36–38%), *n*-hexane (1.0 mL), 80 °C, 14 h.

b Isolated yield.

We then examined the generality of amides and the results are compiled in Table 3. Under the optimized conditions, both electron-rich and electron-deficient group substituted benzamide could undergo C–N bond cleavage and esterification by ethanol to afford the corresponding ester products in high yields (Table 3, entries 1–7). Various functional groups such as alkoxy, hydroxyl, amino, halide, and nitro remained intact during the reaction. 2-Naphthamide also served as an efficient substrate and could react with 2a, furnishing the ester 3r in 86% yield. Interestingly, heteroaryl substituted esters 3s and 3t were obtained from the reaction of 2-thiophenecarboxamide and 3-indoleacetamide with 2a using the present reaction conditions, indicating that this method will be an efficient approach to introduce an aromatic heterocycle into functional molecules. Substrates containing alkynes such as cinnamamide (1l) could also react with ethanol smoothly to give an esterification product 3u with 88% yield. The reaction was not sensitive to the steric environment of the amide moiety; when *meta*- and *ortho*-substituted amides 1m and 1n were subjected to the reaction system, the corresponding ester products were obtained in high yields.

**Table 3:** Esterification of different amides with ethanol^a^

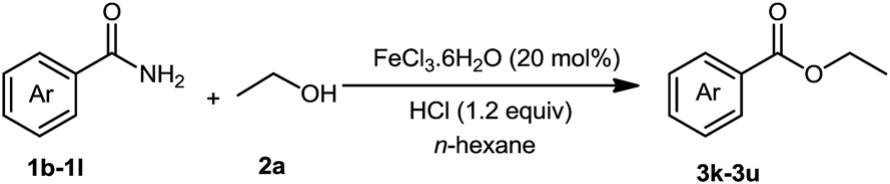		
Entry	Amides	Products and yield^b^
1	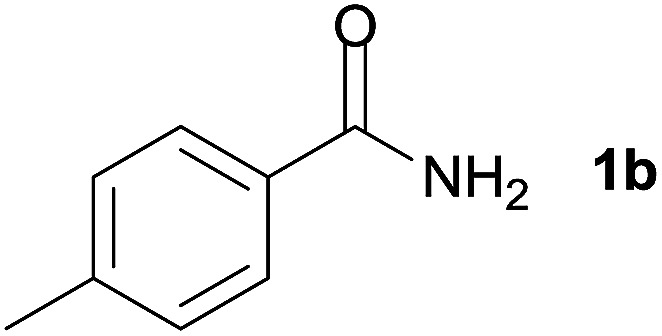	3k, 86%
2	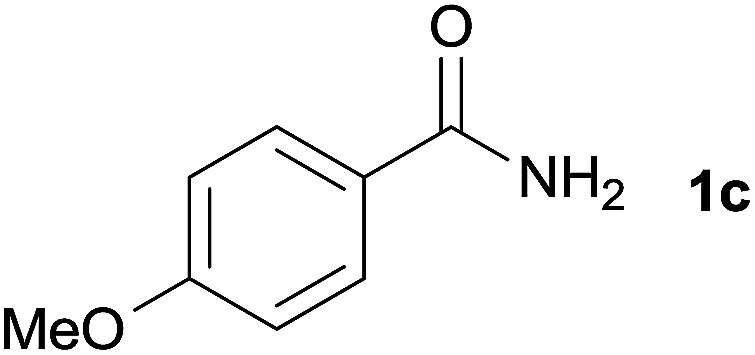	3l, 85%
3	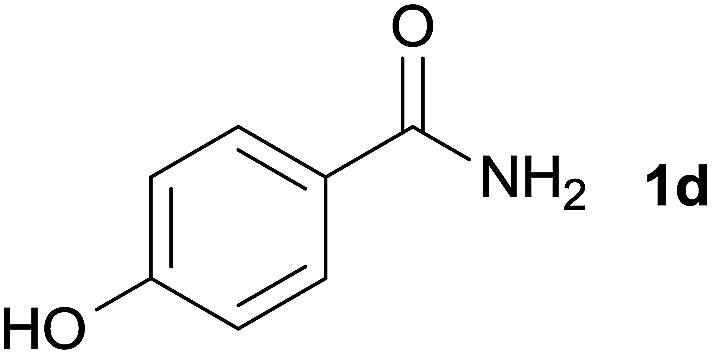	3m, 78%
4	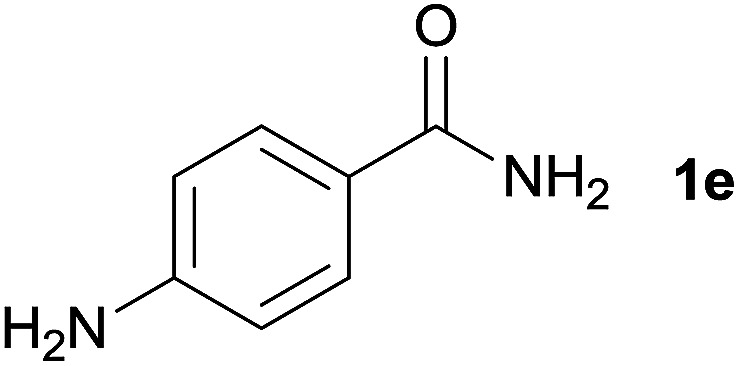	3n, 75%
5	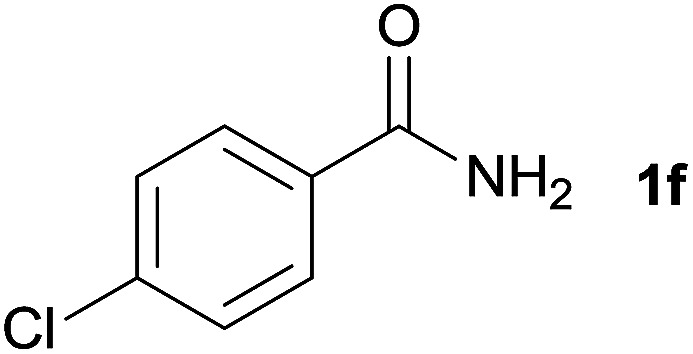	3o, 86%
6	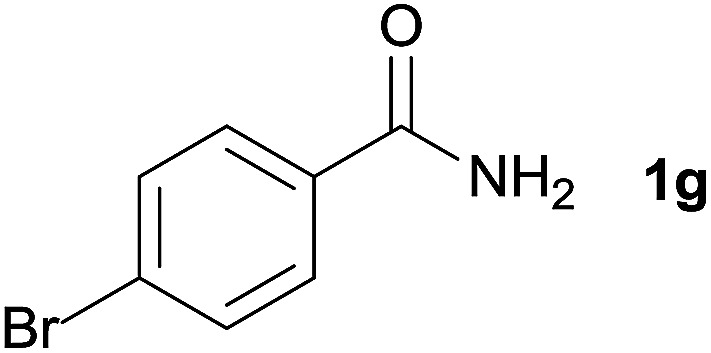	3p, 84%
7	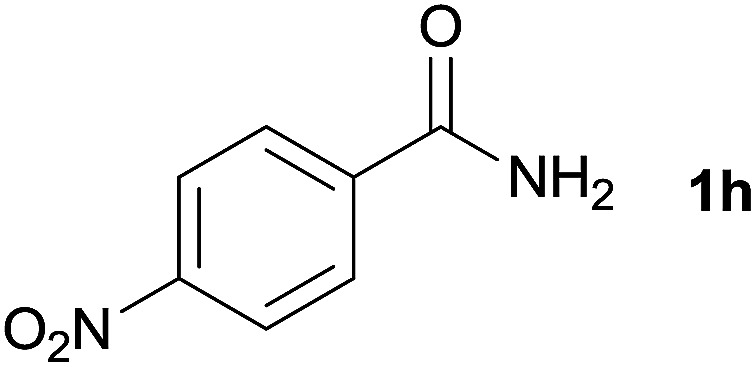	3q, 90%
8	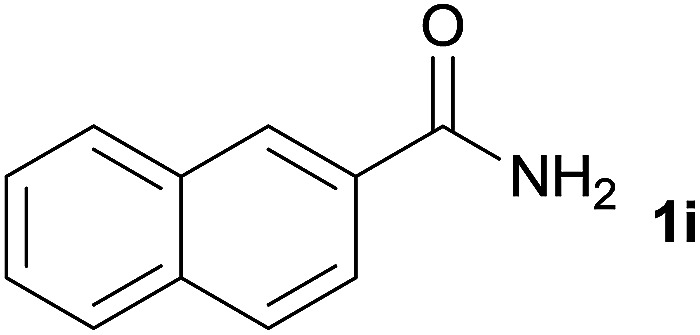	3r, 86%
9	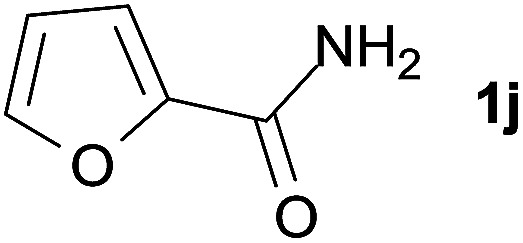	3s, 84%
10	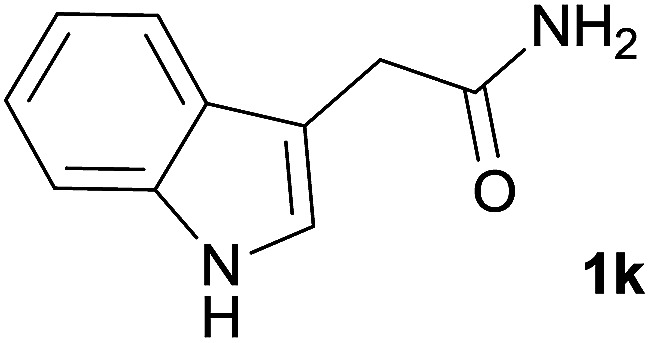	3t, 55%
11	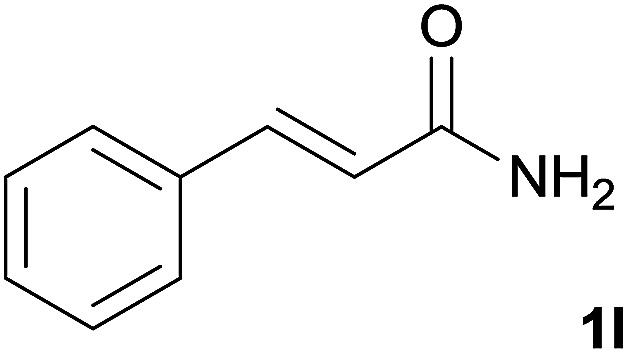	3u, 88%
12	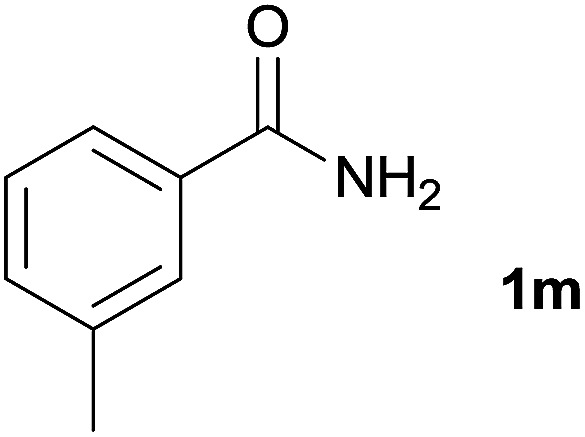	3v, 85%
13	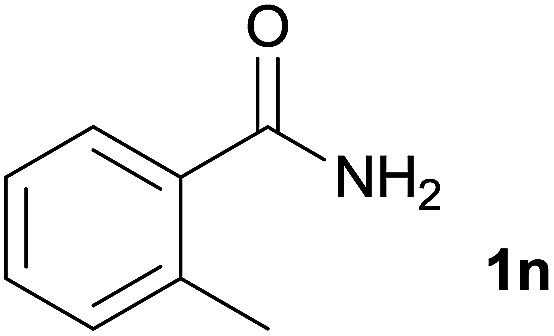	3w, 81%

a Reaction conditions: 1b–1n (0.2 mmol), 2a (0.1 mL), FeCl_3_·6H_2_O (0.04 mmol, 20 mol%), HCl (0.24 mmol, 36–38%), *n*-hexane (1.0 mL), 80 °C, 14 h.

b Isolated yield.

In addition to alcohols, esters were also used as substrates for esterification of amides through the acyl–acyl exchange process under the optimal reaction conditions.^14^ As shown in Table 4, in the current catalytic system, formate, acetate and benzoate also served as efficient substrates and could react with amides, furnishing the corresponding esters in high yield (Table 4, entries 1–7).

**Table 4:** Expanded substrate scope of amides and esters^a^

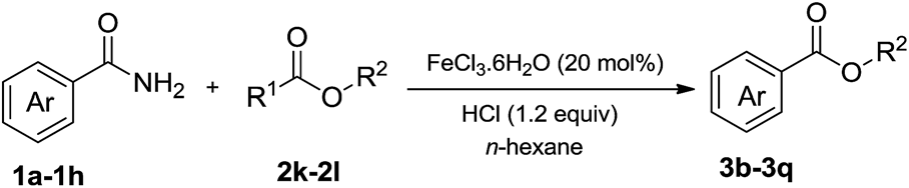			
Entry	Amides	Esters	Products and yield^b^
1	1a	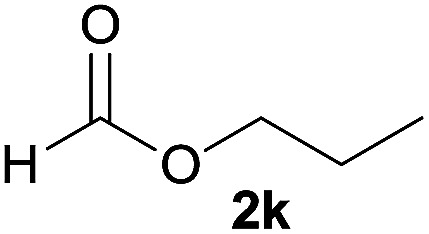	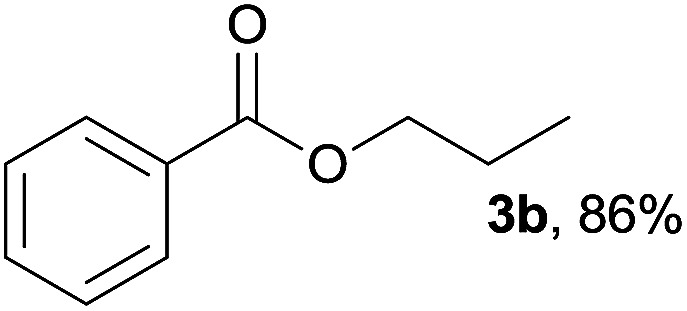
2	1a	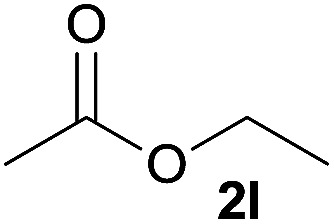	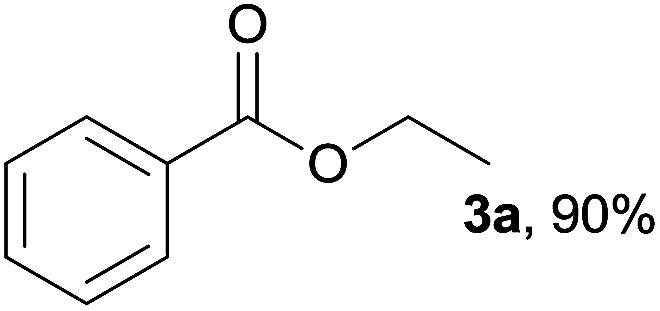
3	1a	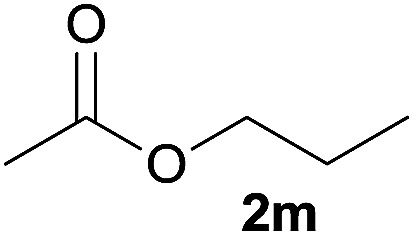	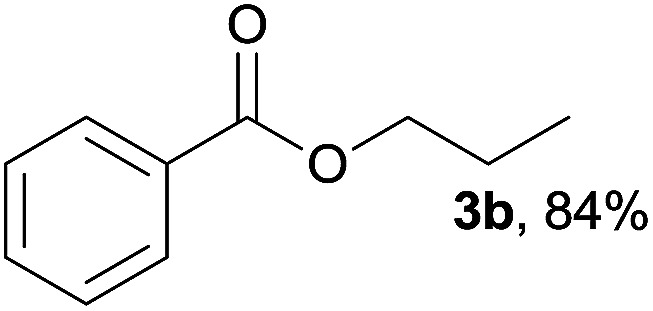
4	1a	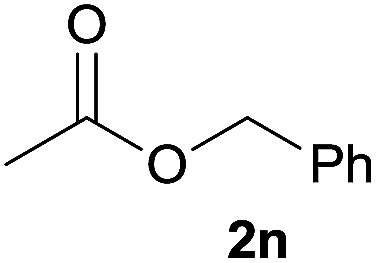	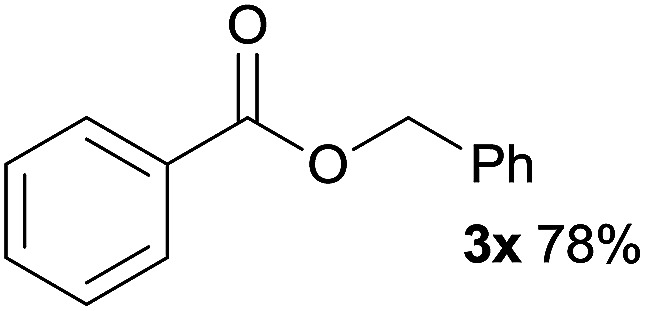
5	1b	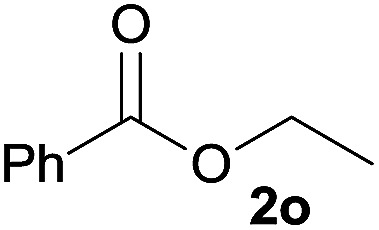	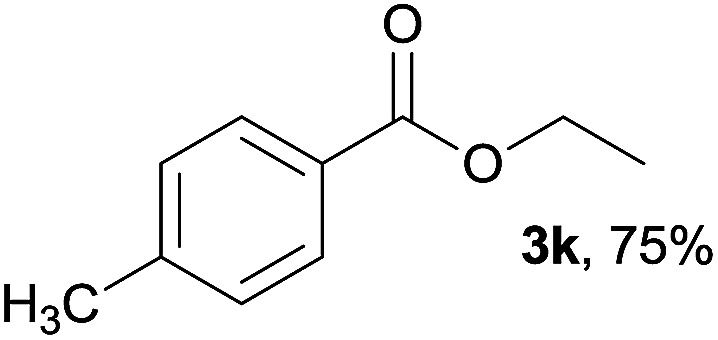
6	1c	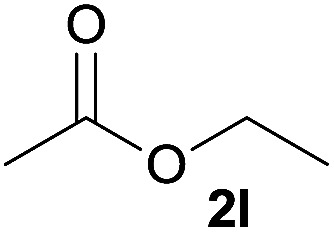	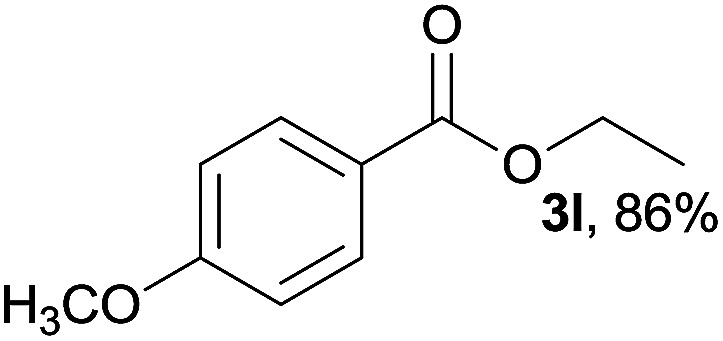
7	1h	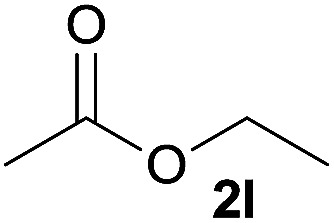	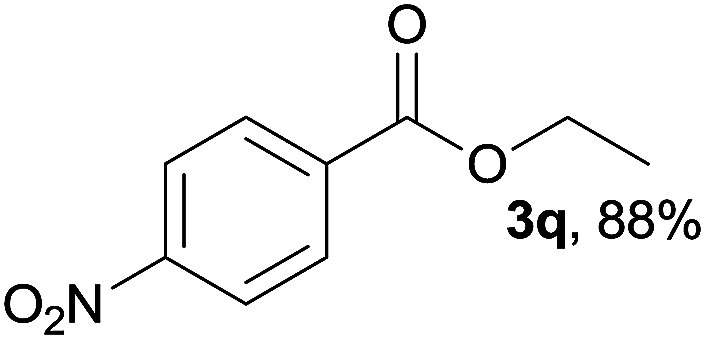

a Reaction conditions: 1a–1h (0.2 mmol), 2k, 2l (0.1 mL), 2m–2o (0.24 mmol), FeCl_3_·6H_2_O (0.04 mmol, 20 mol%), HCl (0.24 mmol, 36–38%), *n*-hexane (1.0 mL), 80 °C, 14 h.

b Isolated yield.

The substrate scope of this iron-catalyzed esterification of secondary and tertiary amides with alcohols was investigated. Remarkably, when a secondary amide, *viz.*, *N*-methylbenzamide (1o) and a tertiary amide, *viz.*, *N*,*N*-diethylbenzamide (1p) were used as substrates, the corresponding esters 3a and 3v were afforded in high yield (Scheme 1).

**Scheme 1:**
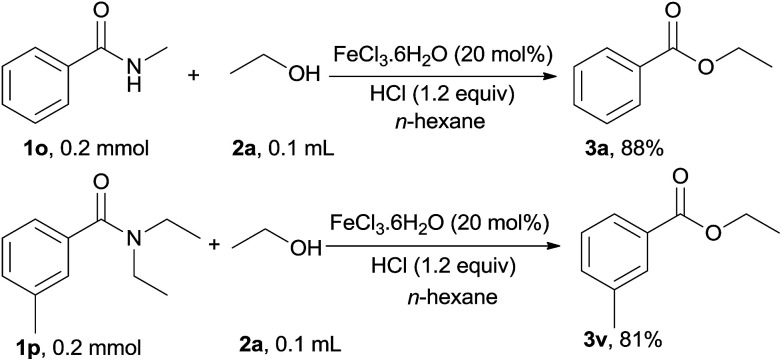
Reaction of secondary and tertiary amides 1o and 1p with ethanol.

In addition to aromatic primary, secondary and tertiary amides, the esterification of aliphatic primary, secondary and tertiary amides was also investigated under the optimal reaction conditions, the corresponding esters were afforded in good to excellent yield and the results are shown in Scheme 2. 2-Phenylacetamide (1q) participated in this catalytic reaction to form the ester 3y in 86% yield. *N*-Acetylaniline 1r and *N*,*N*-dimethylformamide 1s were also applicable to this catalytic system, and transformed into the corresponding esters 3z and 3z^1^ in 79% and 82% yield respectively.

**Scheme 2:**
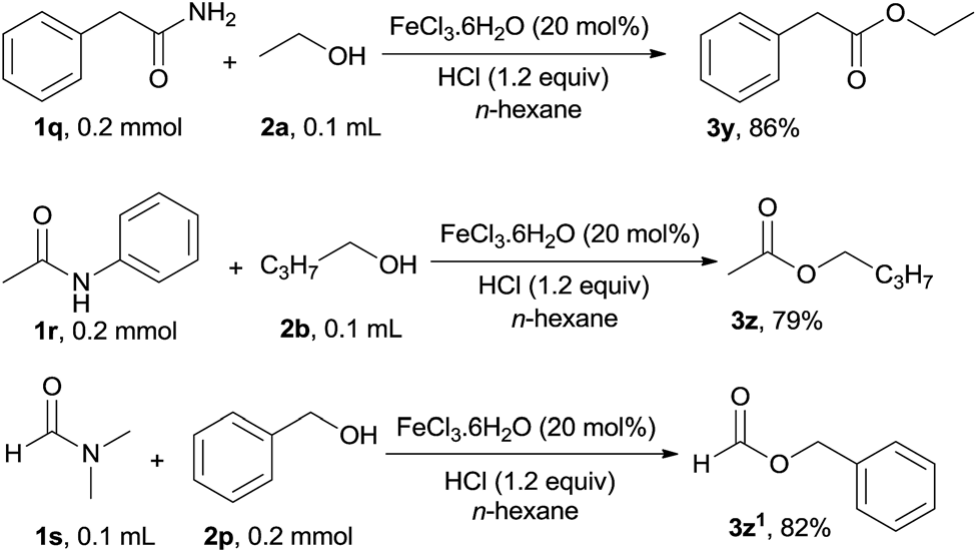
Reaction of aliphatic amides 1q, 1r and 1s with different alcohols.

To demonstrate the effect of iron salt in the current catalytic system, several control experiments were carried out as shown in Scheme 3. When 1 equivalent FeCl_3_·6H_2_O was used as the catalyst in the absence of HCl, 3a was obtained in 90% yield. This result indicated that the catalytic cycle of the iron salt was hindered in the present reaction conditions. When the stronger bidentate ligands such as 2,2-bipyridine was used, 3a was not obtained and trace amounts of 3a were detected on using ferrocene as the catalyst, indicating that the iron salt catalyst showed low efficiency in the presence of the stronger ligand. Thus, it was deduced that free Fe(ii or iii) was effective for this reaction.

**Scheme 3:**
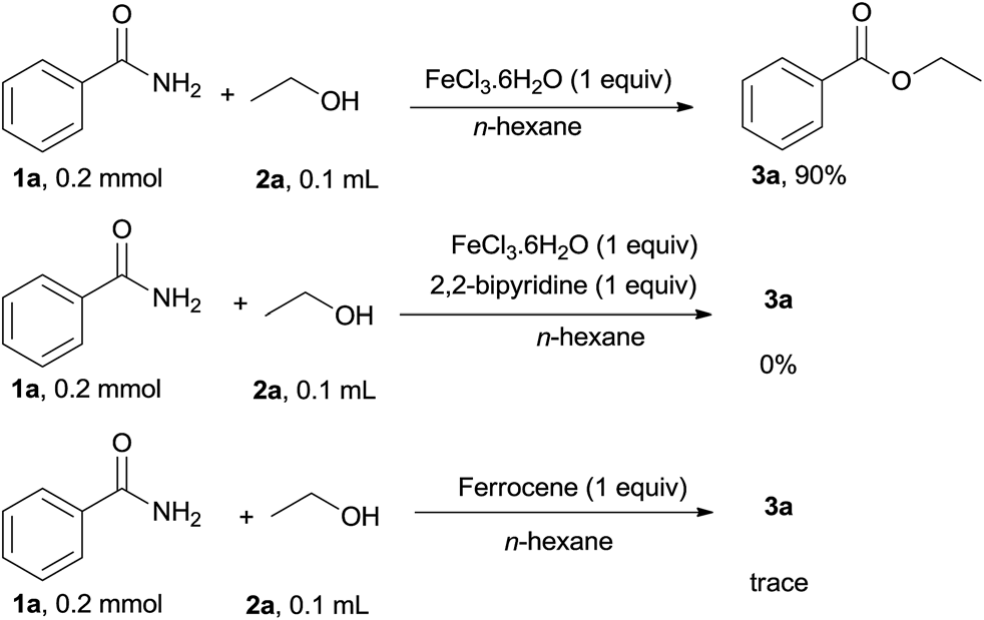
Control experiments.

According to the reported literature^5*d*^ and our experimental results, the catalytic reaction pathways for the Fe-catalyzed esterification of amides by alcohols are proposed as shown in Scheme 4. The first step is the generation of the amidate complex A, which can be formed from free Fe(iii) and amide 1. The complex A reacts with alcohol 2 to produce an unstable intermediate B. The interaction between the alcohol oxygen and the carbonyl results in cyclic intermediate C, which is in equilibrium with its isomer D. Intermediate E can also be produced from D*via* C–N bond cleavage. Through the reaction of HCl and a new molecule amide, intermediate E produces the desired ester 3, ammonium chloride (colorless crystal, which was detected after the reaction), and the amidate complex A.

**Scheme 4:**
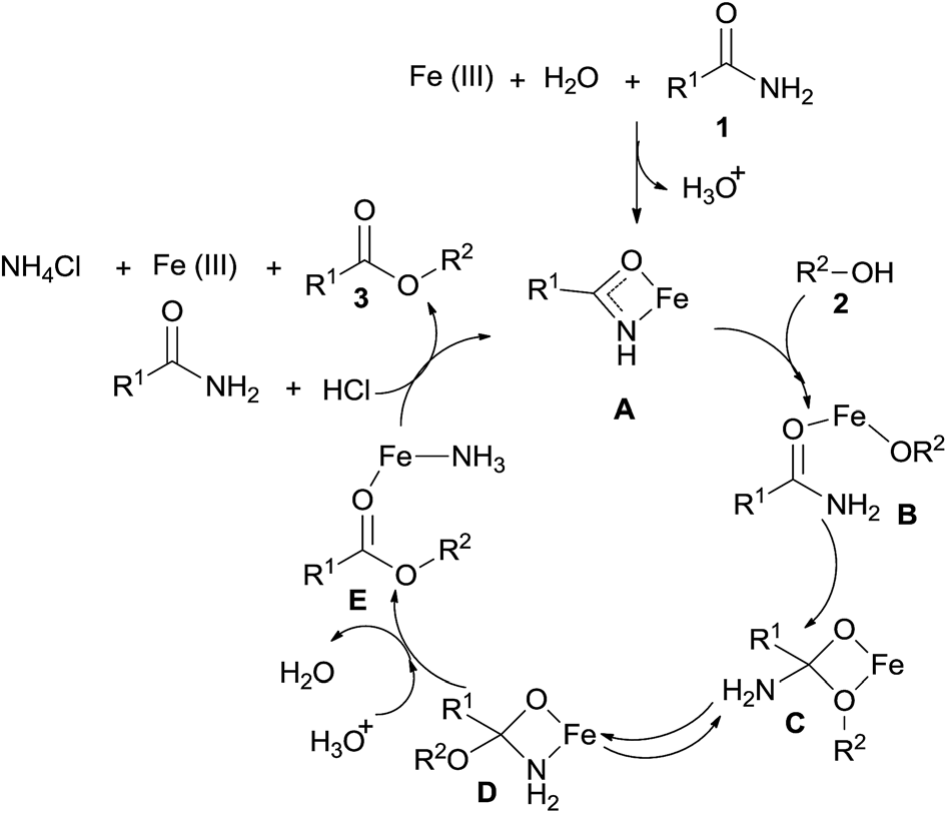
Plausible reaction mechanism for Fe-catalyzed esterification of amides by alcohols.

## Conclusions

In summary, we have discovered a simple, useful and general method for the esterification of amides using inexpensive and readily available iron salts as catalysts. The reaction shows high substrate tolerance as a wide range of aromatic or aliphatic primary, secondary and tertiary amides can be effectively used to produce corresponding esters in good to excellent yields. In addition to alcohol, esters were also used as substrates for esterification of amides through the acyl–acyl exchange process. The present findings not only provide a general and concise method for Fe-catalyzed amide C–N bond cleavage, but also open an avenue for the preparation of esters.

## Conflicts of interest

There are no conflicts to declare.

## Supplementary Material

RA-008-C7RA12152K-s001
