# Supplementary material for: Fe-catalyzed esterification of amides via C–N bond activation
Source: RSC Adv. 2018 Jan 25;8(9):4571–6. doi: 10.1039/c7ra12152k (PMC9077769; doi:10.1039/c7ra12152k)
Supplement: RA-008-C7RA12152K-s001 [file RA-008-C7RA12152K-s001.pdf]

Electronic Supplementary Material (ESI) for RSC Advances.
This journal is © The Royal Society of Chemistry 2018

*Supporting Information*

# Fe-Catalyzed Esterification of Amides via C-N Bond Activation

Xiuling Chen,* Siyin Hu, Rongxing Chen, Jian Wang, Minghu Wu, Haibin Guo, Shaofa Sun*

Non-power Nuclear Technology Collaborative Innovation Center, School of Nuclear Technology and Chemistry & Biology, Hubei University of Science and Technology, Xianning 437100, China

Fax: (+) 86-715-8266953; E-mail: cxl828800@163.com; sunshaofa@mail.hbust.com.cn.

## A. General information

All manipulations were carried out under air atmosphere unless otherwise specified. The reactions were monitored by GC (7820A, Hubei University of Science and Technology) and GC-MS (QP2010, Hunan University). The $^1H$ NMR and $^{13}C$ NMR spectra were recorded on a Brucker ADVANCE III spectrometer at 400 MHz and 100 MHz, respectively (Hubei University of Science and Technology). Flash column chromatography was performed using silica gel 40-70 μm (200-300 mu). Amides were purchased from Energy Chemical, Alfa Aesar, Aladdin or Maya Reagent, alcohols or esters were purchased from Energy Chemical.

## B. General information

A 25 mL Schlenk-type tube equipped with a magnetic stir bar was charged with $FeCl_3.6H_2O$ (0.04 mmol, 20 mol%), then amide **2** (0.24 mmol), alcohol or ester (0.24 mmol), HCl (0.24 mmol, 36%-38%), *n*-hexane (1.0 mL), was added at room temperature, and then the reaction mixture was stirred at 80 ºC for 14 h. The reaction was monitored by GC. After completion of the reaction, the resulting solution was neutralized with saturated NaCl solution. The product was extracted with EtOAc or $CHCl_3$, dried over anhydrous $Na_2SO_4$ and concentrated in vacuo. The crude product was purified by flash column chromatography on silica gel to give analytically pure product.

## C. $^1H$ NMR and $^{13}C$ NMR data of products

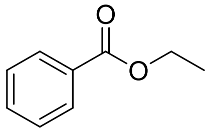

Ethyl benzoate (**3a**)[1]: eluent: petroleum ether; colorless oil; yiled: 85%. $^1H$ NMR (400 MHz, $CDCl_3$) δ: 8.05 (d, $J$ = 8.0 Hz, 2H), 7.54 (t, $J$ = 7.4 Hz, 1H), 7.43 (t, $J$ = 7.6 Hz, 2H), 4.37 (q, $J$ = 7.2 Hz, 2H), 1.39 (t, $J$ = 7.2 Hz, 3H); $^{13}C$ NMR (100 MHz, $CDCl_3$) δ: 166.6, 132.8, 130.5, 129.5, 128.3, 60.9, 14.3; GC-MS: m/z = 150.

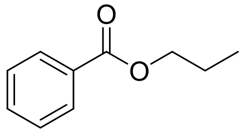

Propyl benzoate (**3b**)[1]: eluent: petroleum ether; colorless oil; yield: 88%. $^1H$ NMR (400 MHz, $CDCl_3$) δ: 8.05 (d, $J$ = 7.2 Hz, 2H), 7.55 (t, $J$ = 7.4 Hz, 1H), 7.44 (t, $J$ = 7.2 Hz, 2H),

4.28 (t, $J$ = 6.6 Hz, 2H), 1.75-1.84 (m, 2H), 1.03 (t, $J$ = 7.4 Hz, 3H); $^{13}$C NMR (100 MHz, $CDCl_3$) δ: 166.7, 132.8, 130.5, 129.5, 128.3, 66.5, 22.1, 10.5; GC-MS: m/z =164.

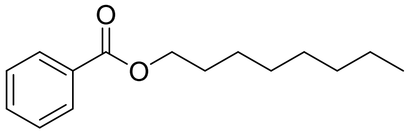

Octyl benzoate (**3c**)[1]: eluent: petroleum ether; colorless oil; yield: 82%. $^1$H NMR (400 MHz, $CDCl_3$) δ: 8.05 (d, $J$ = 7.2 Hz, 2H), 7.55 (t, $J$ = 7.4 Hz, 1H), 7.44 (t, $J$ = 7.0 Hz, 2H), 4.32 (t, $J$ = 6.8 Hz, 2H), 1.73-1.80 (m, 2H), 1.41-1.46 (m, 2H), 1.28-1.34 (m, 8H), 0.89 (t, $J$ = 7.2 Hz, 3H),; $^{13}$C NMR (100 MHz, $CDCl_3$) δ: 166.7, 132.8, 130.5, 129.5, 128.3, 65.1, 31.8, 29.23, 29.17, 28.7, 26.0, 22.6, 14.1; GC-MS: m/z =234.

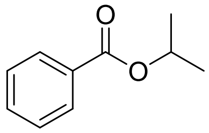

Isopropyl benzoate (**3d**)[2]: eluent: petroleum ether; colorless oil; yield: 91%. $^1$H NMR (400 MHz, $CDCl_3$) δ: 8.04 (d, $J$ = 7.6 Hz, 2H), 7.53 (t, $J$ = 7.4 Hz, 1H), 7.42 (t, $J$ = 7.8 Hz, 2H), 5.21-5.30 (m, 1H), 1.36 (d, $J$ = 6.4 Hz, 6H); $^{13}$C NMR (100 MHz, $CDCl_3$) δ: 166.1, 132.7, 130.9, 129.5, 128.3, 68.3, 21.9; GC-MS: m/z =164.

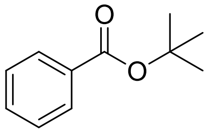

*tert*-Butyl benzoate (**3e**)[3]: eluent: petroleum ether; colorless oil; yield: 81%. $^1$H NMR (400 MHz, $CDCl_3$) δ: 8.00 (d, $J$ = 8.8 Hz, 2H), 7.52 (t, $J$ = 6.6 Hz, 1H), 7.42 (t, $J$ = 7.0 Hz, 2H), 1.60 (s, 9H); $^{13}$C NMR (100 MHz, $CDCl_3$) δ: 165.7, 132.4, 132.0, 129.3, 128.1, 80.9, 28.1; GC-MS: m/z =178.

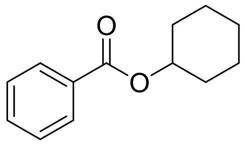

Cyclohexyl benzoate (**3f**)[3]: eluent: petroleum ether; colorless oil; yield: 80%. $^1$H NMR (400 MHz, $CDCl_3$) δ: 8.04 (d, $J$ = 7.2 Hz, 2H), 7.51 (t, $J$ = 7.4 Hz, 1H), 7.40 (t, $J$ = 7.8 Hz, 2H), 5.00-5.06 (m, 1H), 1.92-1.94 (m, 2H), 1.76-1.79 (m, 2H), 1.54-1.59 (m, 3H), 1.34-1.44 (m, 3H); $^{13}$C NMR (100 MHz, $CDCl_3$) δ: 165.9, 132.6, 131.0, 129.5, 128.2, 72.9, 31.6, 25.5, 23.7; GC-MS: m/z =204.

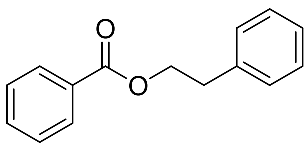

Phenethyl benzoate (**3g**)[4]: eluent: petroleum ether/ethylacetate (200: 1); colorless oil; yield: 83%. $^1$H NMR (400 MHz, $CDCl_3$) δ: 8.01 (d, $J$ = 7.6 Hz, 2H), 7.54 (t, $J$ = 7.4 Hz, 1H), 7.42 (t, $J$ = 7.8 Hz, 2H), 7.22-7.34 (m, 5H), 4.53 (t, $J$ = 7.0 Hz, 2H), 3.08 (t, $J$ = 7.0 Hz, 2H); $^{13}$C NMR (100 MHz, $CDCl_3$) δ: 166.6, 137.9, 132.9, 130.3, 129.6, 129.0, 128.6, 128.4, 126.6, 65.5, 35.3; GC-MS: m/z =226.

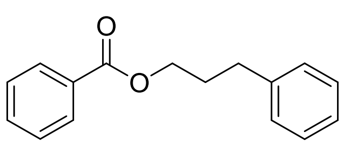

3-Phenylpropyl benzoate (**3h**)[4]: eluent: petroleum ether; colorless oil;

yield: 81%. $^1$H NMR (400 MHz, $CDCl_3$) δ: 8.03 (d, $J$ = 7.6 Hz, 2H), 7.55 (t, $J$ = 7.2 Hz, 1H), 7.43 (t, $J$ = 7.8 Hz, 2H), 7.29 (t, $J$ = 7.4 Hz, 2H), 7.17-7.22 (m, 3H), 4.34 (t, $J$ = 6.6 Hz, 2H), 2.79 (t, $J$ = 7.6 Hz, 2H), 2.07-2.14 (m, 2H); $^{13}$C NMR (100 MHz, $CDCl_3$) δ: 166.6, 141.2, 132.9, 130.4, 129.6, 128.52, 128.49, 128.39, 126.1, 64.3, 32.4, 30.3; GC-MS: m/z =240.

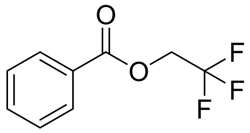


2,2,2-Trifluoroethyl benzoate (**3i**)[5]: eluent: petroleum ether/ethylacetate (30: 1); yield: 81%. $^1$H NMR (400 MHz, $CDCl_3$) δ: 8.07 (t, $J$ = 6.2 Hz, 2H), 7.56-7.63 (m, 1H), 7.43-7.49 (m, 2H), 4.71 (q, $J$ = 8.4 Hz, 2H); $^{13}$C NMR (100 MHz, $CDCl_3$) δ: 164.9, 133.9 (d, $J_{F\text{-}C}$ = 62 Hz), 130.0, 129.8 (q, $J_{F\text{-}C}$ = 69 Hz), 128.6, 127.3 (q, $J_{F\text{-}C}$ = 275 Hz), 61.3 (q, $J_{F\text{-}C}$ = 37 Hz); GC-MS: m/z =204.

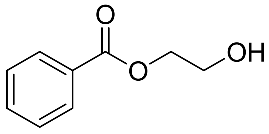


2-Hydroxyethyl benzoate (**3j**)[6]: eluent: petroleum ether/ethylacetate (10: 1); colorless oil; yield: 85%. $^1$H NMR (400 MHz, $CDCl_3$) δ: 8.07 (d, $J$ = 8.0 Hz, 2H), 7.57 (t, $J$ = 7.4 Hz, 1H), 7.45 (t, $J$ = 7.6 Hz, 2H), 4.57 (t, $J$ = 5.6 Hz, 2H), 3.81 (t, $J$ = 5.6 Hz, 2H); $^{13}$C NMR (100 MHz, $CDCl_3$) δ: 166.2, 133.3, 129.8, 129.6, 128.5, 64.5, 41.7; GC-MS: m/z =166.

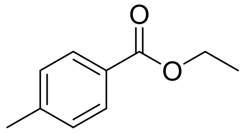


Ethyl 4-methylbenzoate (**3k**)[7]: eluent: petroleum ether/ethylacetate (50:1); Colorless oil; yield: 86%. $^1$H NMR (400 MHz, $CDCl_3$) δ: 7.94 (d, $J$ = 8.0 Hz, 2H), 7.23 (d, $J$ = 8.0 Hz, 2H), 4.37 (q, $J$ = 7.2 Hz, 2H), 2.40 (s, 3H), 1.38 (t, $J$ = 7.2 Hz, 3H); $^{13}$C NMR (100 MHz, $CDCl_3$) δ: 166.7, 143.4, 129.6, 129.0, 127.8, 60.8, 21.6, 14.4; GC-MS: m/z =164.

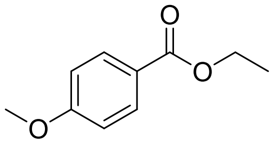


Ethyl 4-methoxybenzoate (**3l**)[7]: eluent: petroleum ether/ethylacetate (50:1); colorless oil; yield: 85%. $^1$H NMR (400 MHz, $CDCl_3$) δ: 7.98 (d, $J$ = 8.4 Hz, 2H), 6.89 (d, $J$ = 8.0 Hz, 2H), 4.34 (q, $J$ = 7.0 Hz, 2H), 3.84 (s, 3H), 1.37 (t, $J$ = 7.0 Hz, 3H); $^{13}$C NMR (100 MHz, $CDCl_3$) δ: 166.4, 163.3, 131.5, 123.0, 113.5, 60.6, 55.4, 14.4; GC-MS: m/z =180..

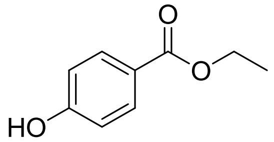


Ethyl 4-hydroxybenzoate (**3m**)[7]: eluent: petroleum ether/ethylacetate (10:1); colorless oil; yield: 78%. $^1$H NMR (400 MHz, $CDCl_3$) δ: 7.96 (d, $J$ = 8.4 Hz, 2H), 7.40 (s, 1H), 6.91 (d, $J$ = 8.4 Hz, 2H), 4.37 (q, $J$ = 7.2 Hz, 2H), 1.38 (t, $J$ = 7.2 Hz, 3H); $^{13}$C NMR (100 MHz, $CDCl_3$) δ: 167.5, 160.8, 132.0, 122.1, 115.4, 61.2, 14.3; GC-MS: m/z =166.

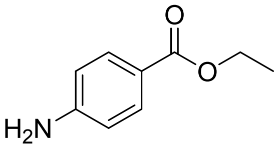

Ethyl 4-aminobenzoate (**3n**)[7]: eluent: petroleum ether/ethylacetate (10:1); colorless oil; yield: 75%. $^1$H NMR (400 MHz, $CDCl_3$) δ: 7.86 (d, *J* = 8.0 Hz, 2H), 6.63 (d, *J* = 8.4 Hz, 2H), 4.32 (q, *J* = 7.0 Hz, 2H), 4.09 (s, 2H), 1.35 (t, *J* = 7.2 Hz, 3H); $^{13}$C NMR (100 MHz, $CDCl_3$) δ: 166.8, 151.0, 131.6, 119.9, 113.8, 60.4, 14.4; GC-MS: m/z =165.

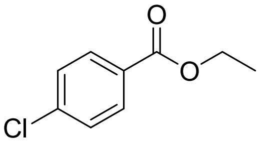

Ethyl 4-chlorobenzoate (**3o**)[7]: eluent: petroleum ether/ethylacetate (100:1); colorless oil; yield: 86%. $^1$H NMR (400 MHz, $CDCl_3$) δ: 7.97 (d, *J* = 8.8 Hz, 2H), 7.40 (d, *J* = 8.4 Hz, 2H), 4.38 (q, *J* = 7.2 Hz, 2H), 1.39 (t, *J* = 7.2 Hz, 3H); $^{13}$C NMR (100 MHz, $CDCl_3$) δ: 165.7, 139.2, 130.9, 128.9, 128.6, 61.2, 14.3; GC-MS: m/z =184.

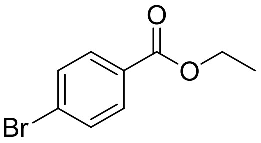

Ethyl 4-bromobenzoate (**3p**)7: eluent: petroleum ether/ethylacetate (100:1); colorless oil, yield: 84%. $^1$H NMR (400 MHz, $CDCl_3$) δ: 7.89 (d, *J* = 8.4 Hz, 2H), 7.56 (d, *J* = 8.4 Hz, 2H), 4.37 (q, *J* = 7.2 Hz, 2H), 1.38 (t, *J* = 7.2 Hz, 3H); $^{13}$C NMR (100 MHz, $CDCl_3$) δ: 165.9, 131.7, 131.1, 129.4, 127.9, 61.2, 14.3; GC-MS: m/z =227.

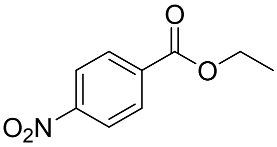

Ethyl 4-nitrobenzoate (**3q**)7: eluent: petroleum ether; mp: 56.3-57.1°C, white solid; yield: 90%. $^1$H NMR (400 MHz, $CDCl_3$) δ: 8.25 (d, *J* = 8.4 Hz, 2H),8.18 (d, *J* = 8.8 Hz, 2H), 4.42 (q, *J* = 7.2 Hz, 2H),1.40 (t, *J* = 7.2 Hz, 3H); $^{13}$C NMR (100 MHz, $CDCl_3$) δ: 164.7, 150.5, 135.8, 130.6, 123.5, 61.9, 14.2; GC-MS: m/z =195.

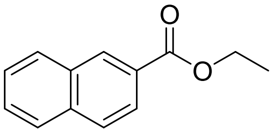

Ethyl 2-naphthoate (**3r**)[8]: eluent: petroleum ether; colorless oil; yield: 86%. $^1$H NMR (400 MHz, $CDCl_3$) δ: 8.62 (s, 1H), 8.08 (d, *J* = 8.8 Hz, 1H), 7.95 (d, *J* = 8.0 Hz, 1H), 7.87 (d, *J* = 8.4 Hz, 2H), 7.52-7.60 (m, 2H), 4.46 (q, *J* = 7.2 Hz, 2H), 1.46 (t, *J* = 7.2 Hz, 3H); $^{13}$C NMR (100 MHz, $CDCl_3$) δ: 166.8, 135.5, 132.5, 131.0, 129.4, 128.17, 128.10, 127.8, 126.6, 125.2, 61.1, 14.4; GC-MS: m/z =200.

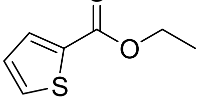

Ethyl thiophene-2-carboxylate (**3s**)[9]: eluent: petroleum ether; colorless oil; yield: 84%. $^1$H NMR (400 MHz, $CDCl_3$) δ: 7.79 (d, *J* = 4.0 Hz, 1H), 7.53 (d, *J* = 4.8 Hz, 1H), 7.09 (t, *J* = 44 Hz, 1H), 4.36 (q, *J* = 7.0 Hz, 2H), 1.37 (t, *J* = 7.2 Hz, 3H), $^{13}$C NMR (100 MHz, $CDCl_3$) δ: 162.3, 134.1, 133.3, 132.2, 127.7, 61.1, 14.3; GC-MS: m/z =156.

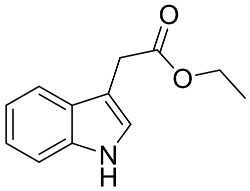

Ethyl 2-(1H-indol-3-yl)acetate (**3t**)[13]: eluent: petroleum ether; colorless oil; yield: 55%. $^1$H NMR (400 MHz, $CDCl_3$) δ: 8.14 (s, 1H), 7.60 (d, *J* = 8.0 Hz, 1H), 7.23 (d, *J* = 8.0 Hz, 1H), 7.09-7.20 (m, 2H), 6.98 (d, *J* = 2.0 Hz, 1H), 4.14 (q, *J* = 7.0 Hz, 2H), 3.74 (s, 1H), 1.23 (t, *J* = 7.0 Hz, 3H); $^{13}$C NMR (100 MHz, $CDCl_3$) δ: 172.3, 136.2, 127.3, 123.3, 122.1, 119.6, 118.9, 111.4, 108.3, 60.9, 31.5, 14.3; GC-MS: m/z =203.

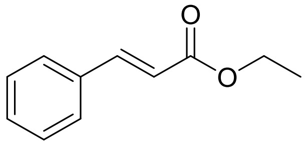

Ethyl cinnamate (**3u**)[14]: eluent: petroleum ether; colorless oil; yield: 84%. $^1$H NMR (400 MHz, $CDCl_3$) δ: 7.70 (d, *J* = 16.0 Hz, 1H), 7.52-7.54 (m, 2H), 7.38-7.39 (m, 3H), 6.43 (d, *J* = 16.0 Hz, 1H), 4.26 (q, *J* = 7.0 Hz, 2H), 1.34 (t, *J* = 7.0 Hz, 3H), $^{13}$C NMR (100 MHz, $CDCl_3$) δ: 167.0, 144.6, 134.5, 130..2, 128.9, 128.1, 118.3, 60.5, 14.3; GC-MS: m/z =176.

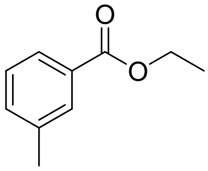

Ethyl 3-methylbenzoate (**3v**)[10]: eluent: petroleum ether; colorless oil; yield: 85%. $^1$H NMR (400 MHz, $CDCl_3$) δ: 7.86 (s, 1H), 7.83 (s, 1H), 7.30-7.35 (m, 2H), 4.38 (q, *J* = 7.0 Hz, 2H), 2.40 (s, 3H), 1.39 (t, *J* = 7.0 Hz, 3H); $^{13}$C NMR (100 MHz, $CDCl_3$) δ: 166.8, 138.1, 133.6, 130.4, 130.1, 128.2, 126.7, 60.9, 21.3, 14.4; GC-MS: m/z =164.

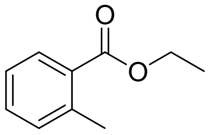

Ethyl 2-methylbenzoate (**3w**)[10]: eluent: petroleum ether; colorless oil; yield: 81%. $^1$H NMR (400 MHz, $CDCl_3$) δ: 7.90 (d, *J* = 8.4 Hz, 1H), 7.38 (t, *J* = 7.4 Hz, 1H), 7.22-7.25 (m, 2H), 4.36 (q, *J* = 7.2 Hz, 2H), 2.60 (s, 3H), 1.39 (t, *J* = 7.2 Hz, 3H); $^{13}$C NMR (100 MHz, $CDCl_3$) δ: 167.7, 140.0, 131.8, 131.6, 130.5, 130.0, 125.7, 60.7, 21.7, 14.3; GC-MS: m/z =164.

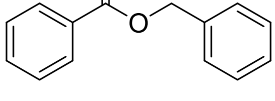

Benzyl benzoate (**3x**)[4]: eluent: petroleum ether; colorless oil; yield: 78%. $^1$H NMR (400 MHz, $CDCl_3$) δ: 8.08 (d, *J* = 8.0 Hz, 2H), 7.56 (t, *J* = 7.2 Hz, 1H), 7.33-7.46 (m, 7H), 5.37 (s, 2H); $^{13}$C NMR (100 MHz, $CDCl_3$) δ: 166.4, 136.1, 133.0, 130.1, 129.7, 128.6, 128.4, 128.22,128.15, 66.7; GC-MS: m/z =212.

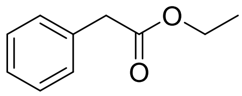

Ehyl 2-phenylacetate (**3y**)[11]: eluent: petroleum ether; colorless oil; yield: 86%. $^1$H NMR (400 MHz, $CDCl_3$) δ: 7.24-7.34 (m, 5H), 4.15 (q, *J* = 7.0 Hz, 2H), 3.60 (s, 3H), 1.24 (t, *J* =

7.2 Hz, 3H); $^{13}C$ NMR (100 MHz, $CDCl_3$) δ: 171.6, 134.2, 129.3, 128.6, 127.1, 60.9, 41.5, 14.2; GC-MS: m/z =164.

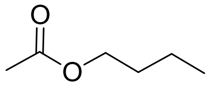
Butyl acetate (**3z**)[12]: eluent: petroleum ether; colorless oil; yield: 79%. $^1H$ NMR (400 MHz, $CDCl_3$) δ: 4.03 (t, *J* = 6.6 Hz, 2H), 2.00 (s, 3H), 1.54-1.61 (m, 2H), 1.30-1.39 (m, 2H), 0.90 (t, *J* = 7.4 Hz, 3H); $^{13}C$ NMR (100 MHz, $CDCl_3$) δ: 171.1, 64.3, 30.6, 20.9, 19.1, 13.6. GC-MS: m/z =116.

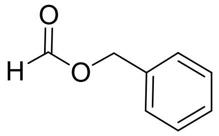
Benzyl formate (**3z$^1$**)[12]: eluent: petroleum ether; colorless oil; yield: 82%. $^1H$ NMR (400 MHz, $CDCl_3$) δ: 8.15 (s, 1H), 7.35-7.39 (m, 5H), 5.21 (s, *2*H); $^{13}C$ NMR (100 MHz, $CDCl_3$) δ: 160.8, 135.3, 128.7, 128.5, 128.4, 65.7; GC-MS: m/z =136.

## D. References


(1) M. Tamura, S. M. A. H. Siddikib, K. Shimizu, *Green Chem.,* **2013**, *15*, 1641–1646.

(2) S. Mahmood, T. Li, B. Xu, Y. Guo, S. Zhang, *Asian Journal of Organic Chemistry*, **2017**, *6*, 768-774.

(3) L. Hie, N. F. Fine Nathel, T. K. Shah, E. L. Baker, X. Hong, Y. F.Yang, P. Liu; K. N. Houk, N. K. Garg, *Nature*, **2015**, *524*, 79-83.

(4) S. M. A. H. Siddiki, A. S. Touchy, M. Tamura, K. Shimizu, *RSC Adv*. **2014**, *4*, 35803-35807.

(5) H. Rikiya, F. Chika, Y. Ryo, O. Takashi, *Chemistry - A European Journa*l, **2016**, *22*, 12278-12281

(6) H. Sharghi, M. H. Sarvari, *J. Org. Chem*. **2003**, *68*, 4096-4099.

(7) R. V. Jagadeesh, H. Junge, M. Pohl, J. Radnik, A. Bruckner, M. Beller, *J. Am. Chem. Soc*. **2013**, *135*, 10776-10782.

(8) S. T. Heller, T. Fu, R. Sarpong, *Org. Lett.* **2012**, 14, 1970-1973.

(9) Y. Zhou, D. Yang, G. Luo, Y. Zhao, Y. Luo, N. Xue, J. Qu, *Tetrahedron*, **2014**, *70*, 4668-4674.

(10) P. A. Wade, S. A. Rutkowsky, D. B. King, *Journal of Chemical Education*, **2006**, *83*, 927-928.

(11) H., Robin, D. Peter, M. Christina, *Synthesis*, **2016**, *48*, 3175-3182.

(12) J. Cao, B. Qi, J. Liu, Y. Shang, H. Liu, W. Wang, J. Lv, Z. Chen, H. Zhang, X. Zhou, *RSC Adv*, **2016**, *6*, 21612–21616.

(13) S. Faisal, Q. Zang, P. K. Maity, A. Brandhofer, P. C. Kearney, O. Reiser, R. N. Grass, D. Stoianova, P. R. Hanson, *Org. Lett.* **2017**, *19*, 2274−2277.

(14) L Hei, C. Feng, Z. Li, L. Liu, J. Gui, *Youji Huaxue*, **2015**, 35, 1673-1681.

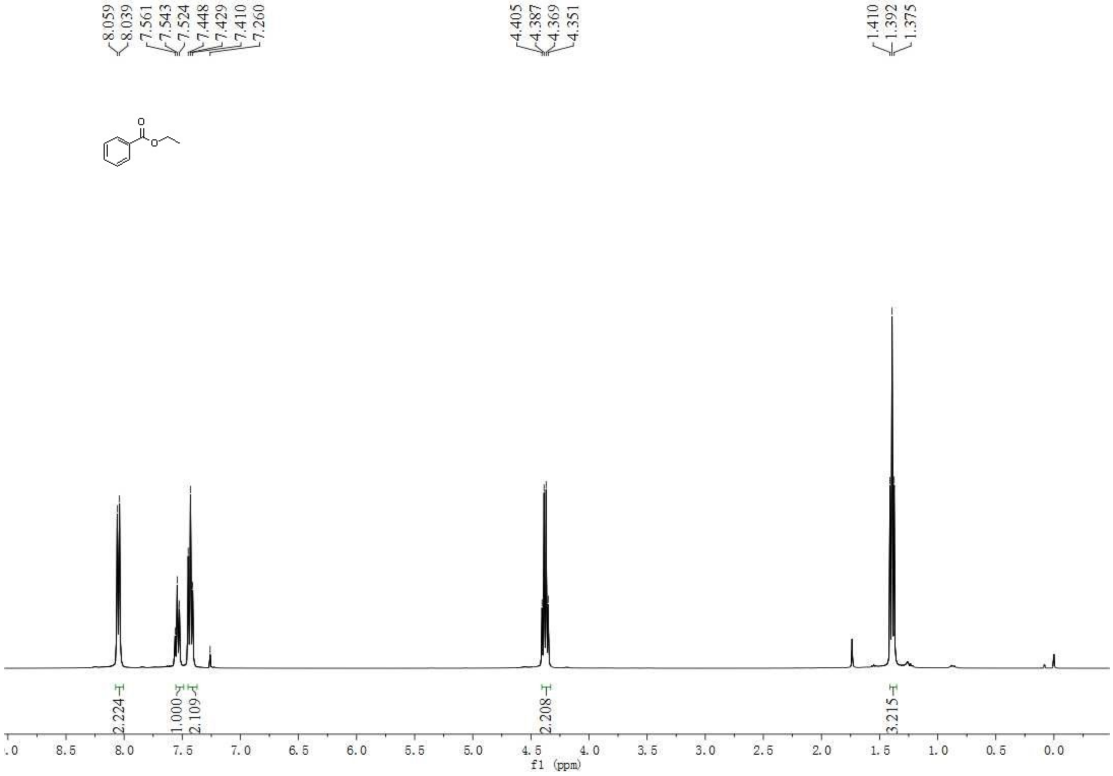

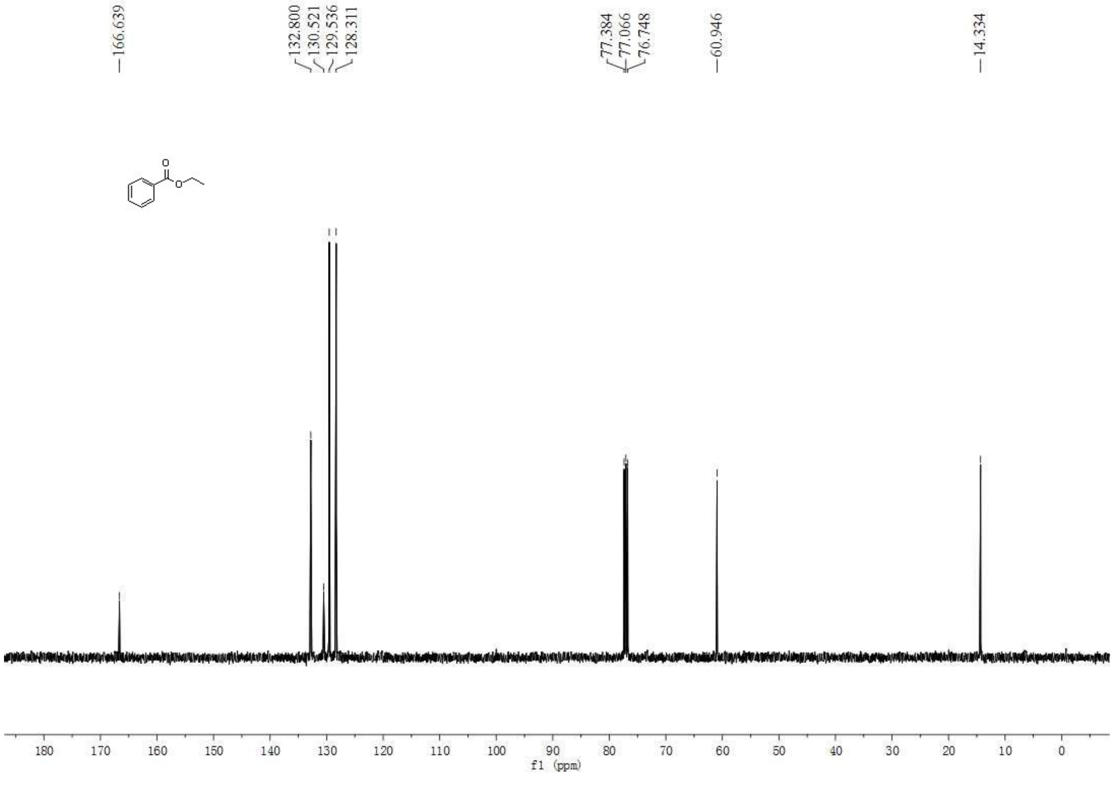

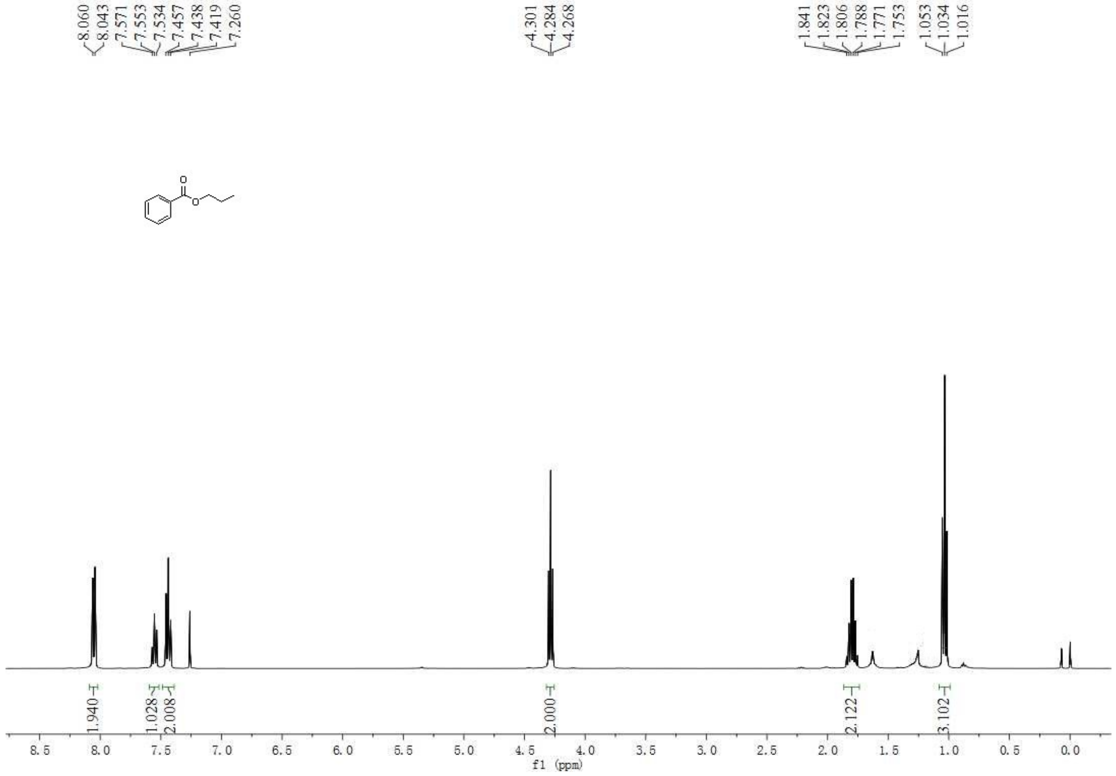
8.060
8.043
7.571
7.553
7.534
7.457
7.438
7.419
7.260
4.301
4.284
4.268
1.841
1.823
1.806
1.788
1.771
1.753
1.053
1.034
1.016
1.940
1.028
2.008
2.000
2.122
3.102
f1 (ppm)

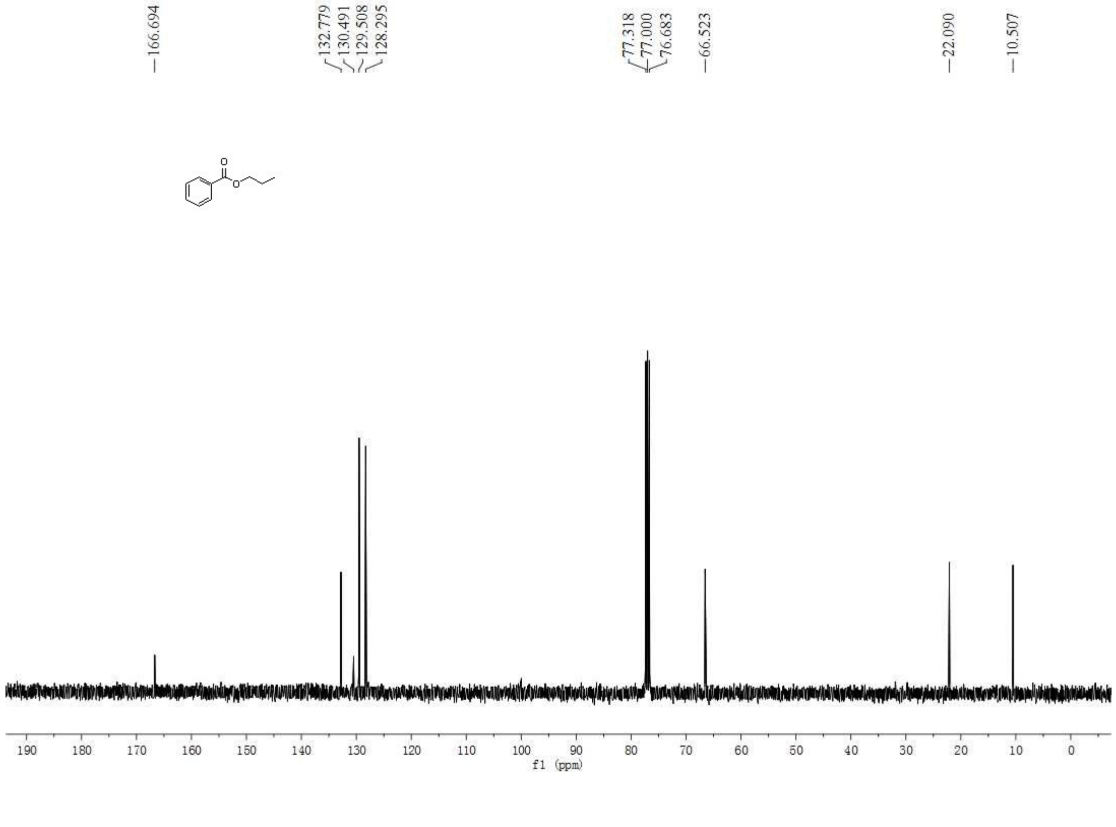
166.694
132.779
130.491
129.508
128.295
77.318
77.000
76.683
66.523
22.090
10.507
f1 (ppm)

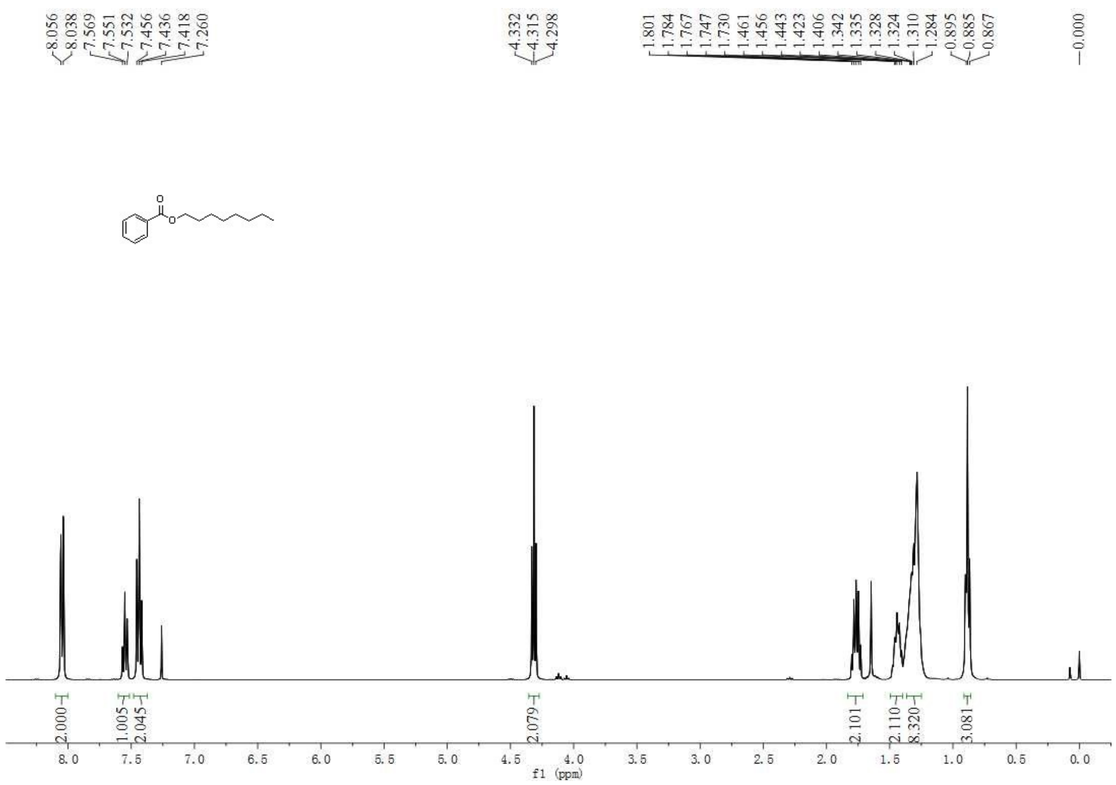
8.056
8.038
7.569
7.551
7.532
7.456
7.436
7.418
7.260
4.332
4.315
4.298
1.801
1.784
1.767
1.747
1.730
1.461
1.456
1.443
1.423
1.406
1.342
1.335
1.328
1.324
1.310
1.284
0.895
0.885
0.867
0.000
2.000
1.005
2.045
2.079
2.101
2.110
8.320
3.081
f1 (ppm)

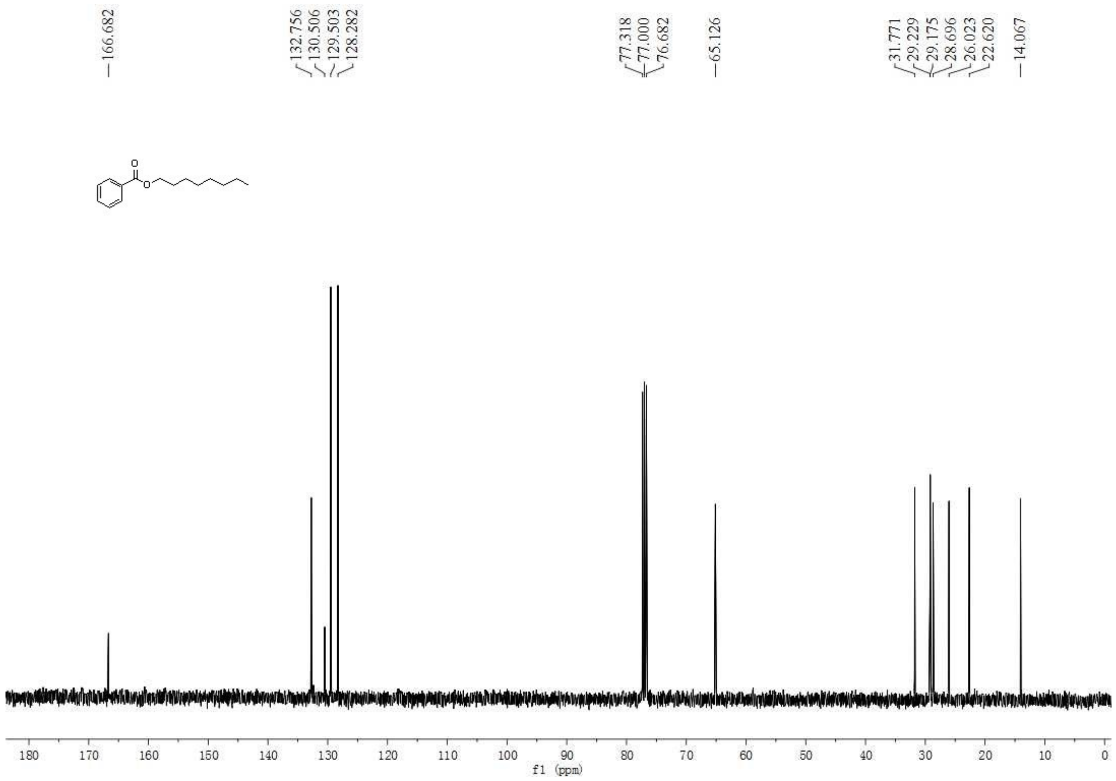
166.682
132.756
130.506
129.503
128.282
77.318
77.000
76.682
65.126
31.771
29.229
29.175
28.696
26.023
22.620
14.067
f1 (ppm)

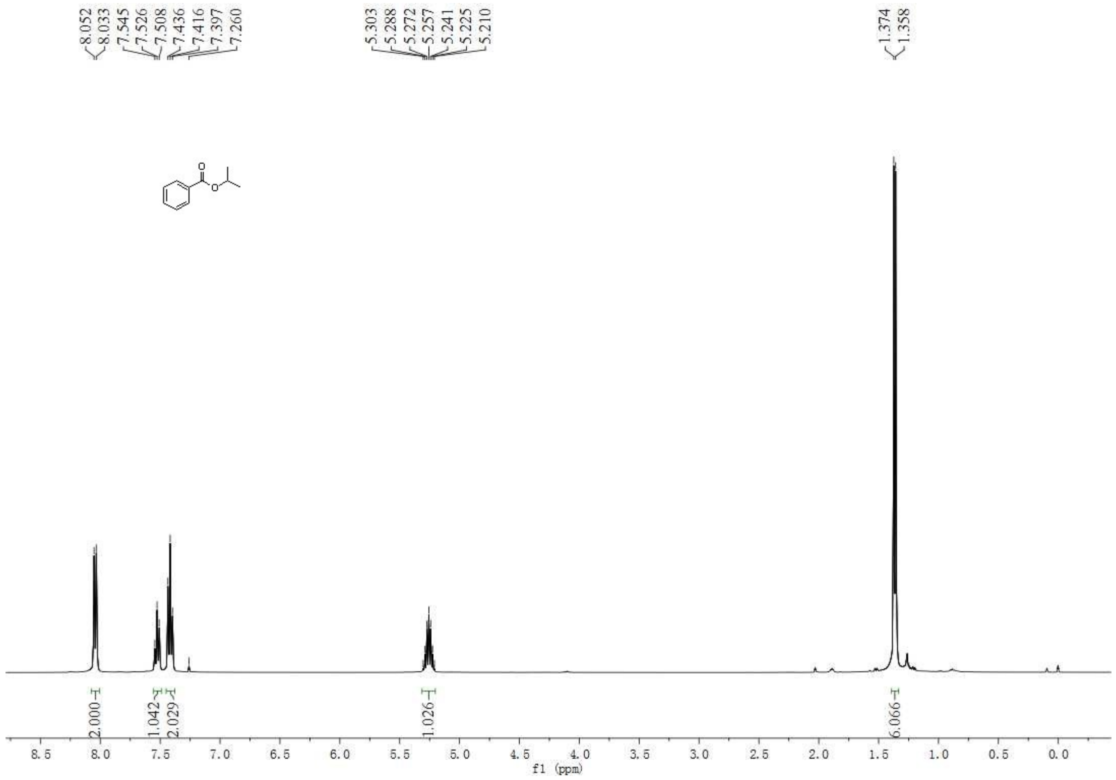
8.052
8.033
7.545
7.526
7.508
7.436
7.416
7.397
7.260
5.303
5.288
5.272
5.257
5.241
5.225
5.210
1.374
1.358
2.000
1.042
2.029
1.026
6.066
f1 (ppm)

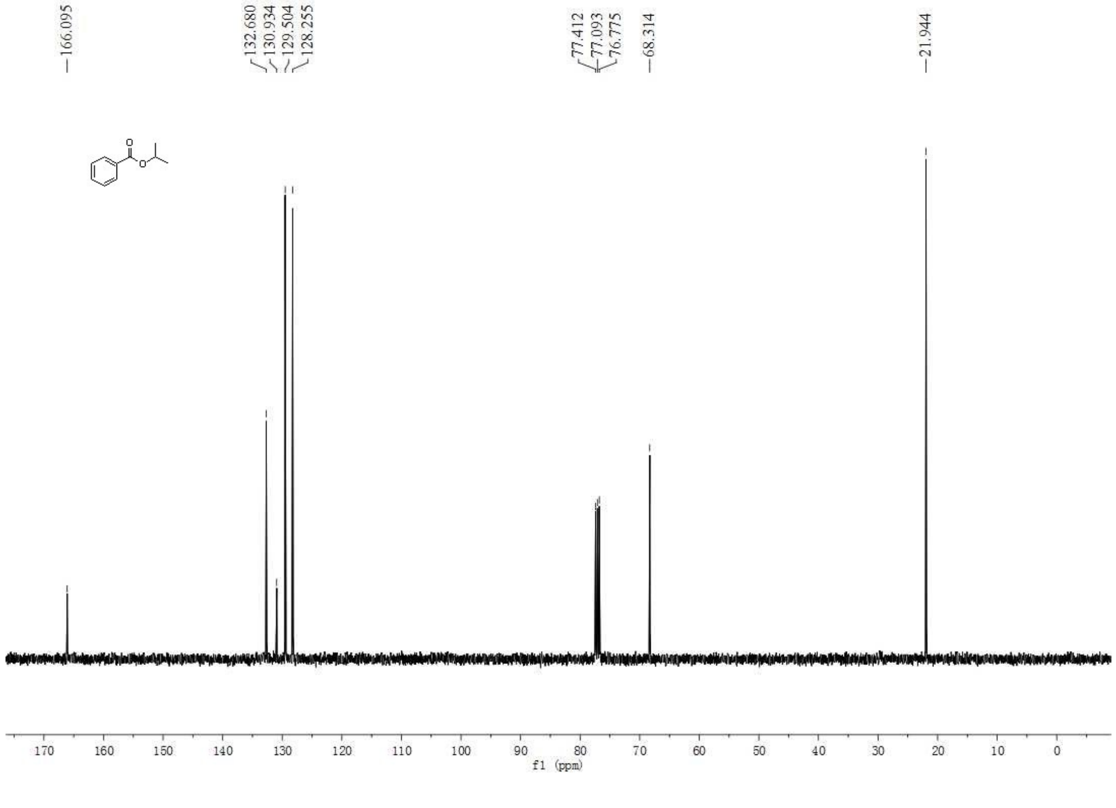
166.095
132.680
130.934
129.504
128.255
77.412
77.093
76.775
68.314
21.944
f1 (ppm)

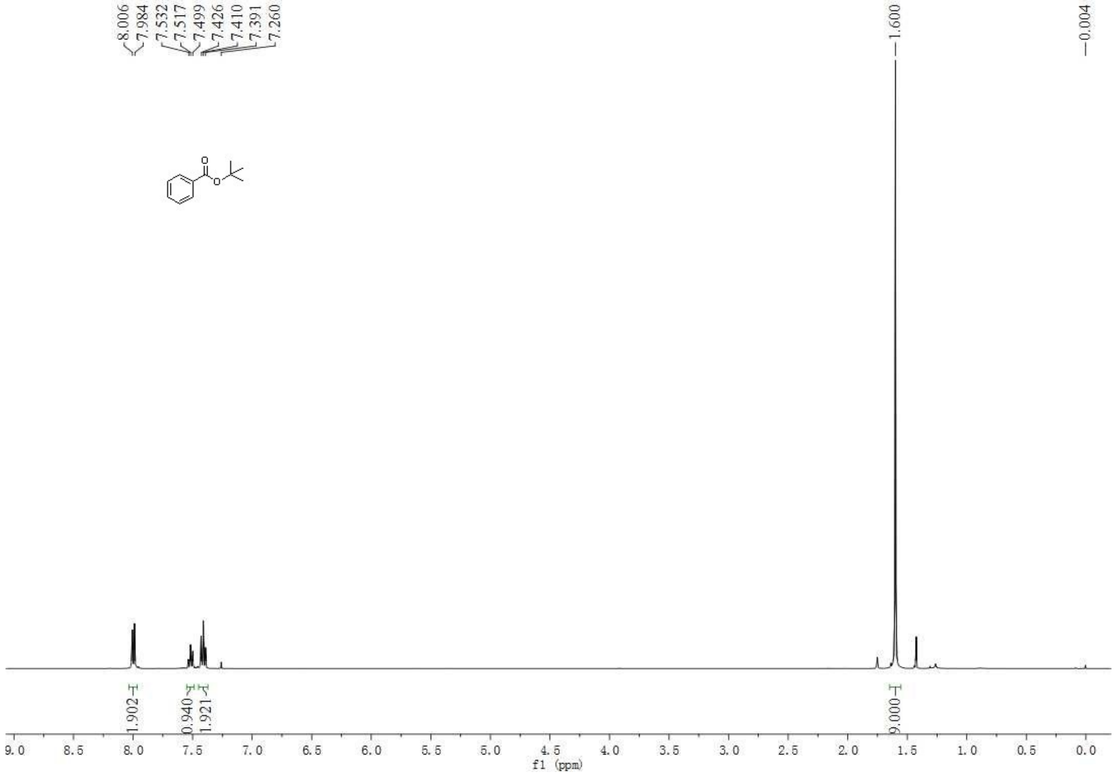
8.006
7.984
7.532
7.517
7.499
7.426
7.410
7.391
7.260
1.600
-0.004
1.902
0.940
1.921
9.000
f1 (ppm)

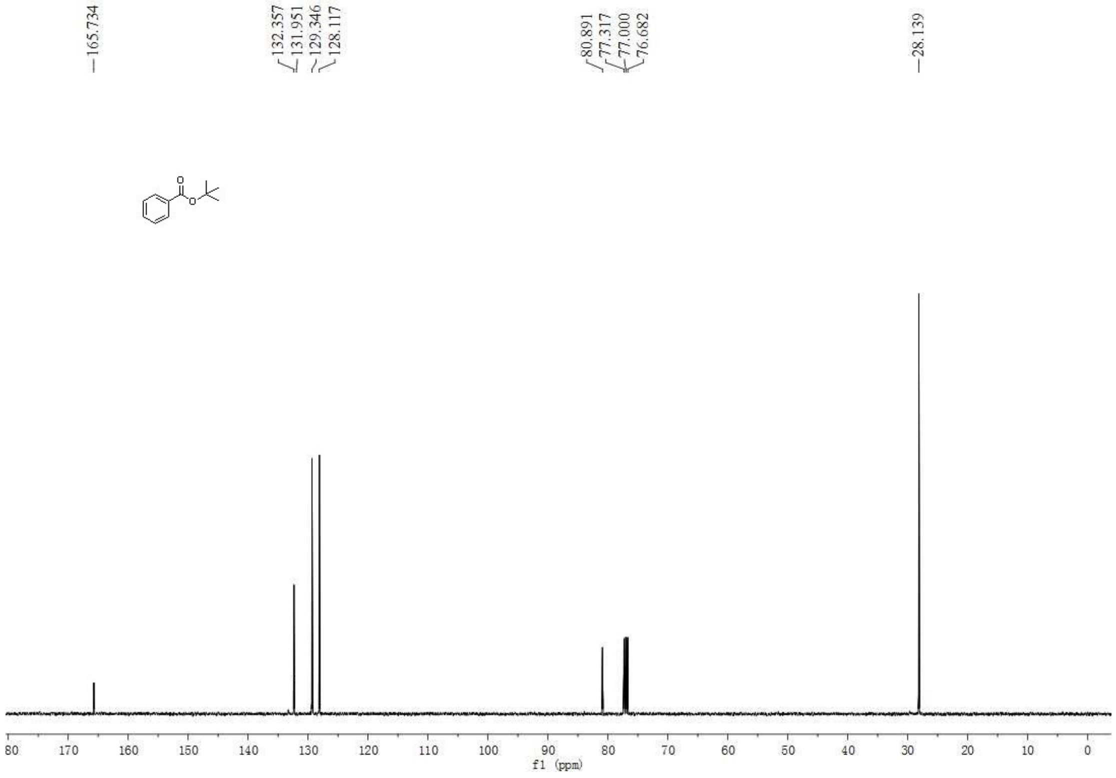
165.734
132.357
131.951
129.346
128.117
80.891
77.317
77.000
76.682
28.139
f1 (ppm)

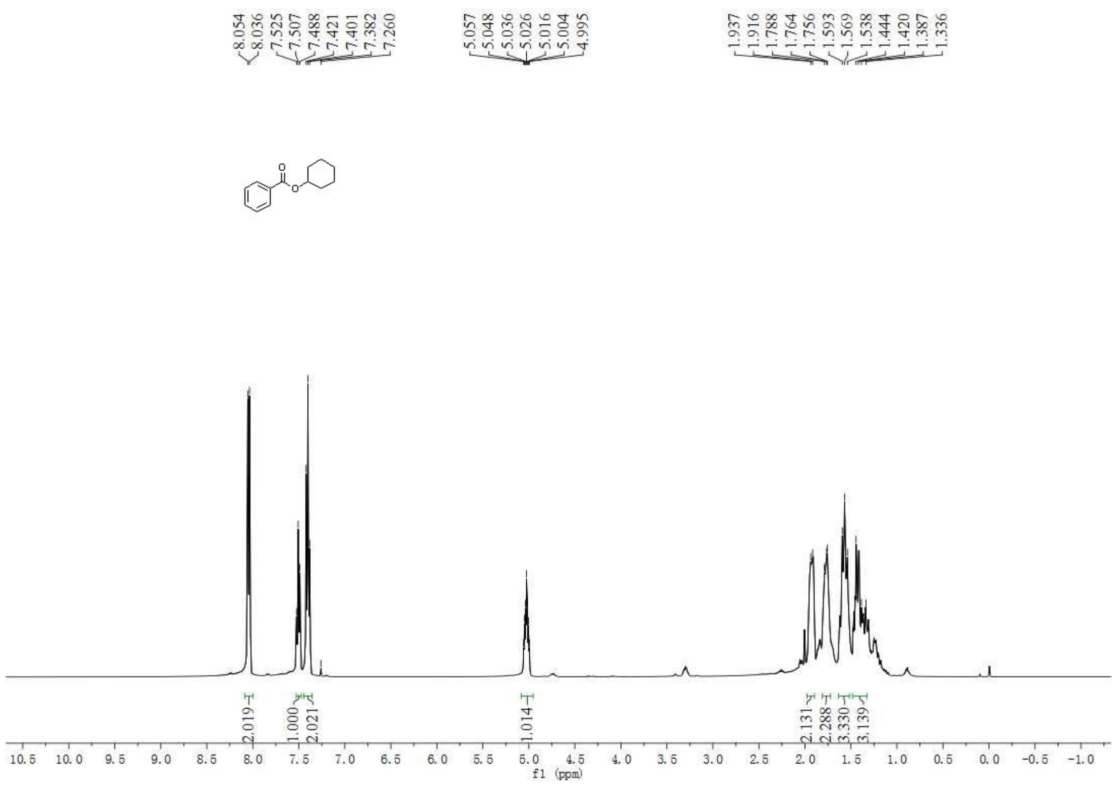
8.054
8.036
7.525
7.507
7.488
7.421
7.401
7.382
7.260
5.057
5.048
5.036
5.026
5.016
5.004
4.995
1.937
1.916
1.788
1.764
1.756
1.593
1.569
1.538
1.444
1.420
1.387
1.336
2.019
1.000
2.021
1.014
2.131
2.288
3.330
3.139
f1 (ppm)

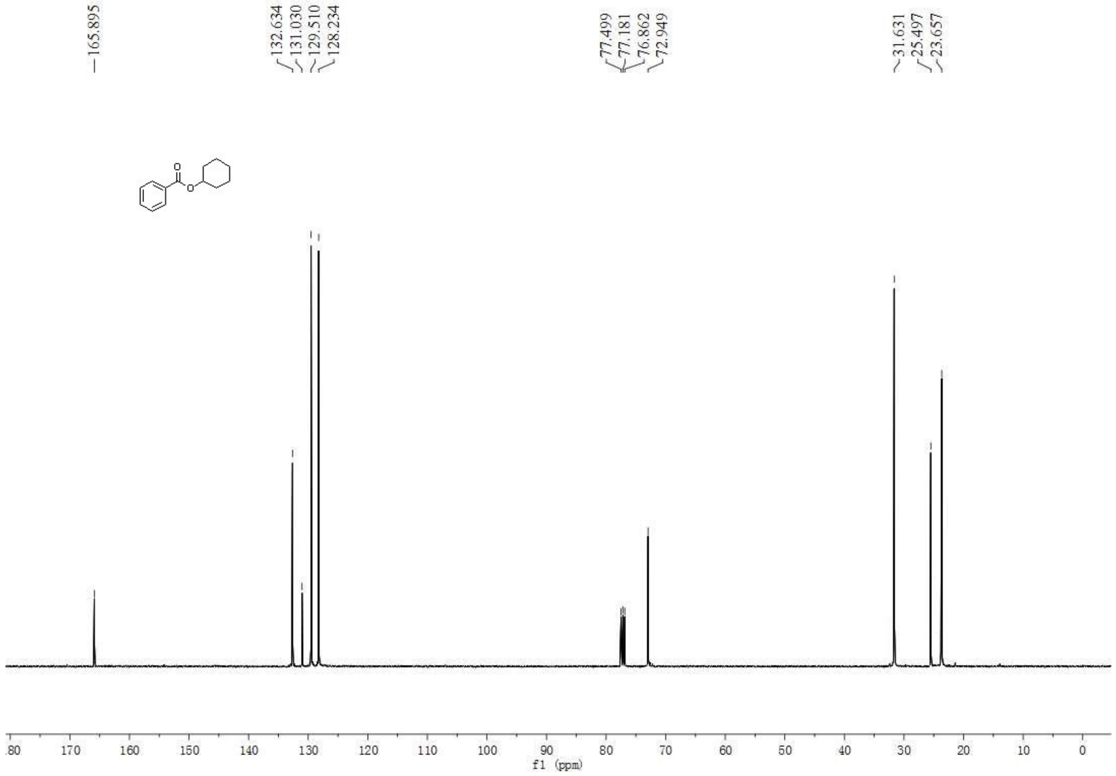
165.895
132.634
131.030
129.510
128.234
77.499
77.181
76.862
72.949
31.631
25.497
23.657
f1 (ppm)

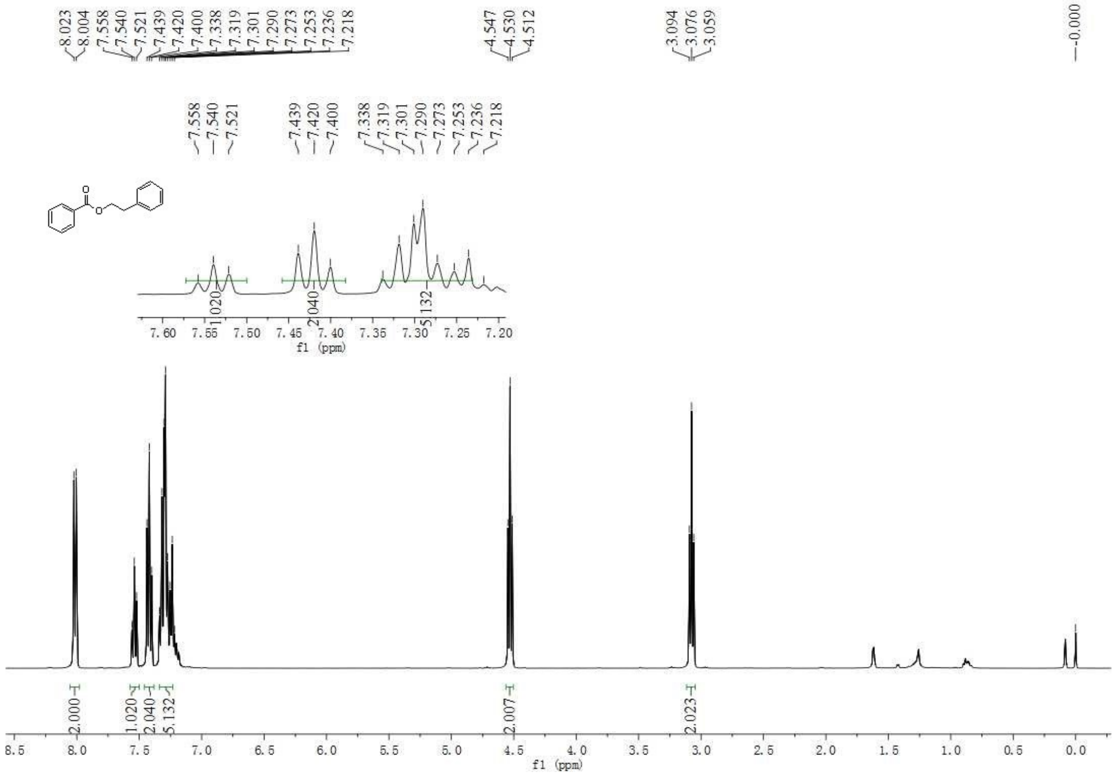
8.023
8.004
7.558
7.540
7.521
7.439
7.420
7.400
7.338
7.319
7.301
7.290
7.273
7.253
7.236
7.218
4.547
4.530
4.512
3.094
3.076
3.059
0.000
1.020
2.040
5.132
f1 (ppm)
2.000
2.007
2.023
f1 (ppm)

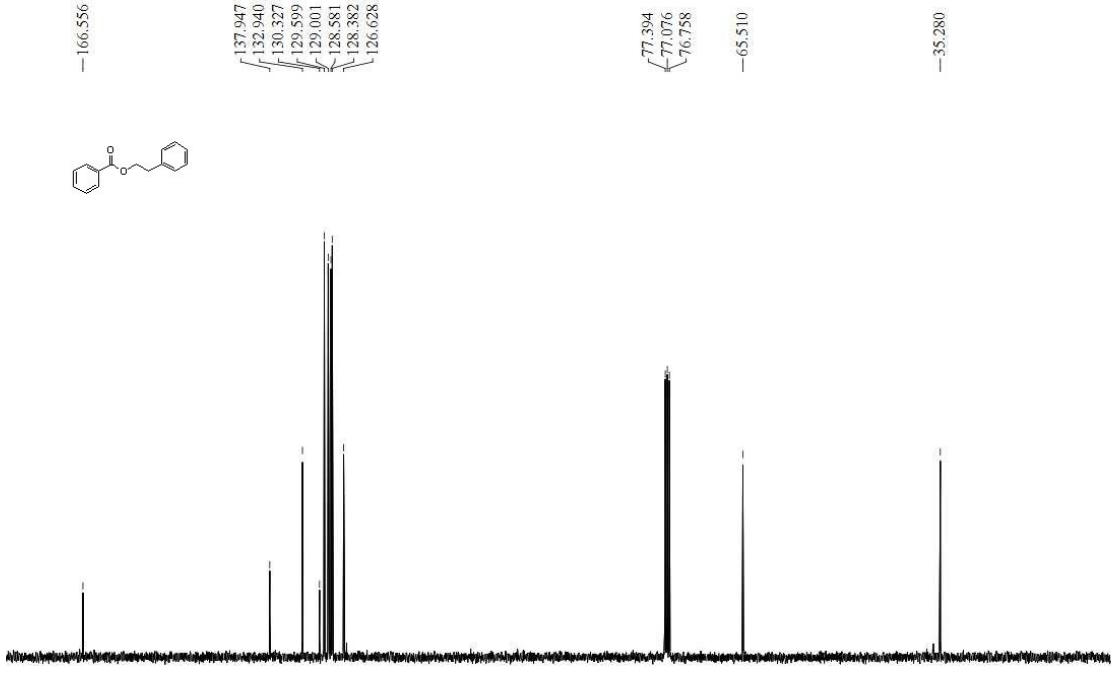
166.556
137.947
132.940
130.327
129.599
129.001
128.581
128.382
126.628
77.394
77.076
76.758
65.510
35.280
f1 (ppm)

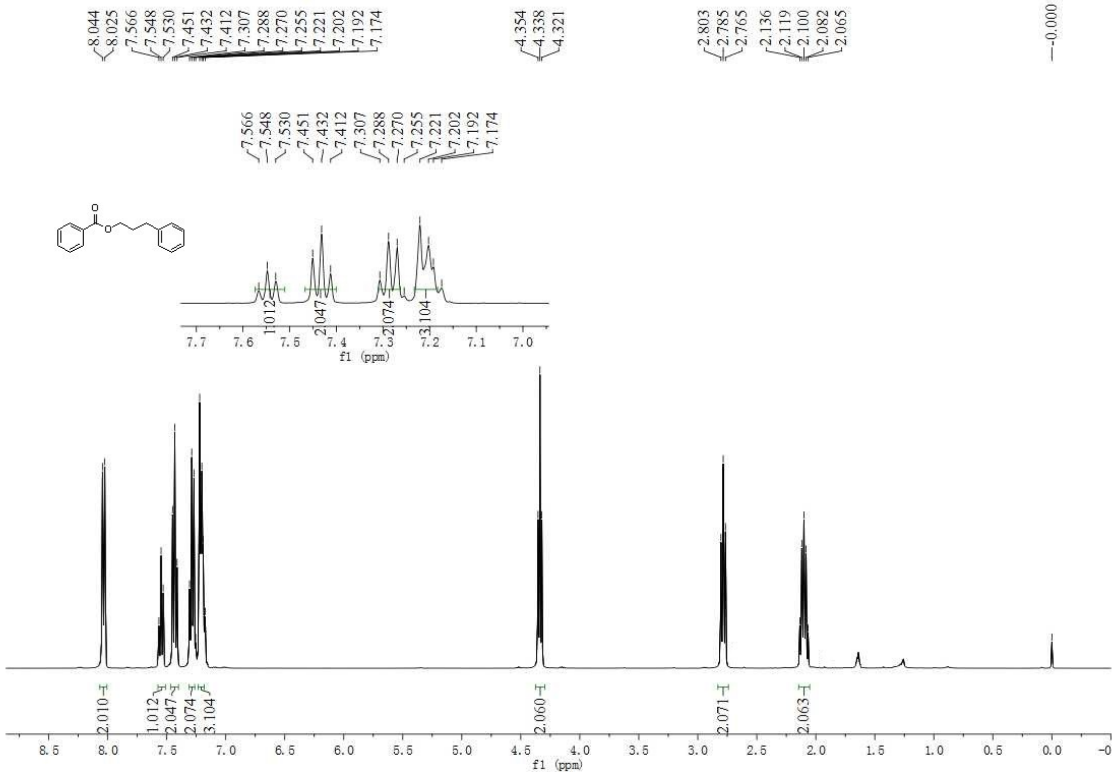
8.044
8.025
7.566
7.548
7.530
7.451
7.432
7.412
7.307
7.288
7.270
7.255
7.221
7.202
7.192
7.174
4.354
4.338
4.321
2.803
2.785
2.765
2.136
2.119
2.100
2.082
2.065
0.000
2.010
1.012
2.047
2.074
3.104
2.060
2.071
2.063
f1 (ppm)

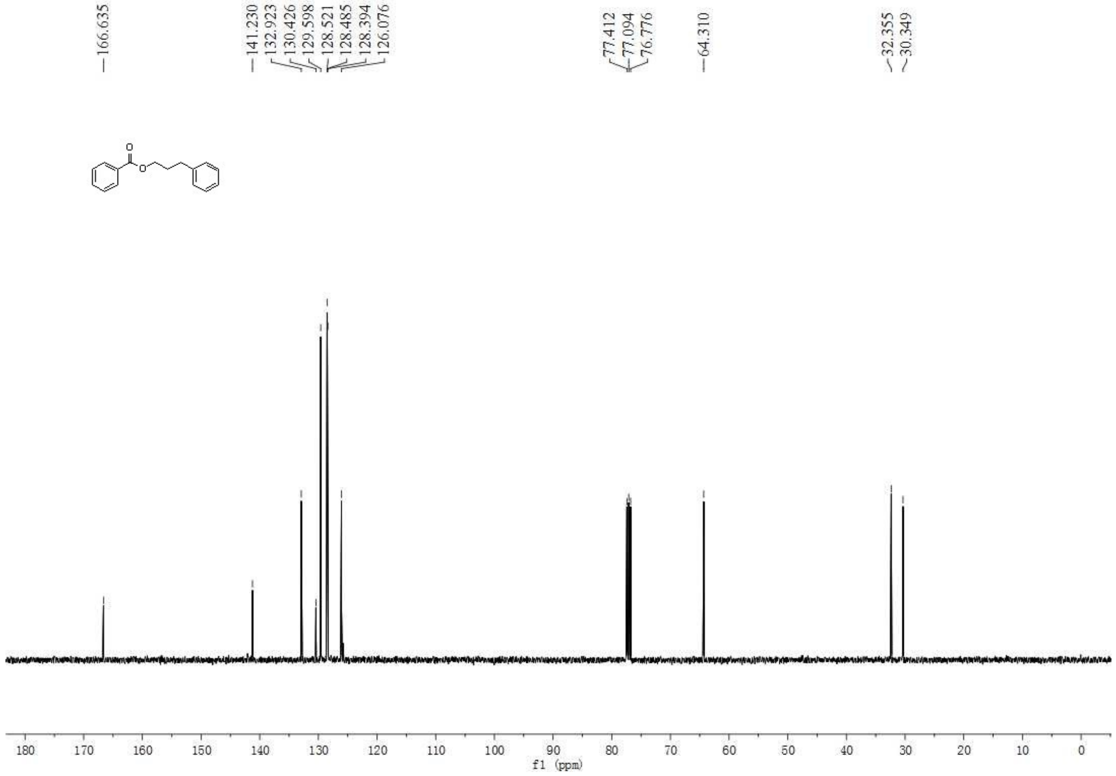
166.635
141.230
132.923
130.426
129.598
128.521
128.485
128.394
126.076
77.412
77.094
76.776
64.310
32.355
30.349
f1 (ppm)

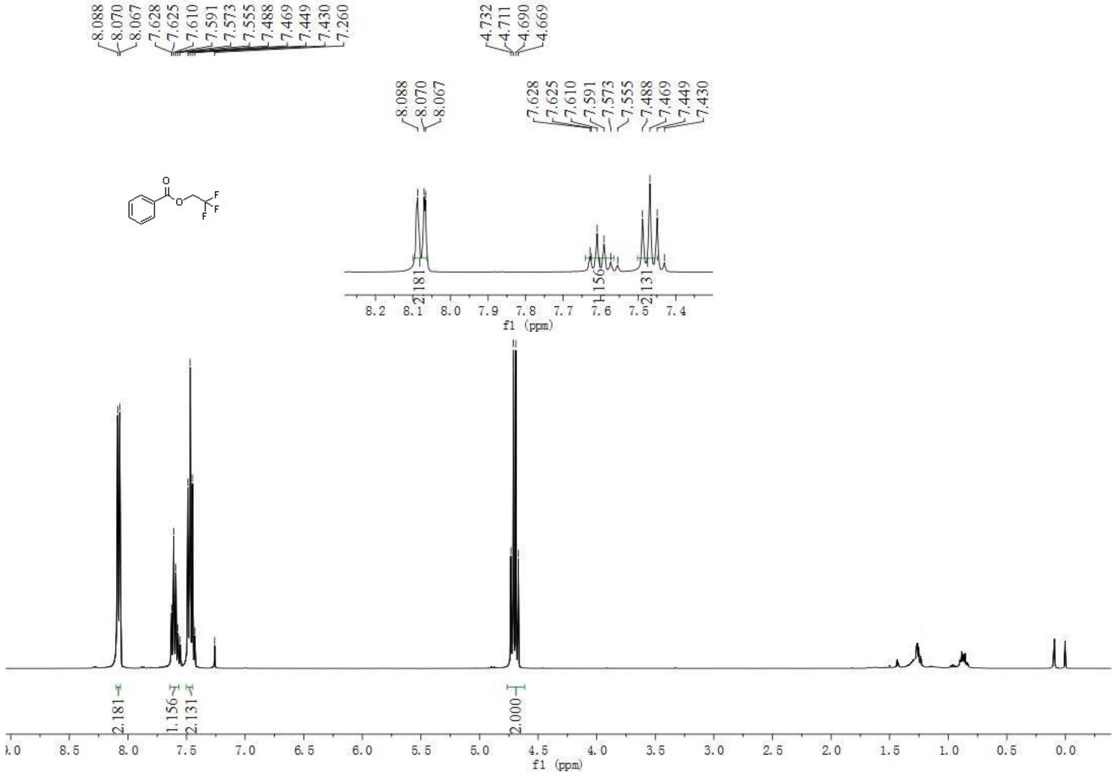

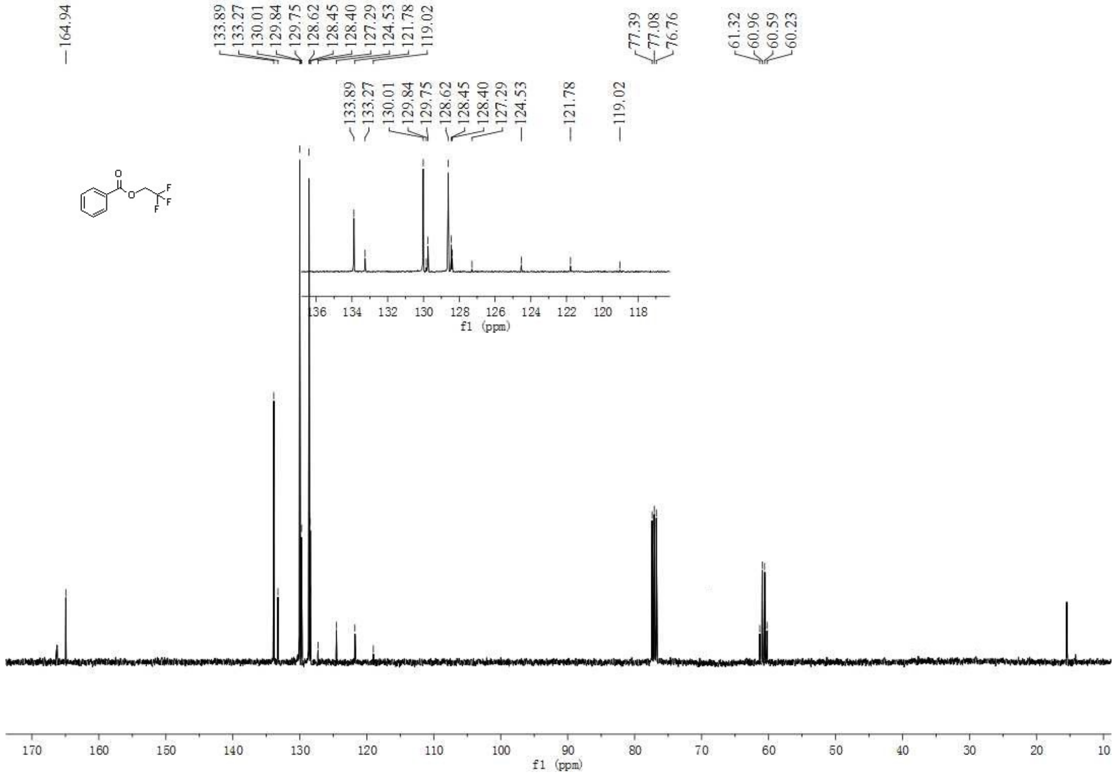

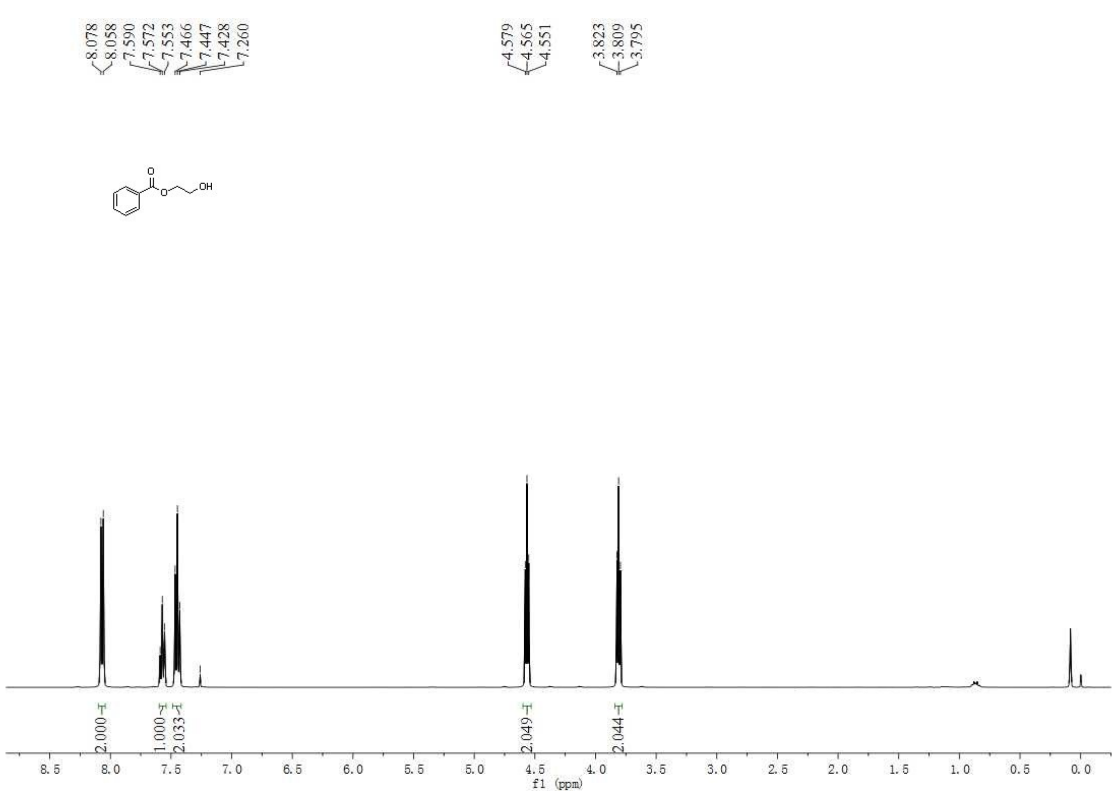
8.078
8.058
7.590
7.572
7.553
7.466
7.447
7.428
7.260
4.579
4.565
4.551
3.823
3.809
3.795
O
O
OH
2.000
1.000
2.033
2.049
2.044
f1 (ppm)

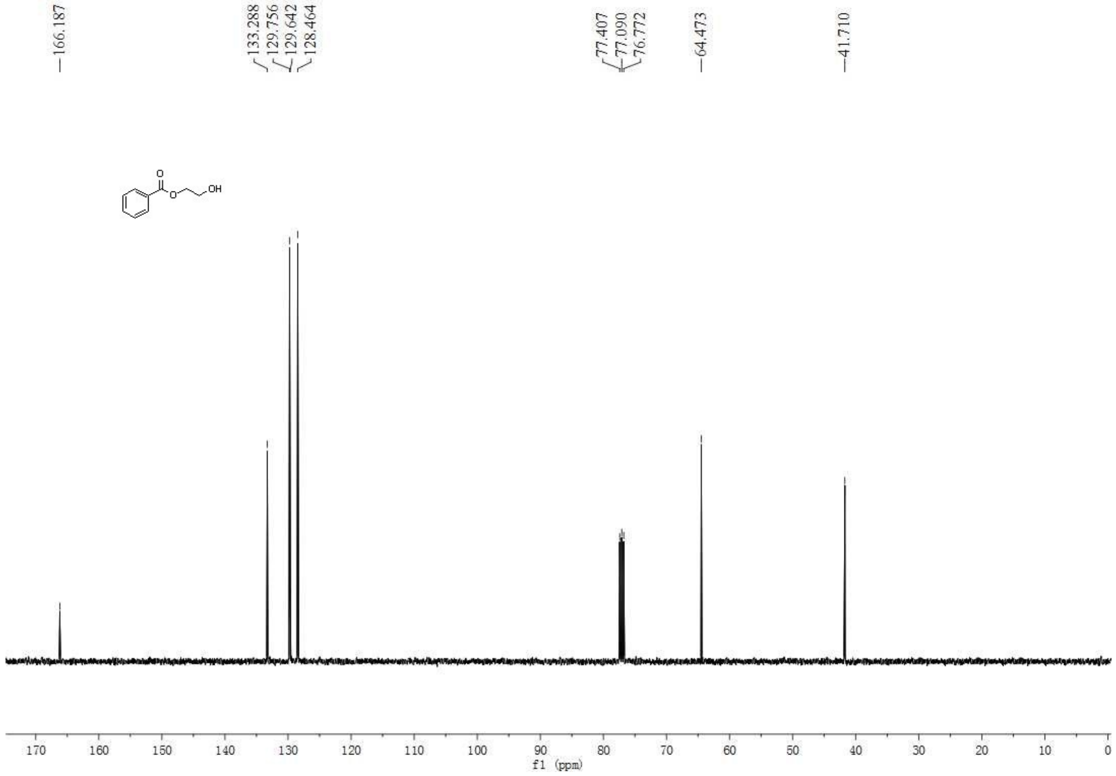
166.187
133.288
129.756
129.642
128.464
77.407
77.090
76.772
64.473
41.710
O
O
OH
f1 (ppm)

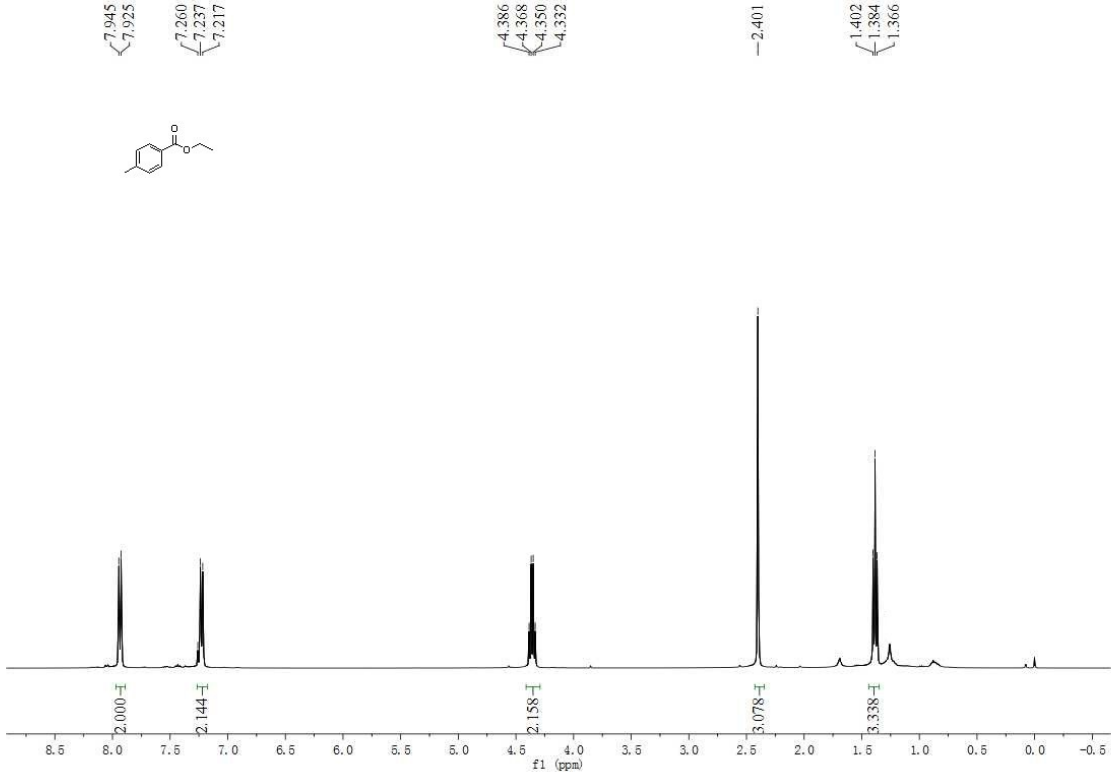
7.945
7.925
7.260
7.237
7.217
4.386
4.368
4.350
4.332
2.401
1.402
1.384
1.366
2.000
2.144
2.158
3.078
3.338
f1 (ppm)

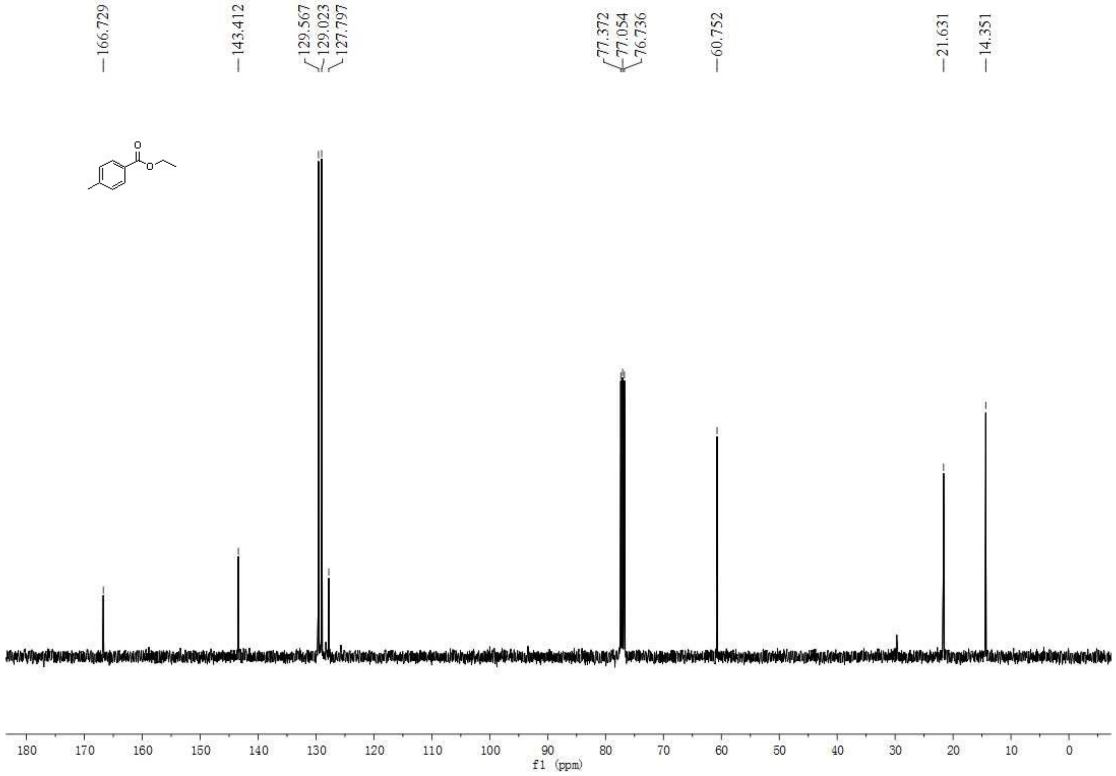
166.729
143.412
129.567
129.023
127.797
77.372
77.054
76.736
60.752
21.631
14.351
f1 (ppm)

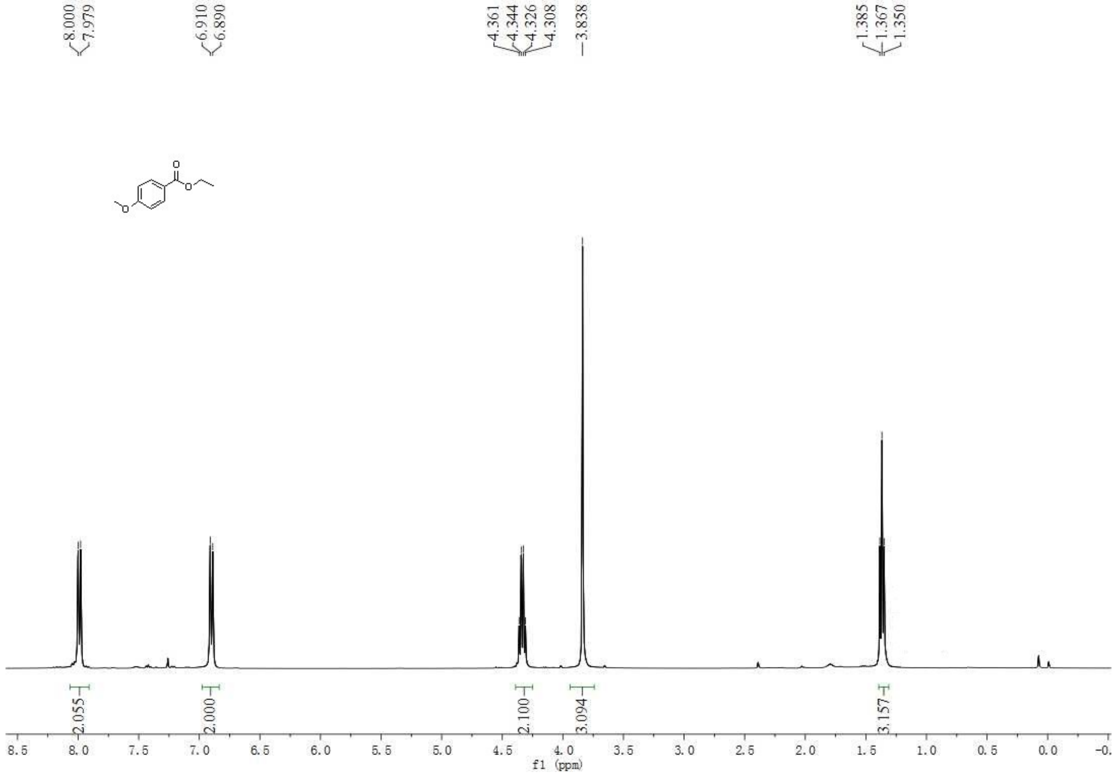
8.000
7.979
6.910
6.890
4.361
4.344
4.326
4.308
3.838
1.385
1.367
1.350
2.055
2.000
2.100
3.094
3.157
f1 (ppm)

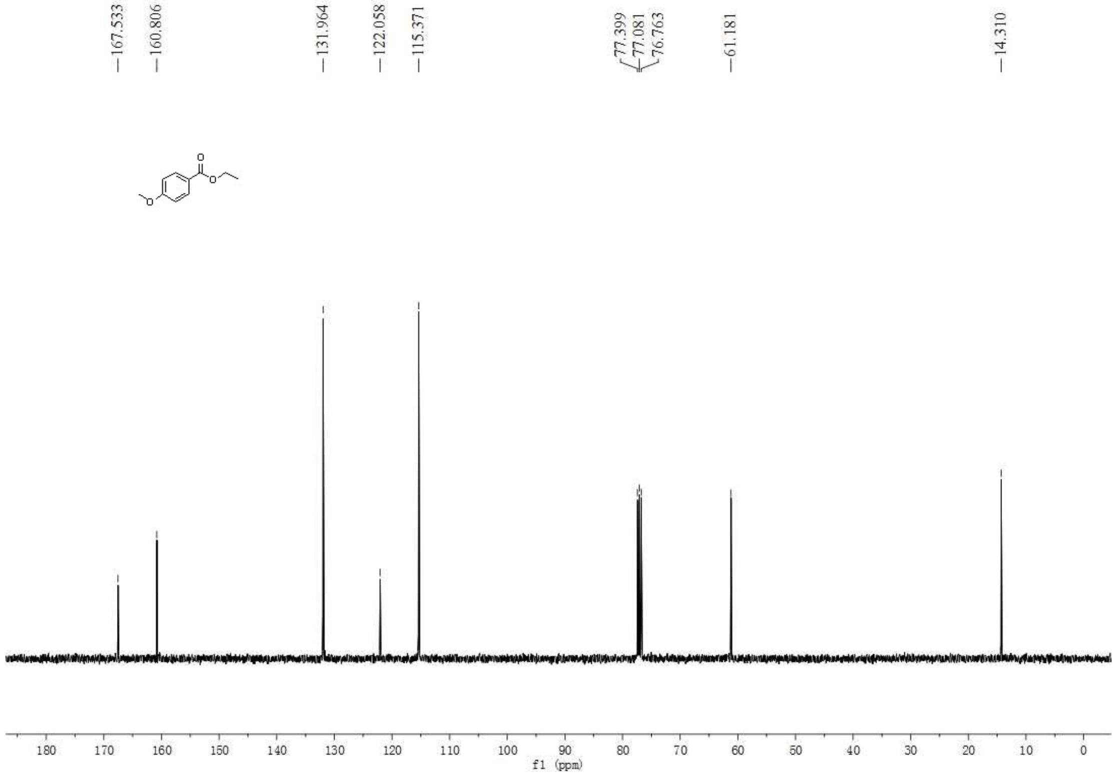
167.533
160.806
131.964
122.058
115.371
77.399
77.081
76.763
61.181
14.310
f1 (ppm)

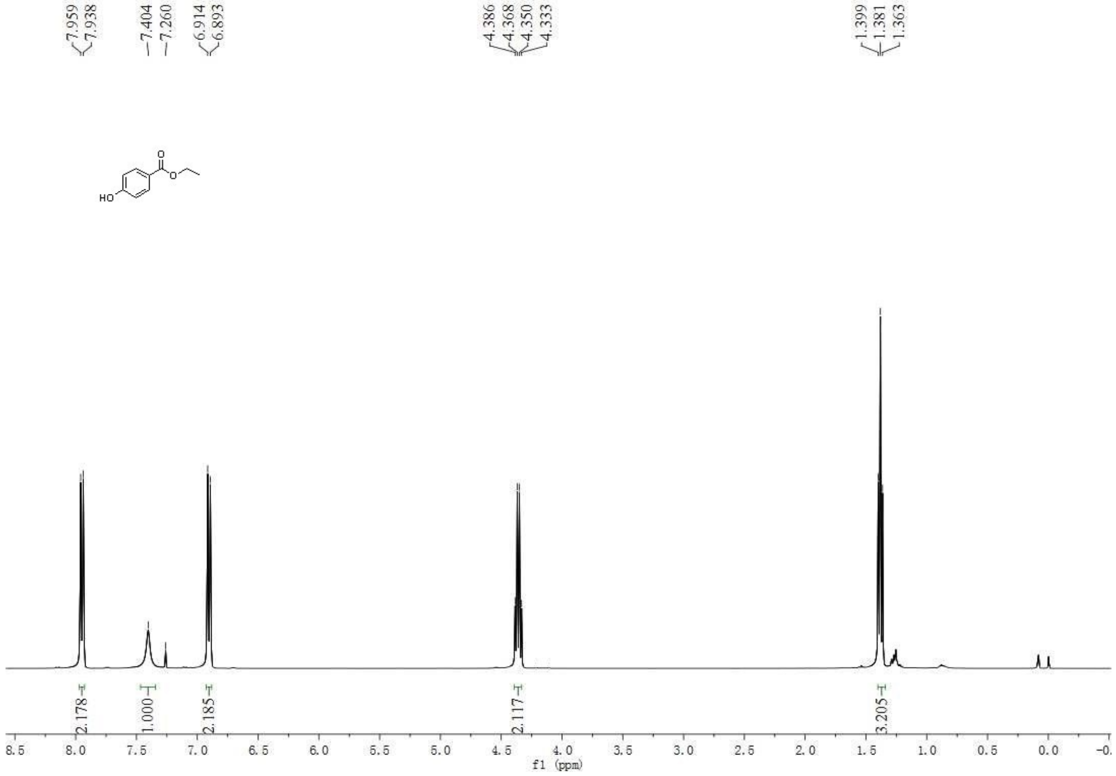
7.959
7.938
7.404
7.260
6.914
6.893
4.386
4.368
4.350
4.333
1.399
1.381
1.363
HO
O
O
2.178
1.000
2.185
2.117
3.205
f1 (ppm)

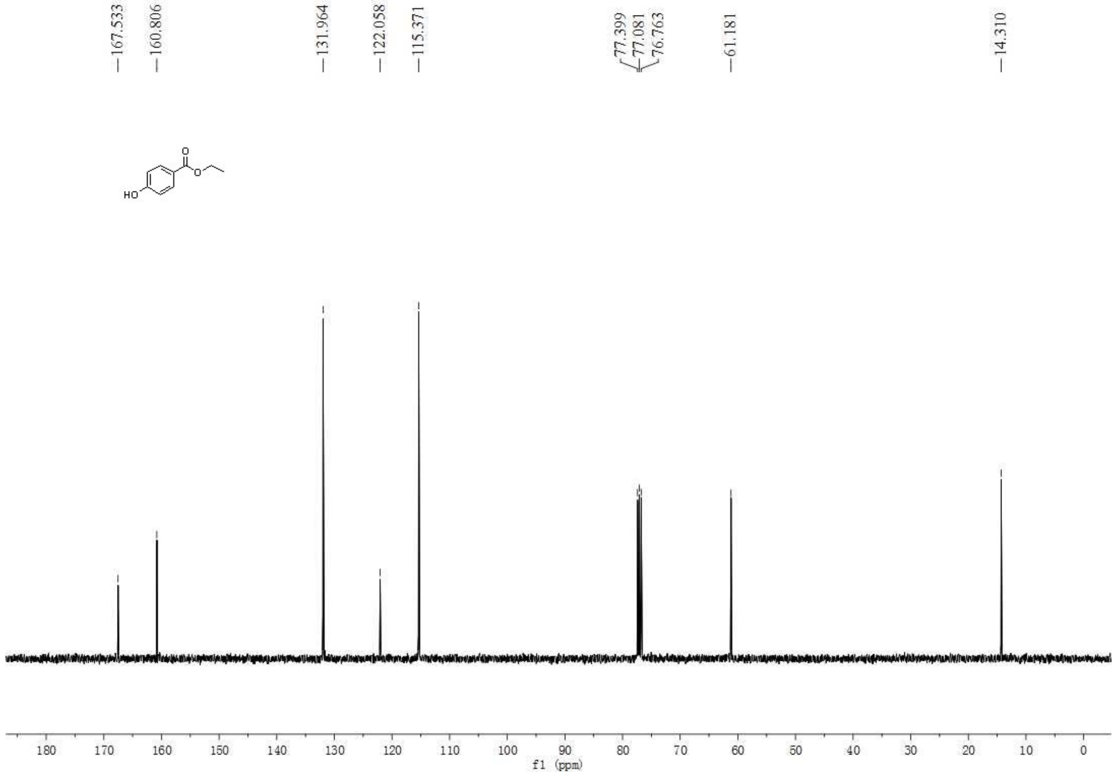
167.533
160.806
131.964
122.058
115.371
77.399
77.081
76.763
61.181
14.310
HO
O
O
f1 (ppm)

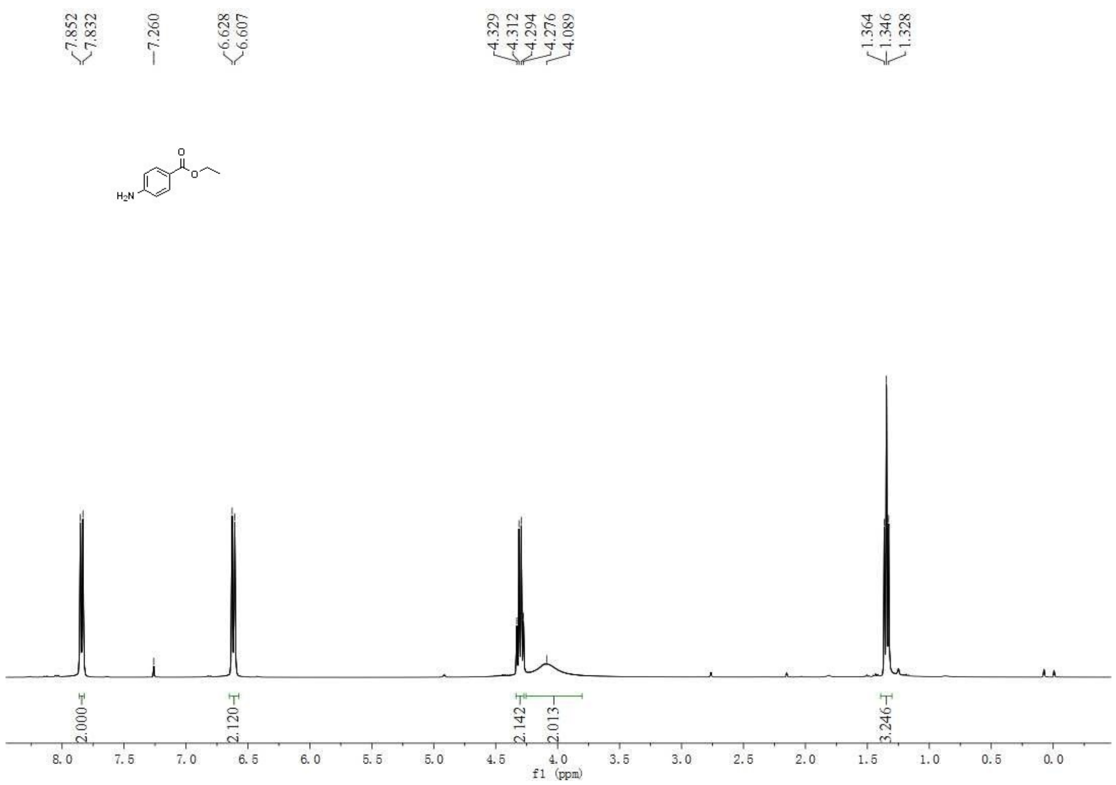
7.852
7.832
7.260
6.628
6.607
4.329
4.312
4.294
4.276
4.089
1.364
1.346
1.328
H2N
O
2.000
2.120
2.142
2.013
3.246
f1 (ppm)

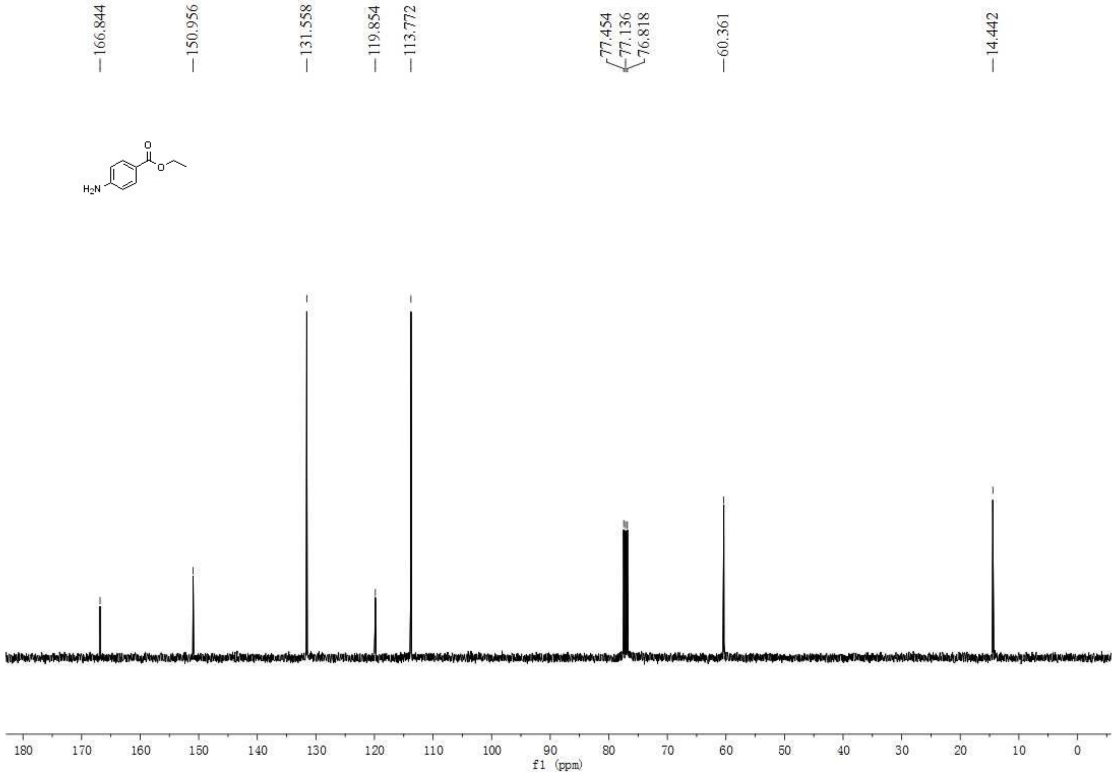
166.844
150.956
131.558
119.854
113.772
77.454
77.136
76.818
60.361
14.442
H2N
O
f1 (ppm)

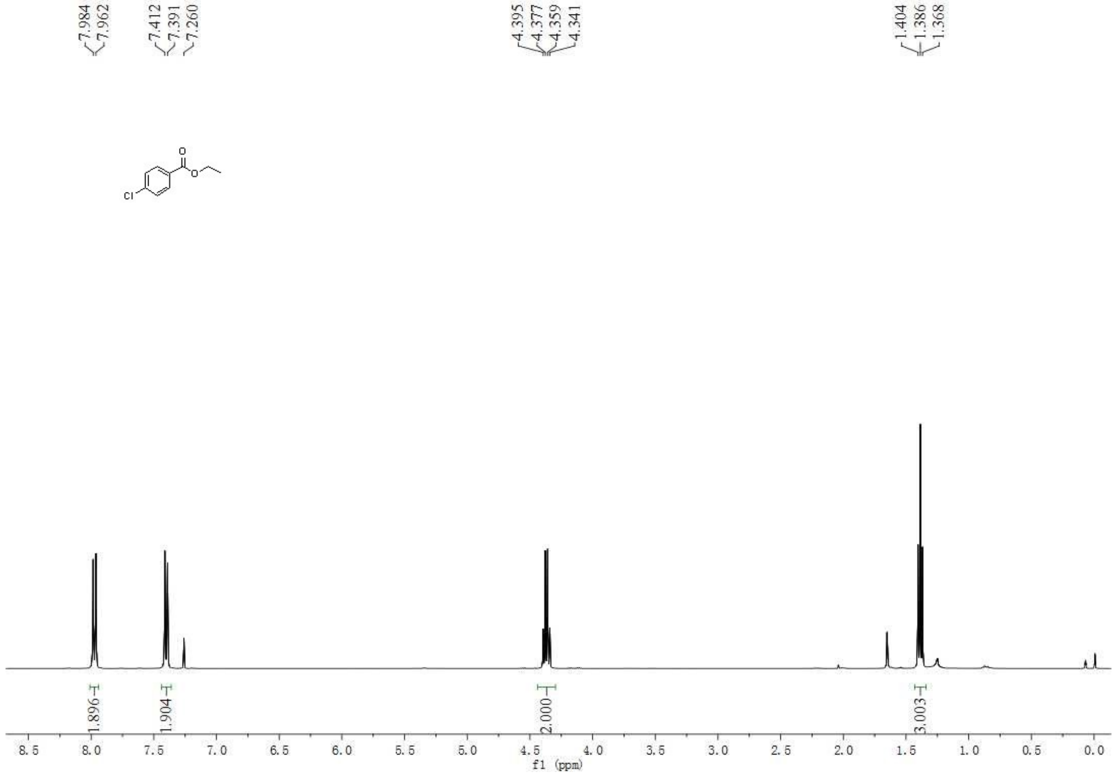
7.984
7.962
7.412
7.391
7.260
4.395
4.377
4.359
4.341
1.404
1.386
1.368
Cl
O
1.896
1.904
2.000
3.003
8.5 8.0 7.5 7.0 6.5 6.0 5.5 5.0 4.5 4.0 3.5 3.0 2.5 2.0 1.5 1.0 0.5 0.0
f1 (ppm)

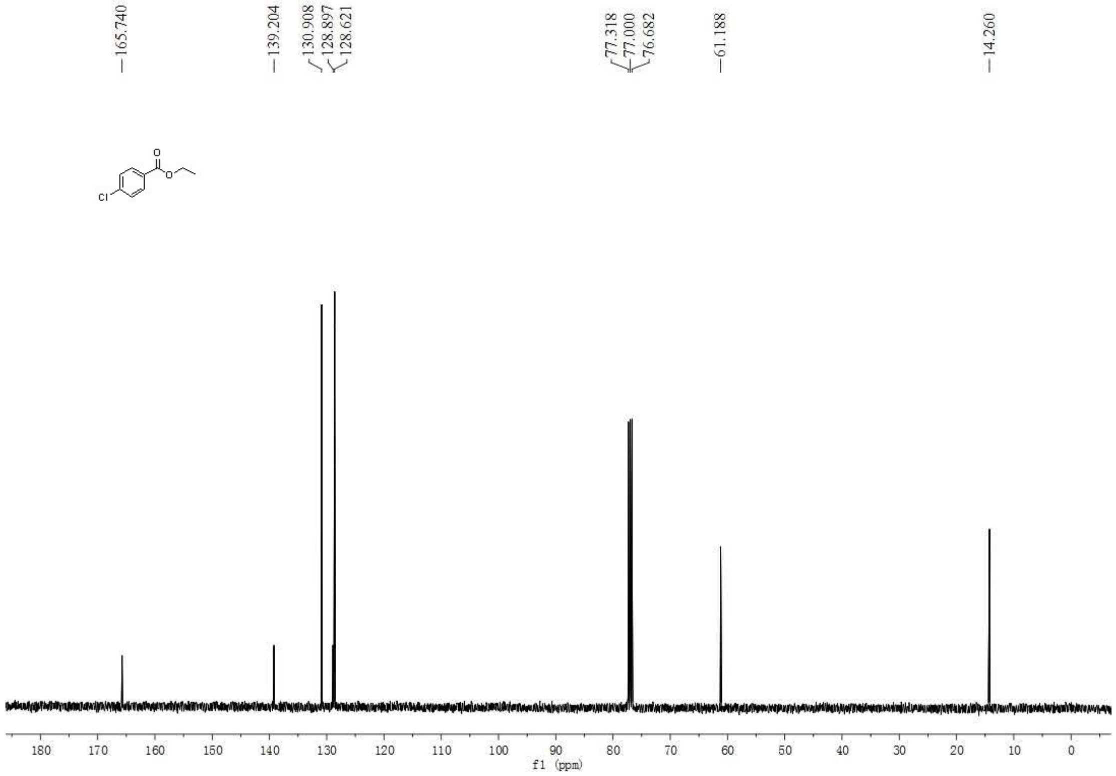
165.740
139.204
130.908
128.897
128.621
77.318
77.000
76.682
61.188
14.260
Cl
O
180 170 160 150 140 130 120 110 100 90 80 70 60 50 40 30 20 10 0
f1 (ppm)

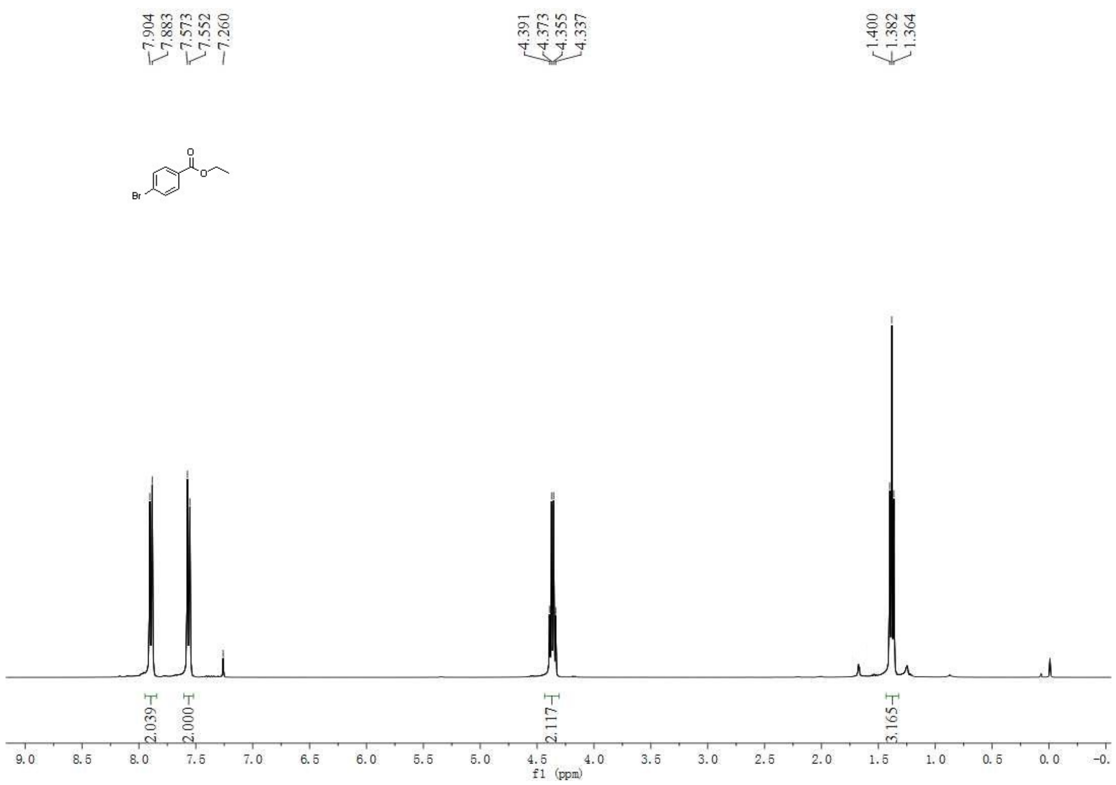
7.904
7.883
7.573
7.552
7.260
4.391
4.373
4.355
4.337
1.400
1.382
1.364
Br
O
O
2.039
2.000
2.117
3.165
9.0 8.5 8.0 7.5 7.0 6.5 6.0 5.5 5.0 4.5 4.0 3.5 3.0 2.5 2.0 1.5 1.0 0.5 0.0
f1 (ppm)

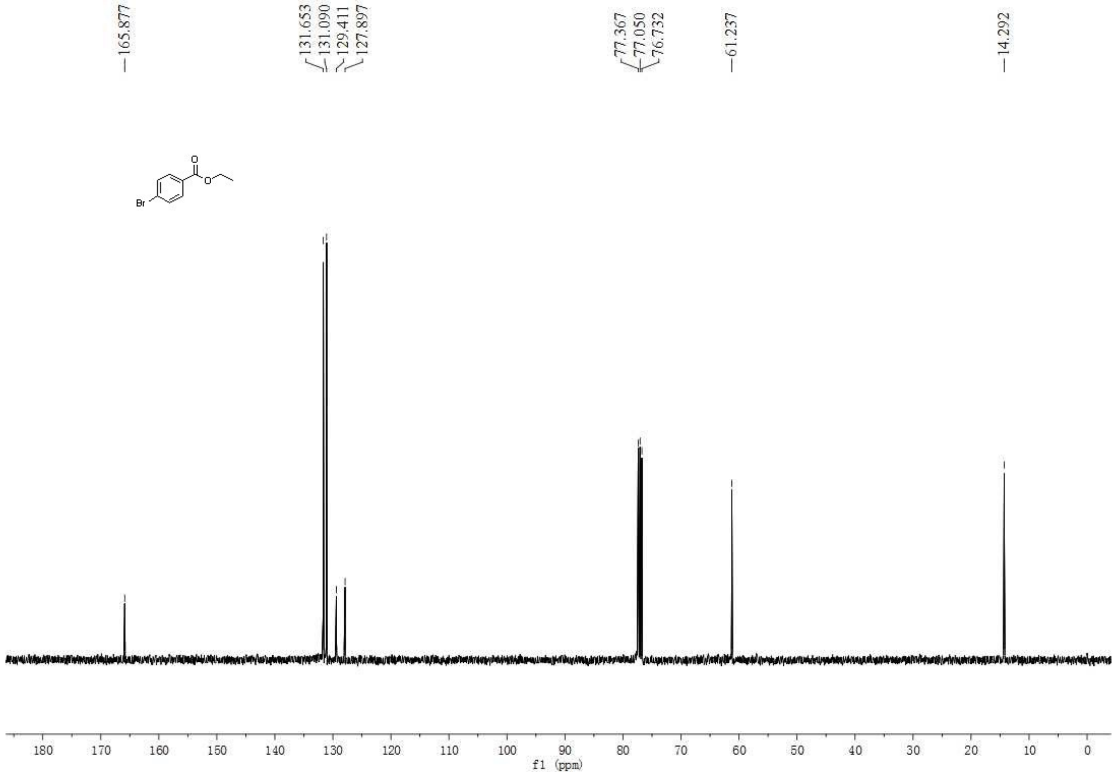
165.877
131.653
131.090
129.411
127.897
77.367
77.050
76.732
61.237
14.292
Br
O
O
180 170 160 150 140 130 120 110 100 90 80 70 60 50 40 30 20 10 0
f1 (ppm)

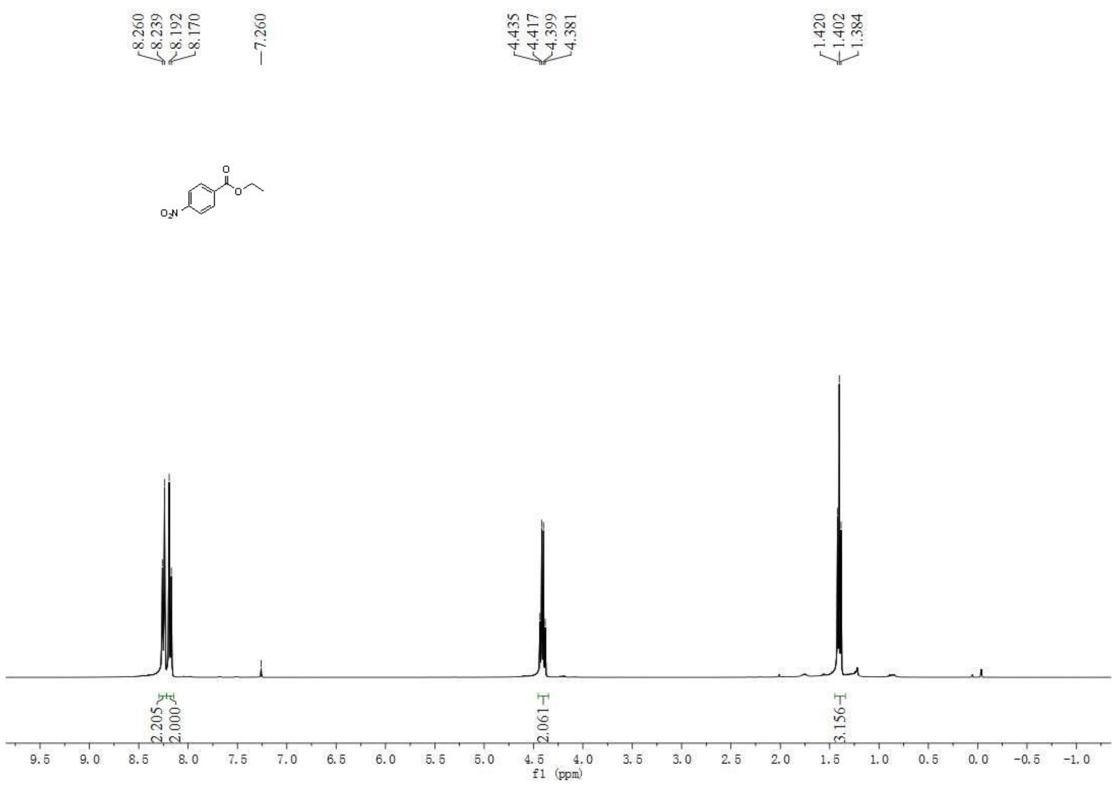
8.260
8.239
8.192
8.170
7.260
4.435
4.417
4.399
4.381
1.420
1.402
1.384
2.205
2.000
2.061
3.156
9.5 9.0 8.5 8.0 7.5 7.0 6.5 6.0 5.5 5.0 4.5 4.0 3.5 3.0 2.5 2.0 1.5 1.0 0.5 0.0 -0.5 -1.0
f1 (ppm)

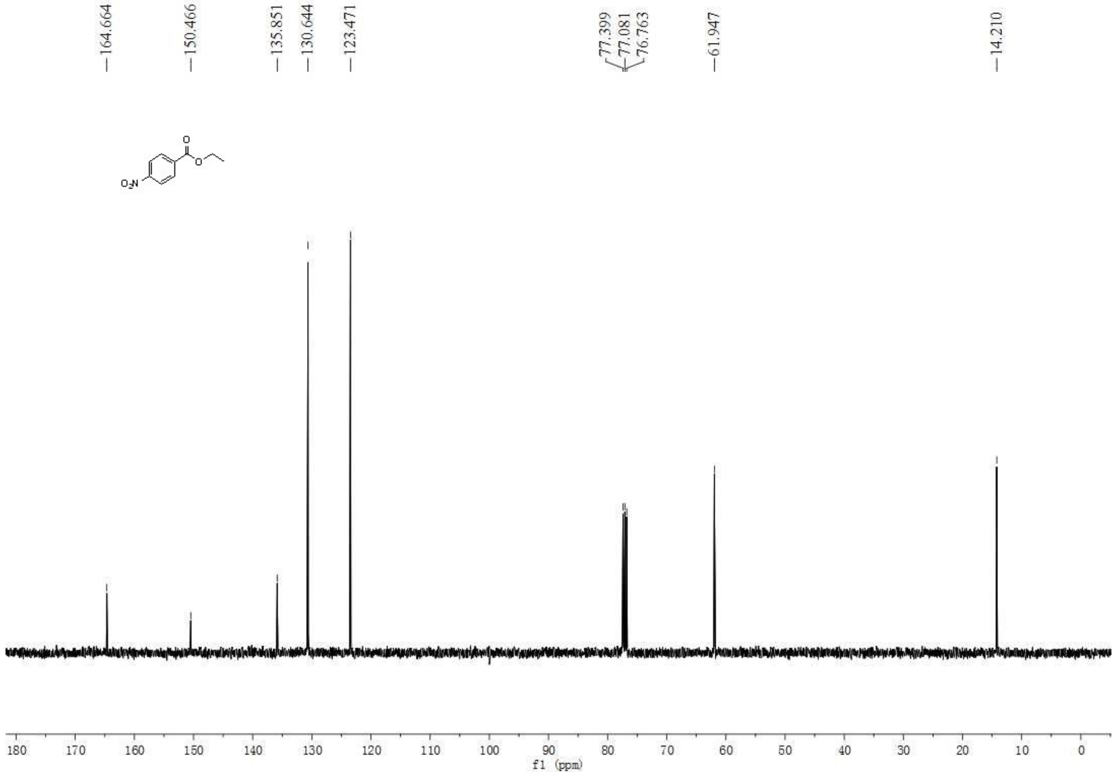
164.664
150.466
135.851
130.644
123.471
77.399
77.081
76.763
61.947
14.210
180 170 160 150 140 130 120 110 100 90 80 70 60 50 40 30 20 10 0
f1 (ppm)

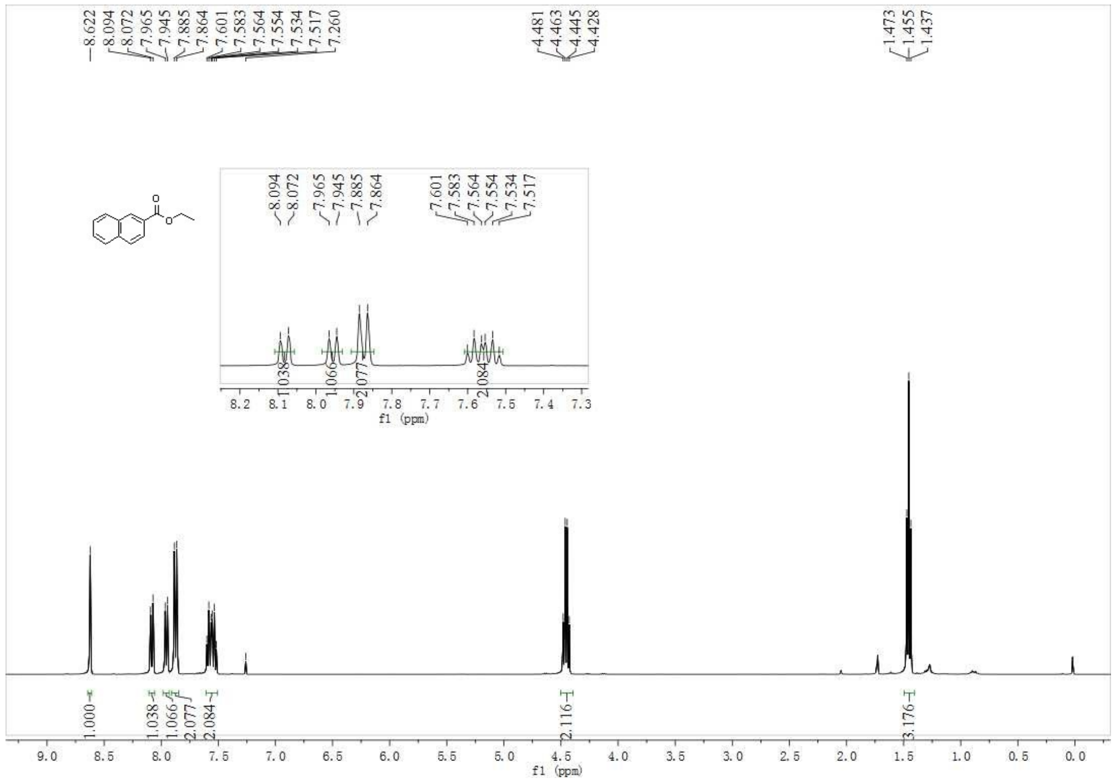
8.622
8.094
8.072
7.965
7.945
7.885
7.864
7.601
7.583
7.564
7.554
7.534
7.517
7.260
4.481
4.463
4.445
4.428
1.473
1.455
1.437
1.000
1.038
1.066
2.077
2.084
2.116
3.176
f1 (ppm)

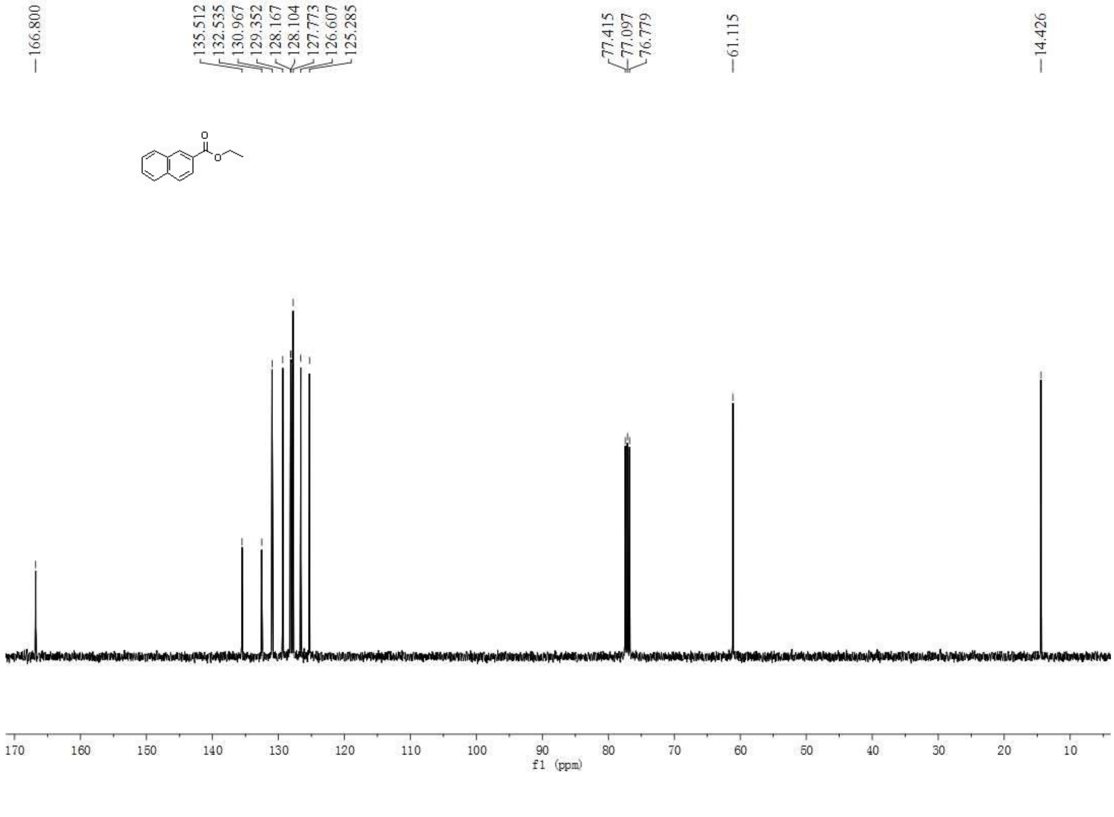
166.800
135.512
132.535
130.967
129.352
128.167
128.104
127.773
126.607
125.285
77.415
77.097
76.779
61.115
14.426
f1 (ppm)

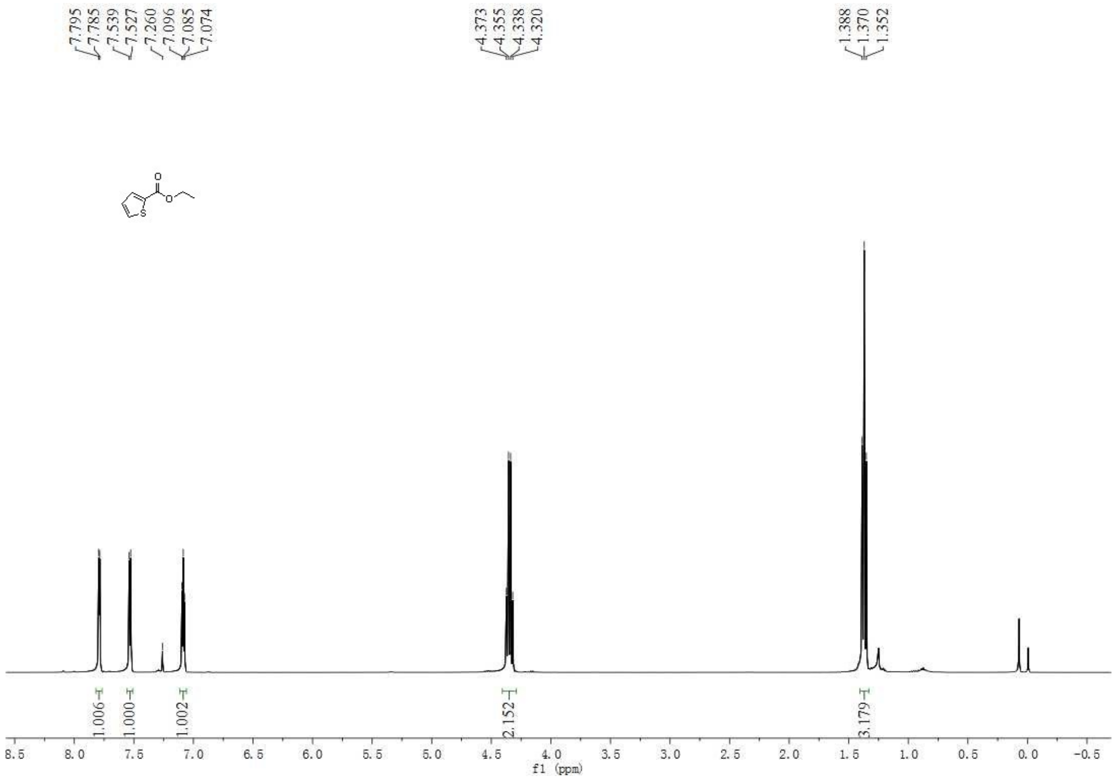
7.795
7.785
7.539
7.527
7.260
7.096
7.085
7.074
4.373
4.355
4.338
4.320
1.388
1.370
1.352
1.006
1.000
1.002
2.152
3.179
8.5 8.0 7.5 7.0 6.5 6.0 5.5 5.0 4.5 4.0 3.5 3.0 2.5 2.0 1.5 1.0 0.5 0.0 -0.5
f1 (ppm)

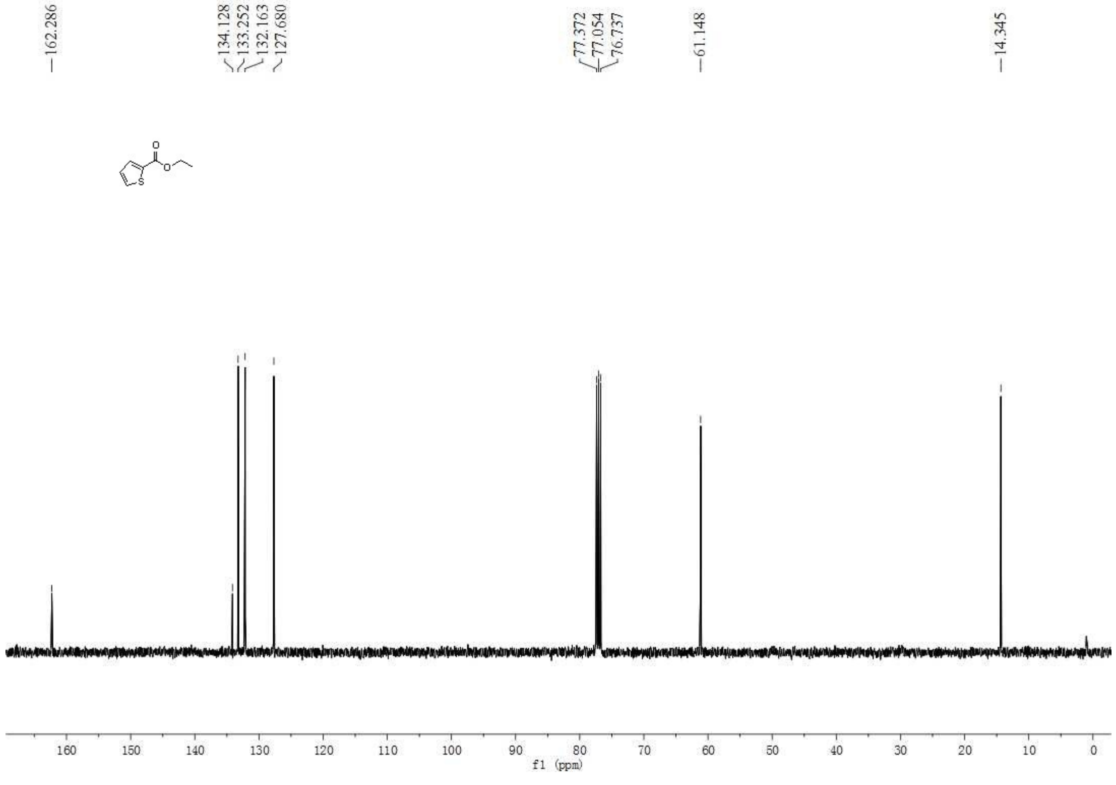
162.286
134.128
133.252
132.163
127.680
77.372
77.054
76.737
61.148
14.345
160 150 140 130 120 110 100 90 80 70 60 50 40 30 20 10 0
f1 (ppm)

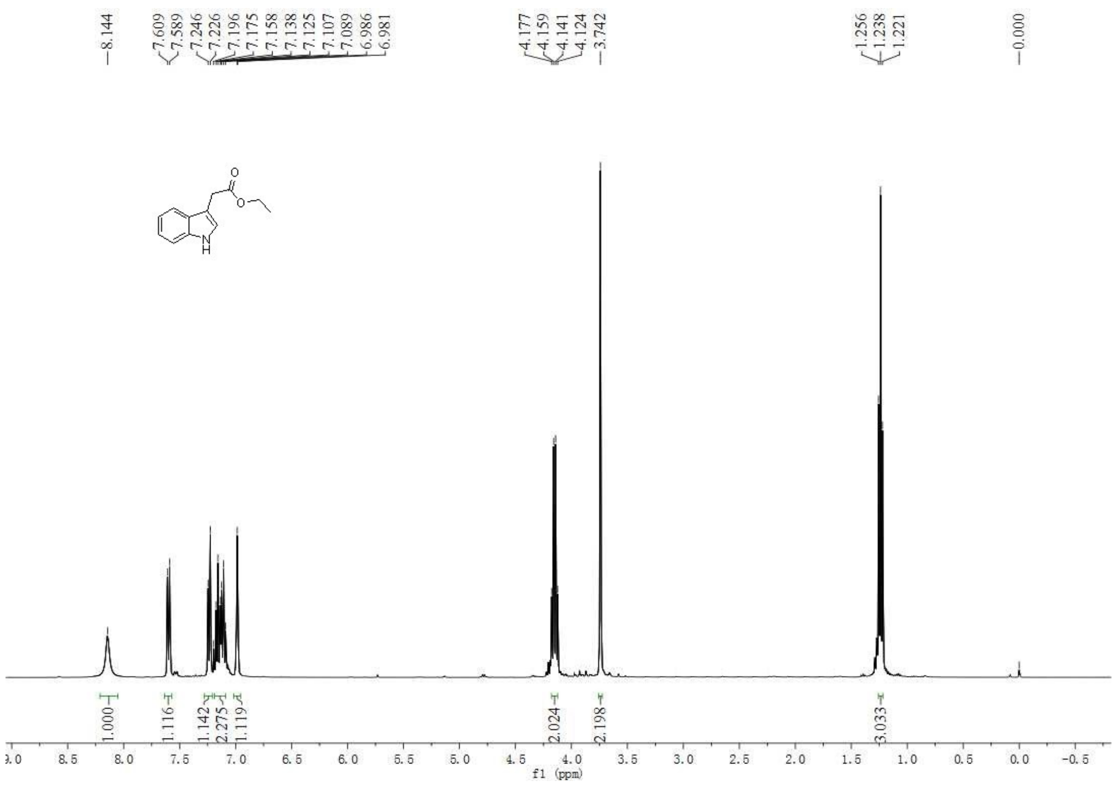
8.144
7.609
7.589
7.246
7.226
7.196
7.175
7.158
7.138
7.125
7.107
7.089
6.986
6.981
4.177
4.159
4.141
4.124
3.742
1.256
1.238
1.221
0.000
O
O
N
H
1.000
1.116
1.142
2.275
1.119
2.024
2.198
3.033
f1 (ppm)

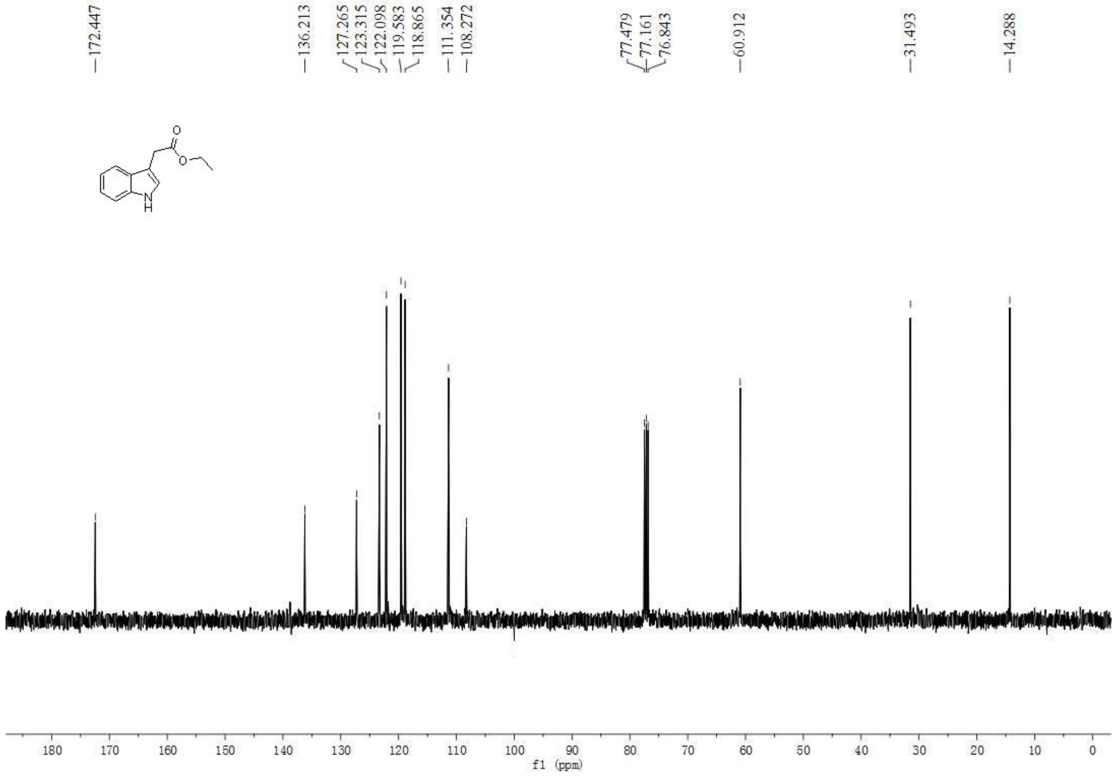
172.447
136.213
127.265
123.315
122.098
119.583
118.865
111.354
108.272
77.479
77.161
76.843
60.912
31.493
14.288
O
O
N
H
f1 (ppm)

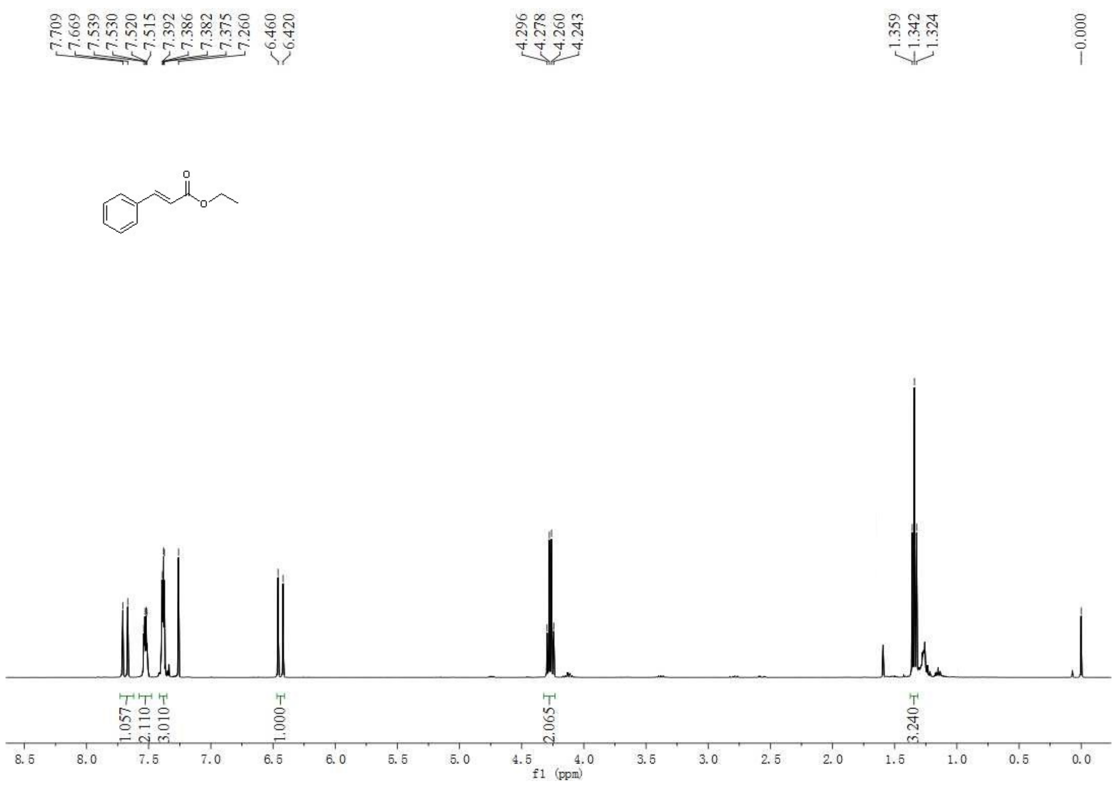
7.709
7.669
7.539
7.530
7.520
7.515
7.392
7.386
7.382
7.375
7.260
6.460
6.420
4.296
4.278
4.260
4.243
1.359
1.342
1.324
0.000
O
O
1.057
2.110
3.010
1.000
2.065
3.240
8.5 8.0 7.5 7.0 6.5 6.0 5.5 5.0 4.5 4.0 3.5 3.0 2.5 2.0 1.5 1.0 0.5 0.0
f1 (ppm)

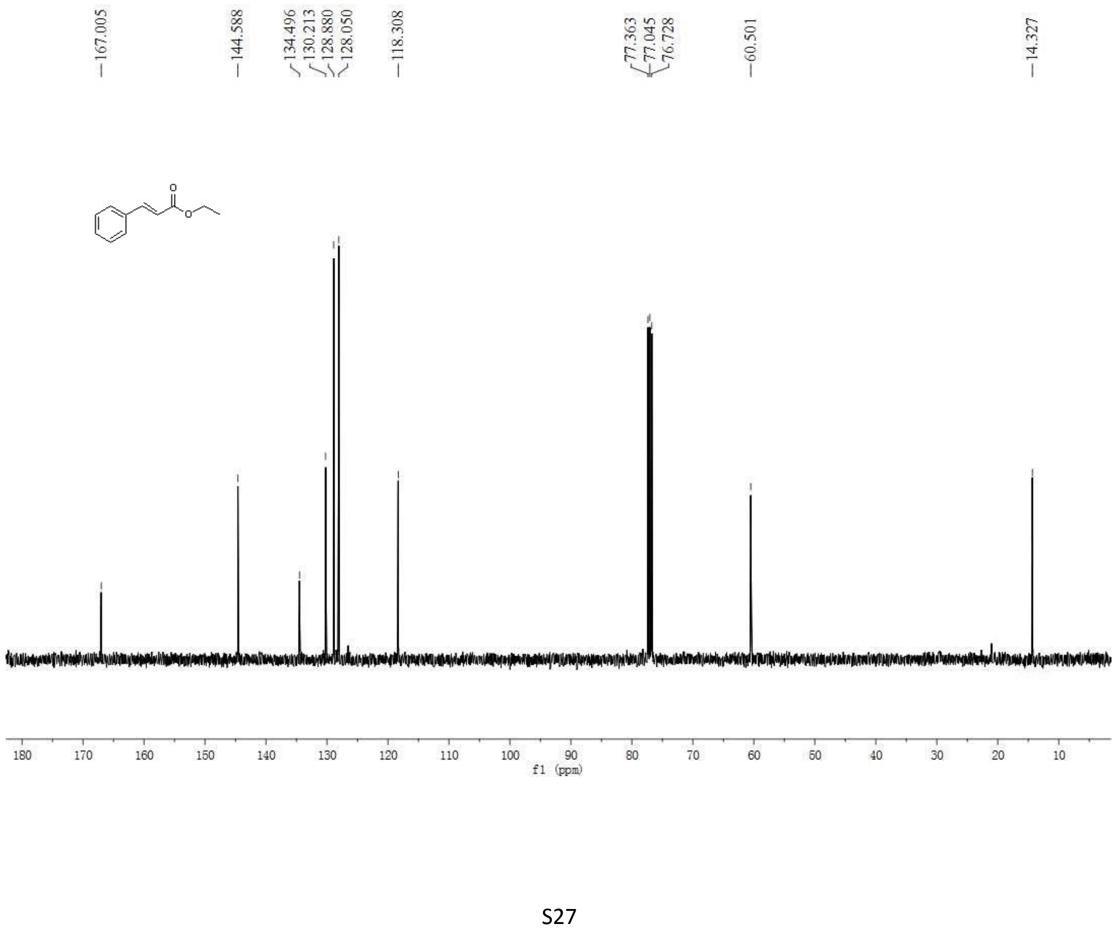
167.005
144.588
134.496
130.213
128.880
128.050
118.308
77.363
77.045
76.728
60.501
14.327
O
O
180 170 160 150 140 130 120 110 100 90 80 70 60 50 40 30 20 10
f1 (ppm)

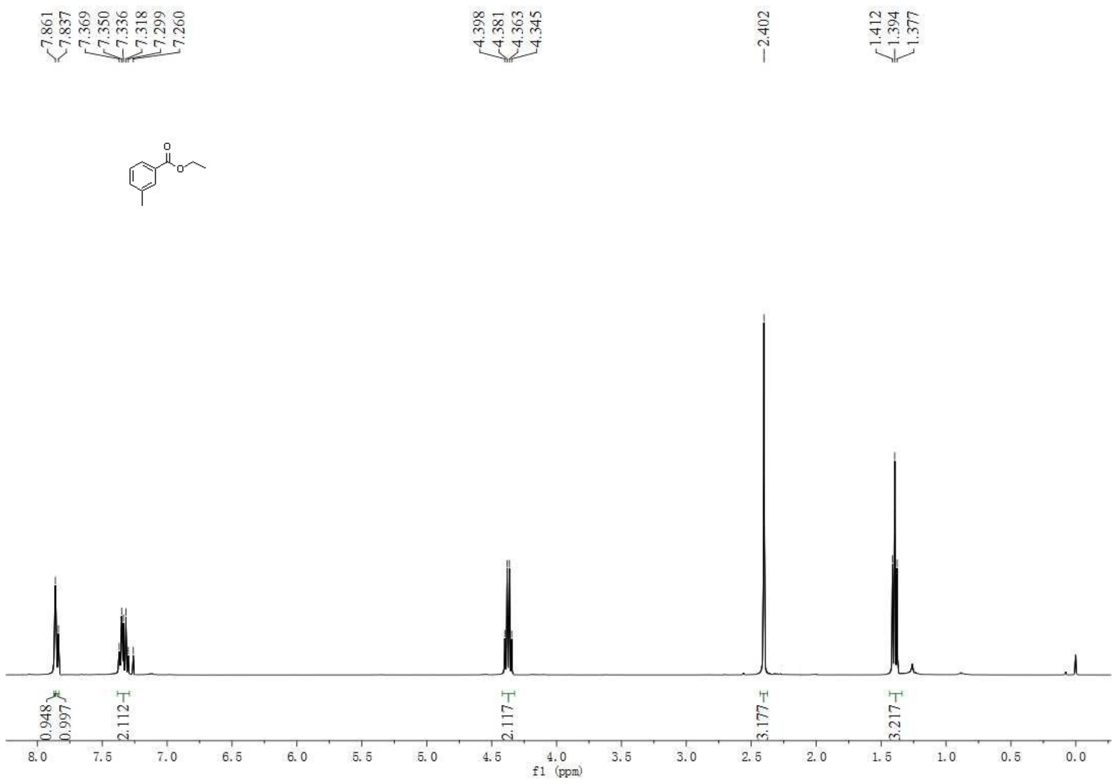

7.861
7.837
7.369
7.350
7.336
7.318
7.299
7.260
4.398
4.381
4.363
4.345
2.402
1.412
1.394
1.377
O
O
0.948
0.997
2.112
2.117
3.177
3.217
8.0 7.5 7.0 6.5 6.0 5.5 5.0 4.5 4.0 3.5 3.0 2.5 2.0 1.5 1.0 0.5 0.0
f1 (ppm)


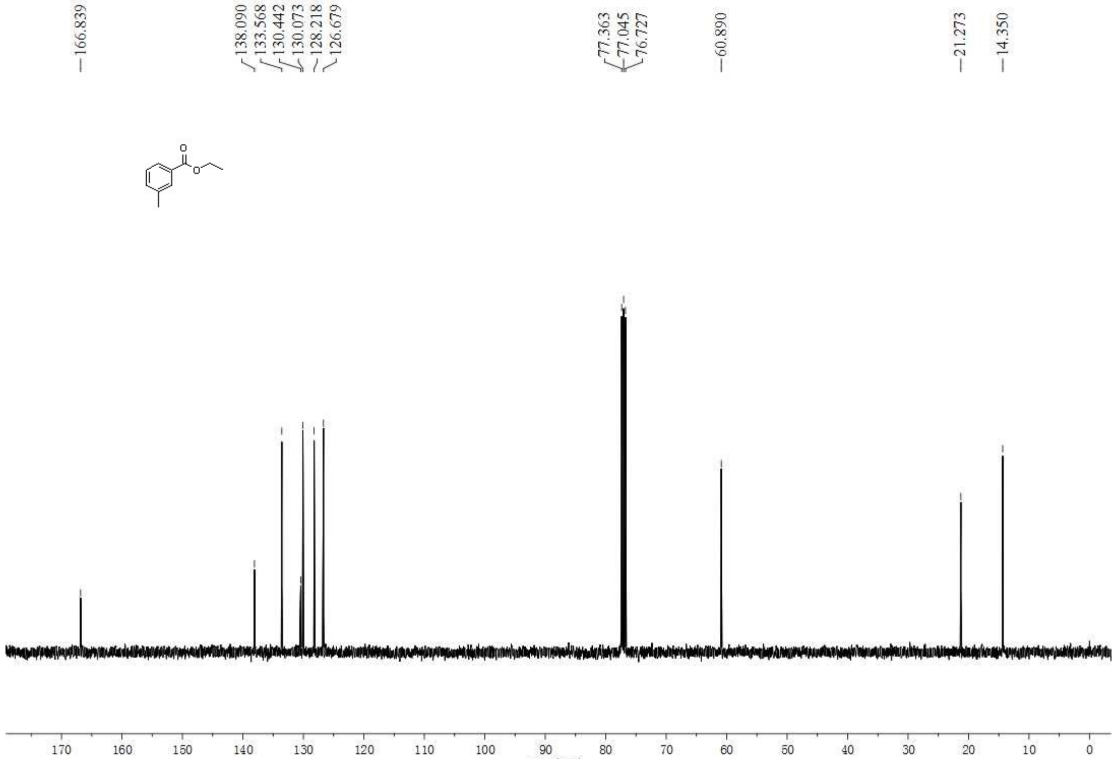

166.839
138.090
133.568
130.442
130.073
128.218
126.679
77.363
77.045
76.727
60.890
21.273
14.350
O
O
170 160 150 140 130 120 110 100 90 80 70 60 50 40 30 20 10 0
f1 (ppm)

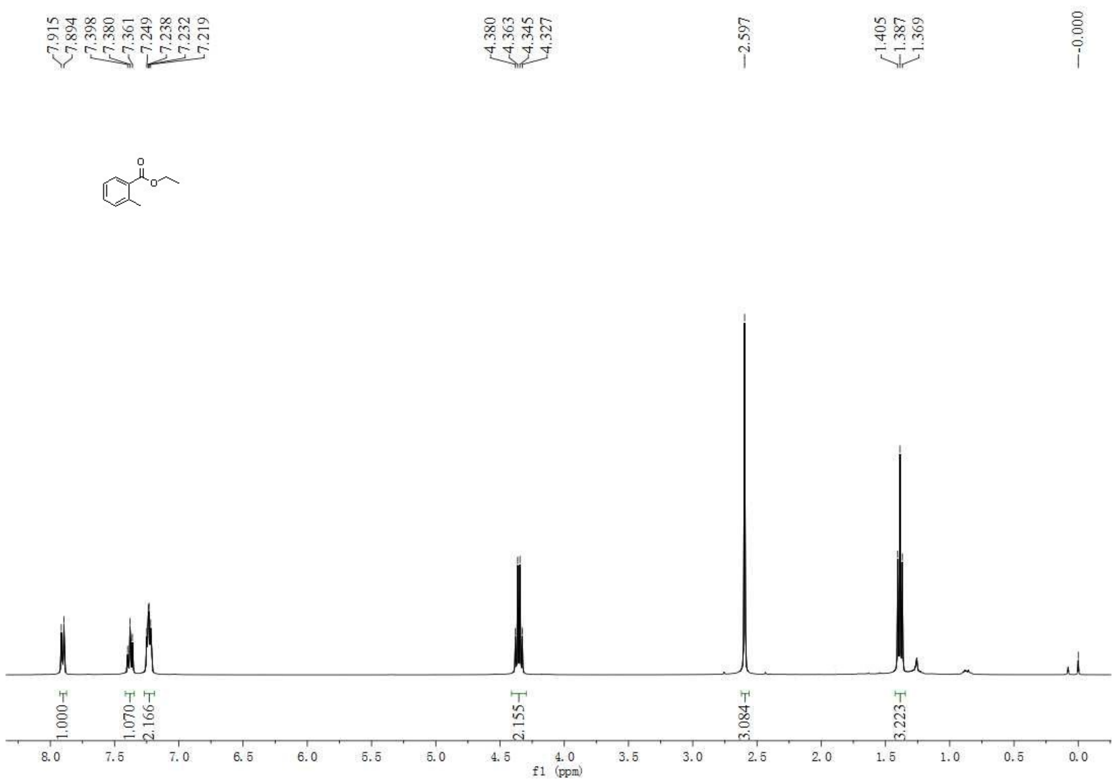

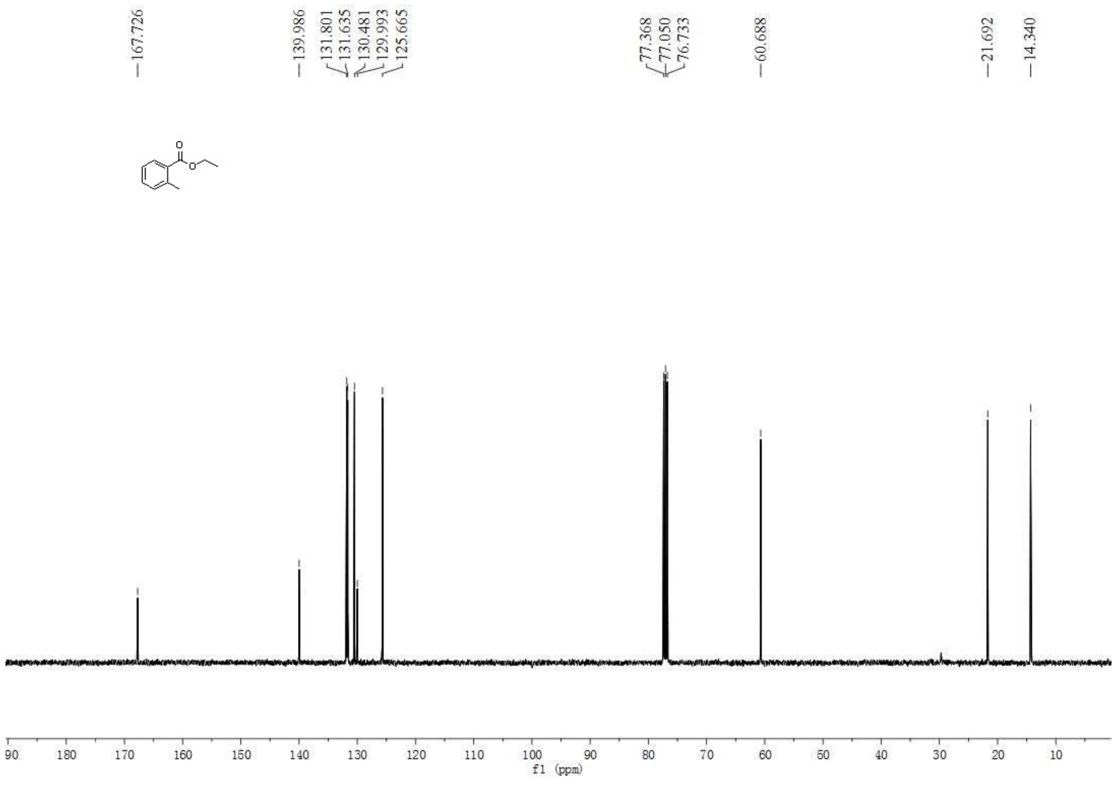

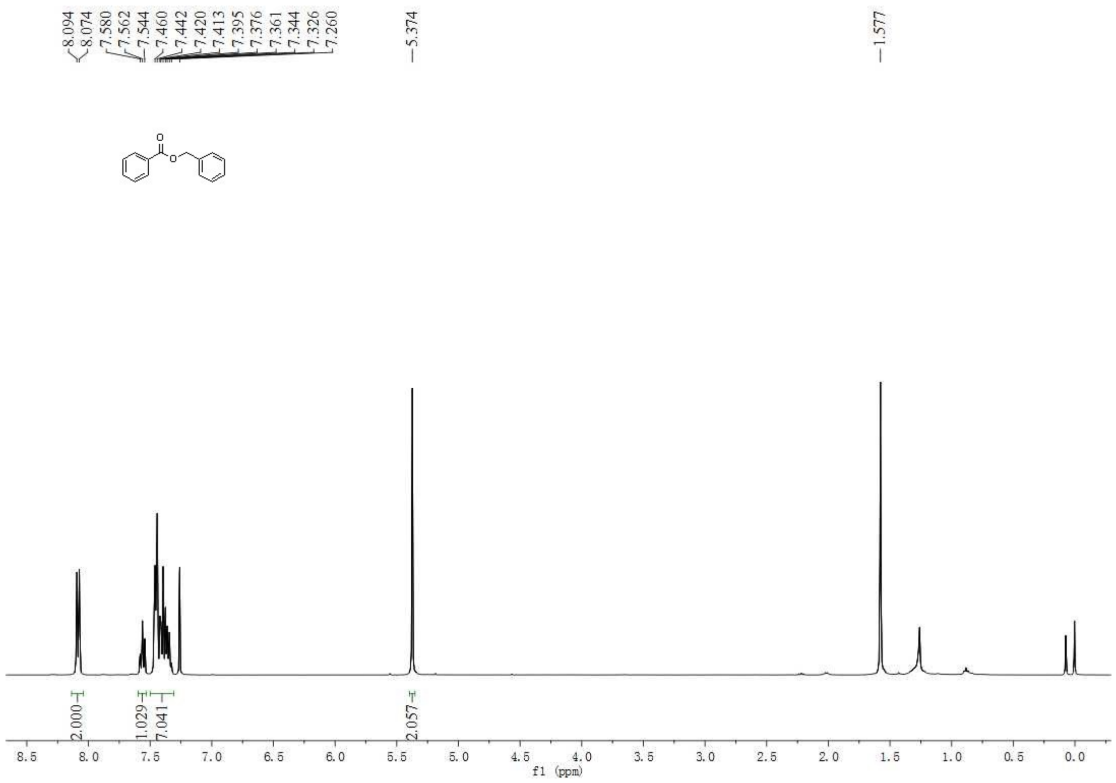
8.094
8.074
7.580
7.562
7.544
7.460
7.442
7.420
7.413
7.395
7.376
7.361
7.344
7.326
7.260
5.374
1.577
2.000
1.029
7.041
2.057
f1 (ppm)

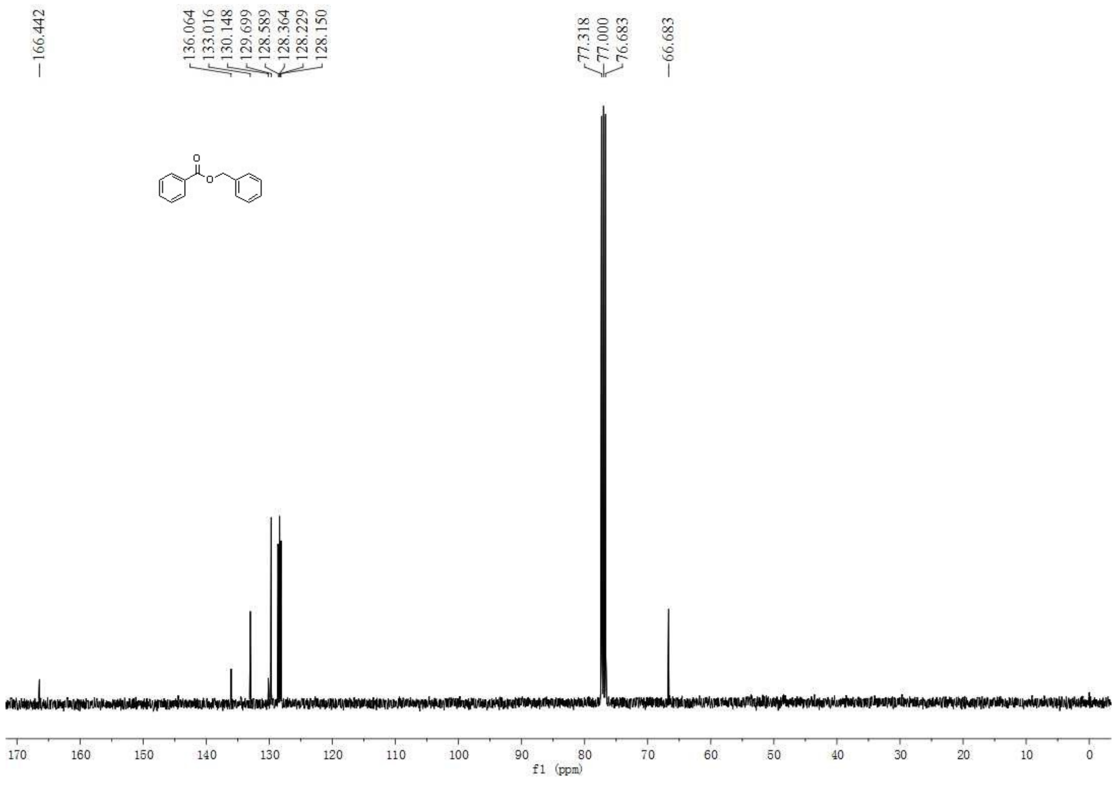
166.442
136.064
133.016
130.148
129.699
128.589
128.364
128.229
128.150
77.318
77.000
76.683
66.683
f1 (ppm)

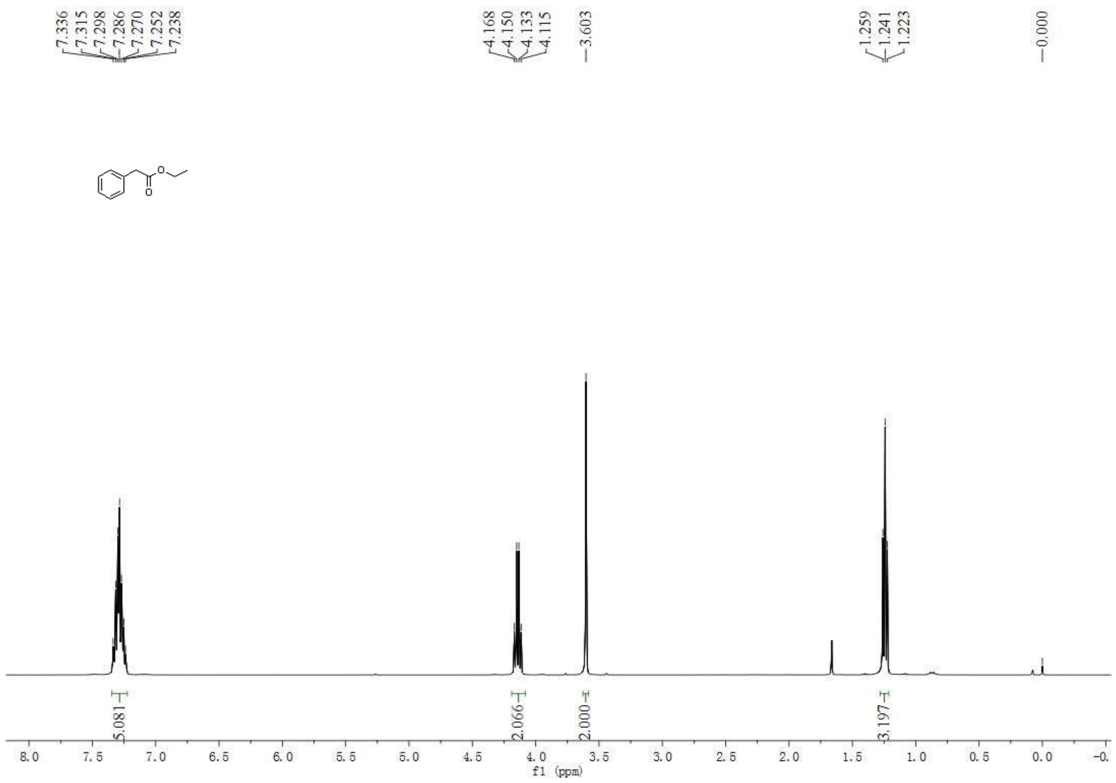
7.336
7.315
7.298
7.286
7.270
7.252
7.238
4.168
4.150
4.133
4.115
3.603
1.259
1.241
1.223
0.000
5.081
2.066
2.000
3.197
8.0 7.5 7.0 6.5 6.0 5.5 5.0 4.5 4.0 3.5 3.0 2.5 2.0 1.5 1.0 0.5 0.0
f1 (ppm)

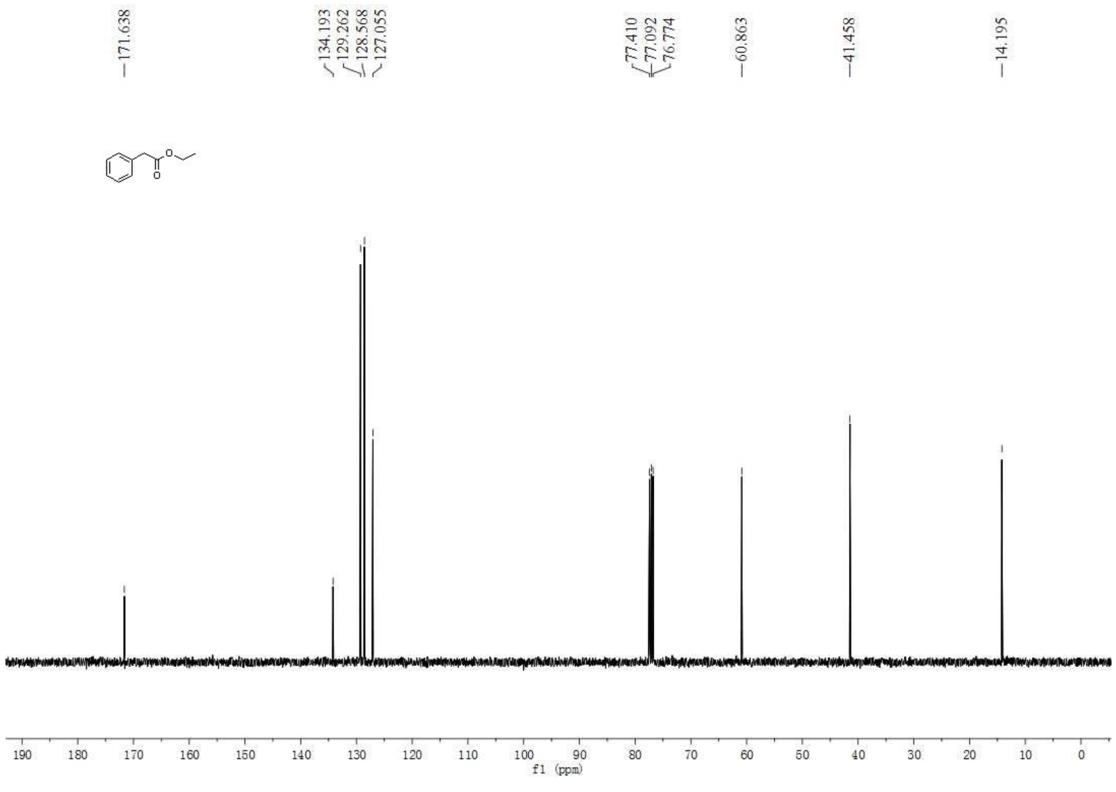
171.638
134.193
129.262
128.568
127.055
77.410
77.092
76.774
60.863
41.458
14.195
190 180 170 160 150 140 130 120 110 100 90 80 70 60 50 40 30 20 10 0
f1 (ppm)

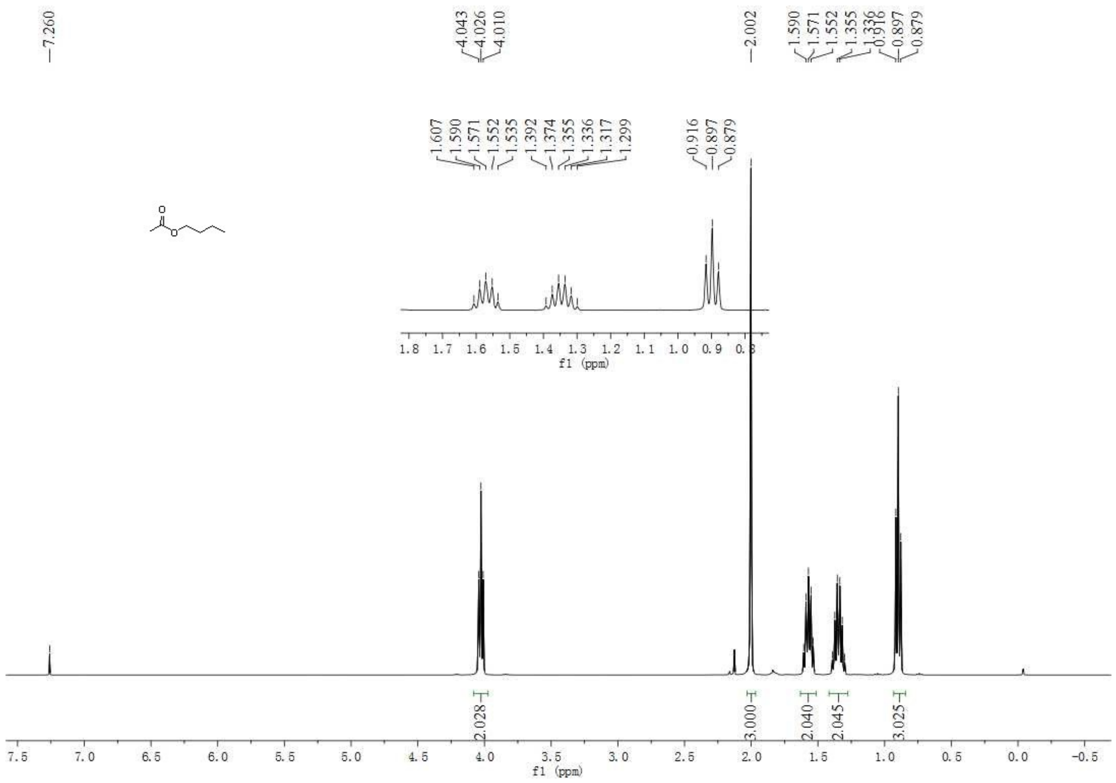
7.260
4.043
4.026
4.010
2.002
1.590
1.571
1.552
1.355
1.336
0.916
0.897
0.879
1.607
1.590
1.571
1.552
1.535
1.392
1.374
1.355
1.336
1.317
1.299
0.916
0.897
0.879
f1 (ppm)
2.028
3.000
2.040
2.045
3.025
f1 (ppm)

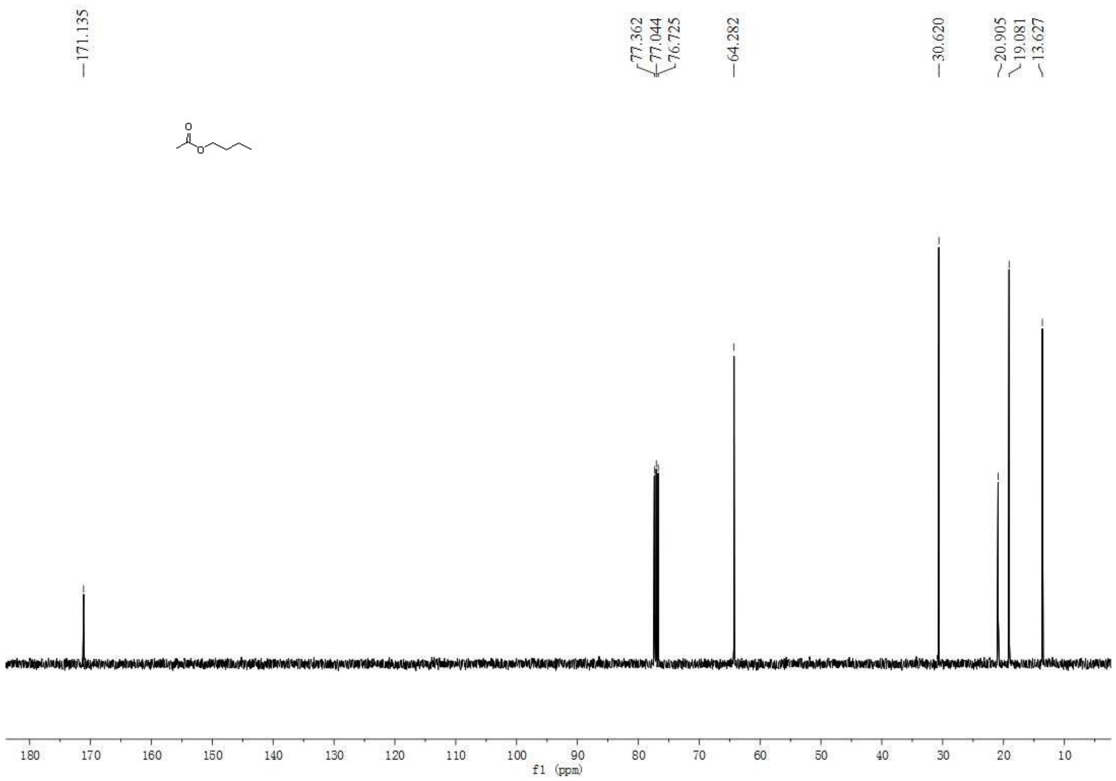
171.135
77.362
77.044
76.725
64.282
30.620
20.905
19.081
13.627
f1 (ppm)

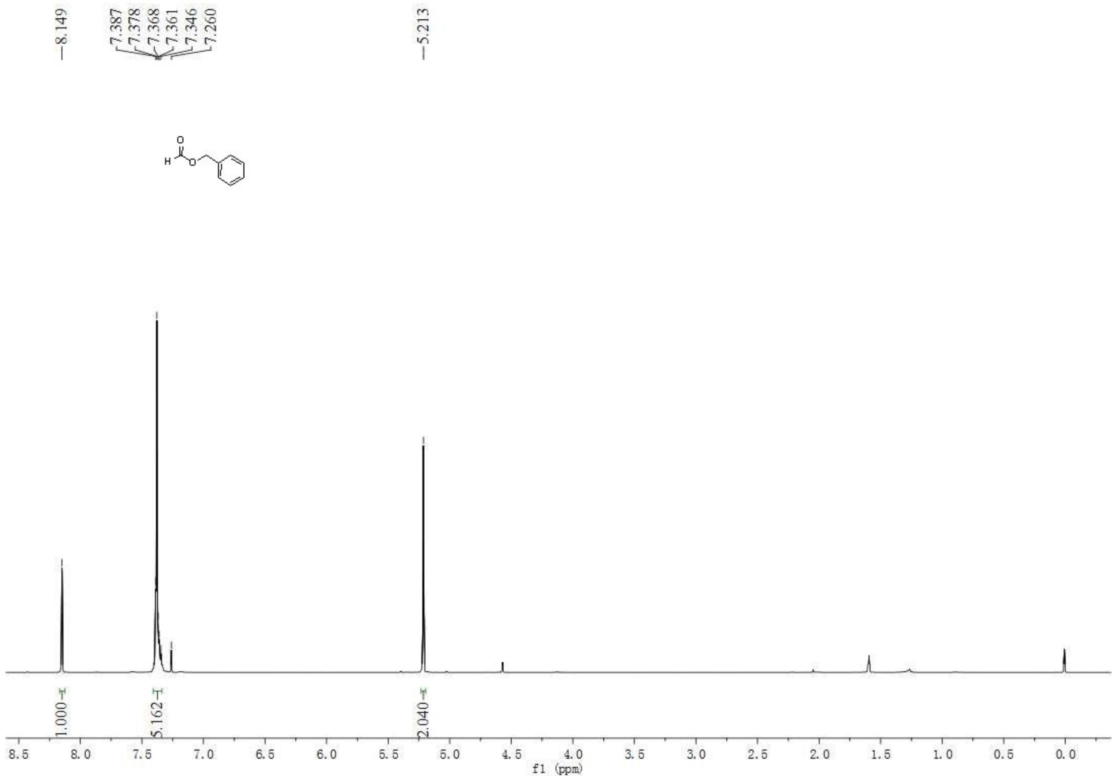
8.149
7.387
7.378
7.368
7.361
7.346
7.260
5.213
1.000
5.162
2.040
f1 (ppm)

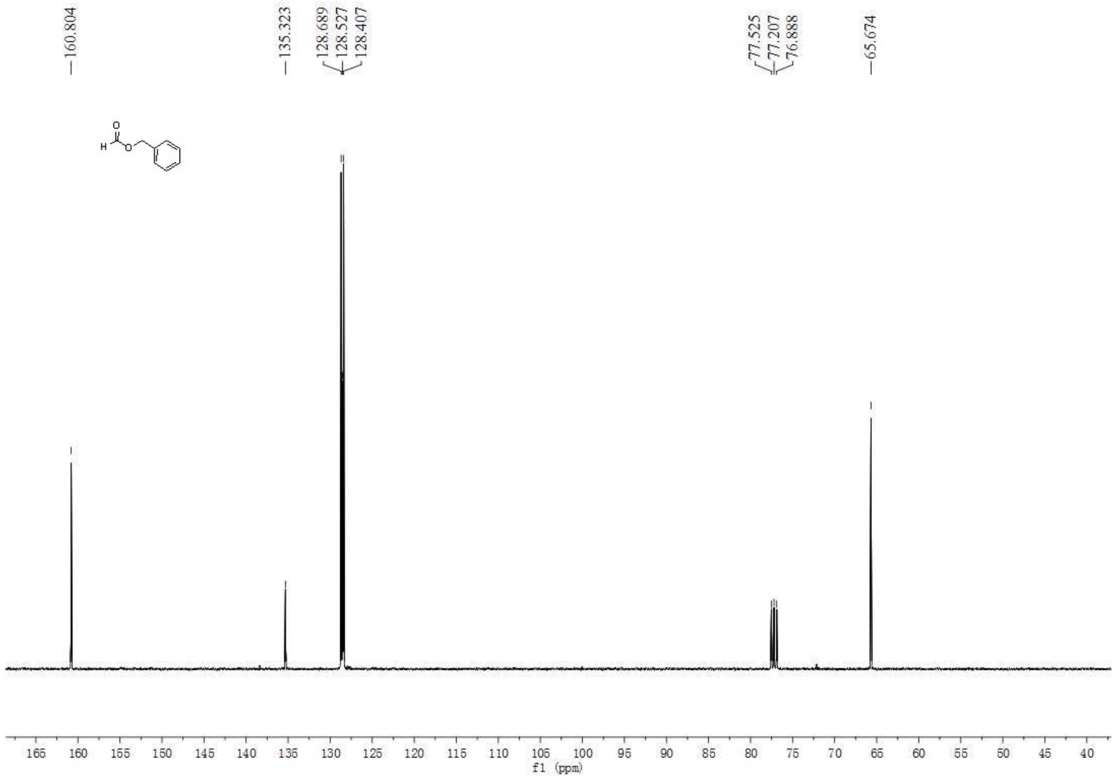
160.804
135.323
128.689
128.527
128.407
77.525
77.207
76.888
65.674
f1 (ppm)
